# Stereoselective Synthesis and Biological Evaluation of Perhydroquinoxaline-Based κ Receptor Agonists

**DOI:** 10.3390/ijms26030998

**Published:** 2025-01-24

**Authors:** Jonathan Hoffmann, Dirk Schepmann, Constantin Daniliuc, Marcel Bermudez, Bernhard Wünsch

**Affiliations:** 1 Institut für Pharmazeutische und Medizinische Chemie, Universität Münster, Corrensstraße 48, D-48149 Münster, Germany; jonathan_hoffmann@outlook.de (J.H.); dirk.schepmann@uni-muenster.de (D.S.);; 2 Organisch-Chemisches Institut, Universität Münster, Corrensstraße 40, D-48149 Münster, Germany; constantin.daniliuc@uni-muenster.de; 3 GRK 2515, Chemical Biology of Ion Channels (Chembion), Universität Münster, Corrensstr. 48, D-48149 Münster, Germany

**Keywords:** κ receptor agonists, ethylenediamines, perhydroquinoxalines, *trans*-configuration, pseudochirality, stereoselective synthesis, κ receptor affinity, docking

## Abstract

The hydroxylated perhydroquinoxaline **14** was designed by conformational restriction of the prototypical κ receptor agonist U-50,488 and the introduction of an additional polar group. The synthesis of **14** comprised ten reaction steps starting from diethyl 3-hydroxyglutarate (**4**). The first key step was the diastereoselective establishment of the tetrasubstituted cyclohexane **7** by the reaction of dialdehyde **6** with benzylamine and nitromethane. The piperazine ring was annulated by the reaction of silyloxy-substituted cyclohexanetriamine **8** with dimethyl oxalate. The pharmacophoric structural elements characteristic for κ receptor agonists were finally introduced by functional group modifications. The structure including the relative configuration of the tetrasubstituted cyclohexane derivative (2*r*,5*s*)-**7a** and the perhydroquinoxaline **9** was determined unequivocally by X-ray crystal structure analysis. The hydroxylated perhydroquinoxaline **14** showed moderate κ receptor affinity (*K*_i_ = 599 nM) and high selectivity over μ, δ, σ_1_, and σ_2_ receptors. An ionic interaction between the protonated pyrrolidine of **14** and D138 of κ receptor anchors **14** in the κ receptor binding pocket.

## 1. Introduction

The group of opioid receptors is differentiated into four subtypes: µ receptors (MOP), δ receptors (DOP), κ receptors (KOP), and nociceptin receptors (NOP). These receptors are termed according to their prototypical agonists morphine (μ receptor), ketazocine (κ receptor), nociceptin (NOP), or the tissue where the receptor was first detected (vas *d*eferens, δ receptor) [1,2]. While the neuropeptide nociceptin (orphanin FQ, heptadecapeptide) activating NOP is a potent antianalgesic compound [3], the activation of μ, δ, and κ receptors leads to strong analgesia [4,5].

In contrast to the analgesic effects induced by the activation of μ and δ receptors, the analgesic effects produced by κ opioid receptor agonists are not accompanied by severe side effects, such as respiratory depression, euphoria, addiction or epileptic seizures [6,7]. In addition to the treatment of pain, κ receptor agonists can be used for the treatment of inflammatory itching skin diseases. The morphinan-based κ agonist nalfurafine has been approved in Japan for the treatment of pruritus [8,9,10]. Moreover, it has been shown that the endogenous opioid system is involved in processes such as inflammatory neurodegeneration, which finally could lead to multiple sclerosis (MS) or experimental autoimmune encephalomyelitis (EAE). In particular, κ agonists are able to regulate cytokine production and immune cell activation, resulting in beneficial effects in these neuroinflammatory and neurodegenerative disorders [11,12,13,14].

The four opioid receptor subtypes are members of class A (rhodopsin-like family) of G protein-coupled receptors and recruit the G-protein Gi/Go upon activation. In 2010, the κ receptor was crystallized in the inactive state together with the antagonist JDTic. Six years later, crystals of the κ receptor in the activated conformation with the agonist MP1104 and a stabilizing nanobody were obtained. The structures of both complexes were determined by X-ray crystal structure analysis. The binding of MP1104 is mainly driven by an ionic interaction between its protonated amino moiety and D138 of κ receptor, a key interaction that is observed for all orthosteric κ receptor agonists [15,16,17].

Agonists at κ receptors belong to four different compound classes: peptides derived from the endogenous κ agonist dynorphin A [18], morphinans and benzomorphans derived from morphine, N-free terpenoids (e.g., salvinorin A) [19], and ethylenediamines. High κ agonistic activity and selectivity over other opioid receptors is achieved by embedding one N-atom of the ethylenediamine substructure into a pyrrolidine ring and attaching the 2-(3,4-dichlorophenyl)acetyl moiety to the other N-atom (arylacetamide substructure) [20].

**Figure 1:**
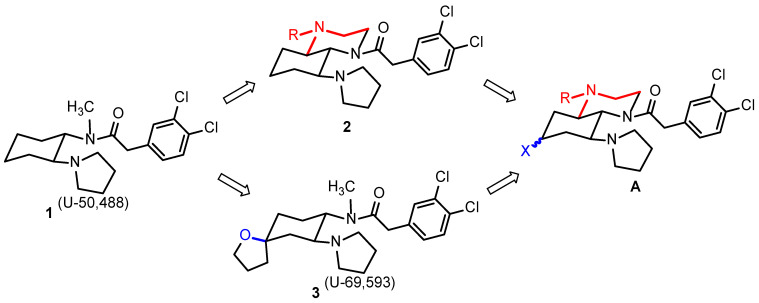
Development of perhydroquinoxaline-based κ receptor agonists **A** starting with the prototypical κ agonist U-55,0488 (**1**) via the κ agonists **2** and U-69,593 (**3**). The new κ receptor agonists **A** should contain both the conformationally restricted perhydroquinoxaline core structure of **2** (red) and the additional O-substituent of **3** (blue).

The first synthetic κ receptor agonist belonging to the ethylenediamine compound class is U-50,488 (**1**) [20,21] (Figure 1). In our radioligand receptor binding assay, U-50,488 revealed a *K*_i_-value of 0.34 nM. Recently, we have connected the *N*-methyl group of U-50,488 with the cyclohexane ring, which led to the perhydroquinoxaline-based κ receptor agonists **2**. As example, compound **2a** bearing a benzyl moiety (R = CH_2_C_6_H_5_) showed a *K*_i_ value at κ receptor of 9.4 nM [22]. The introduction of a tetrahydrofuran ring in the 4-position of the cyclohexane ring of U-50,488 resulted in the very potent spirocyclic κ agonist U-69,583 (**3**, *K*_i_ = 0.88 nM), which is used as a radioligand in receptor binding studies [23,24] (Figure 1).

Herein, we report a combination of both variations in one molecule **A**, i.e., defined orientation of the 2-(3,4-dicholorophenyl)acetyl side chain by connecting the N-methyl moiety with the cyclohexane ring (Figure 1 red) and the introduction of a polar O-substituent in the 4-position of the cyclohexane ring (Figure 1 blue).

## 2. Results and Discussion

### 2.1. Synthesis

**Scheme 1:**
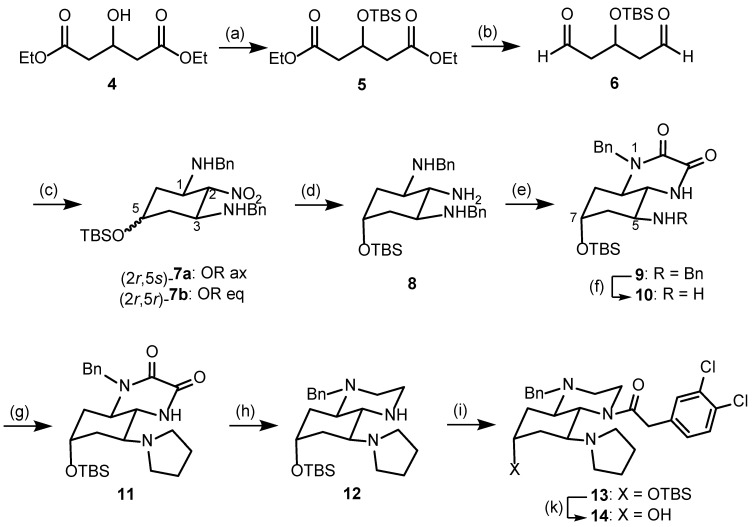
Synthesis of tetrahydroquinoxaline-based κ receptor agonist **14**. Reagents and reaction conditions: (a) ^t^BuMe_2_SICl, imidazole, CH_2_Cl_2_, rt, 24 h, and 92%. (b) DIBAL-H, toluene, −78 °C, 60 min, and 97%. (c) H_3_C-NO_2_, CH_2_Cl_2_, BnNH_2_, 0 °C, 1 h, then rt, 16 h, 85%, and ratio (2*r*,5*s*)-**7a**: (2*r*,5*r*)-**7b** = 70:30; recrystallization provided pure (2*r*,5*s*)-**7a**. (d) (2*r*,5*s*)-**7a**, Zn, THF, NH_4_Cl, 60 °C, 24 h, and 98%. (e) H_3_CO_2_CCO_2_CH_3_, CH_3_OH, reflux, 24 h, and 61%. (f) H_2_ (5 bar), Pd/C, CH_3_OH, rt, 48 h, and 49%. (g) ICH_2_CH_2_CH_2_CH_2_II, Na_2_CO_3_, THF, reflux, 4 d, and 73%. (h) LiAlH_4_, AlCl_3_, THF, then addition of **11**, 0 °C, 45 min, then rt, and 20 min. (i) 2-(3,4-Dichlorophenyl))acetyl chloride, CH_2_Cl_2_, rt, 18 h, and 75% related to **11**. (k) Bu_4_NF, THF, rt, 4 d, and 47%. Perhydroquinoxalines **9**–**14** are racemic mixtures. For clarity, only one enantiomer of the racemic mixtures is displayed.

The synthesis of the envisaged κ receptor agonists of type **A** started with the hydroxyglutarate **4**, which was protected with a silyl protective group [25]. The resulting diester **5** was reduced with diisobutylaluminumhydride (DIBAL-H) at −78 °C in toluene [26] to afford the dialdehyde **6** in 97% yield (Scheme 1).

Dialdehyde **6** reacted with nitromethane and benzylamine [27,28] to provide the tetrasubstituted cyclohexane derivative **7** in 85% yield. This transformation can be explained by the condensation of both formyl moieties of **6** with benzylamine and the subsequent nucleophilic addition of nitromethane to the formed imines. Alternatively, a double Henry reaction of dialdehyde **6** with nitromethane could lead to nitrodiol, which is followed by the elimination of H_2_O and the subsequent conjugate addition of benzylamine at the α,β-unsaturated nitro derivative. The reaction of dialdehyde **6** with nitromethane and benzylamine led to two diastereomers **7a** and **7b** in the ratio 70:30. The NO_2_ moiety in 2-position and both BnNH moieties in 1- and 3-position adopt the thermodynamically favored equatorial orientation as reported for glutaraldehyde [22]. The diastereomers **7a** and **7b** differ in the orientation of the silyloxy moiety in 5-position. In the major diastereomer **7a** the silyloxy moiety is axially and in the minor diastereomer **7b** it is equatorially oriented. Despite four stereogenic centers, cyclohexanes **7** are achiral compounds with an intramolecular symmetry plane including both pseudochiral centers in 2- and 5-positions. **7a**, with an axially oriented silyloxy moiety, has (2*r*,5*s*)-configuration and **7b**, with an equatorially oriented silyloxy moiety, has (2*r*,5*r*)-configuration.

**Figure 2:**
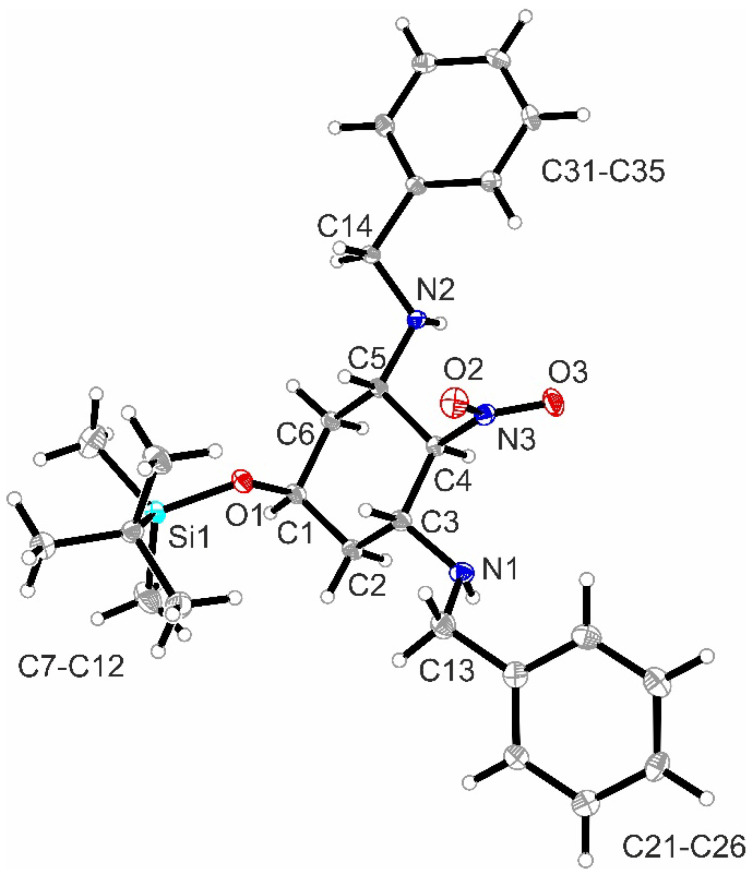
X-ray crystal structure of (2*r*,5*s*)-**7a**. Thermal ellipsoids are shown at 50% probability. CCDC Nr.: 2384408.

The major diastereomer (2*r*,5*s*)-**7a** was isolated by the recrystallization of the mixture of diastereomers **7a**/**b**. Recrystallization with Et_2_O led to crystals, which were appropriate for X-ray crystal structure analysis (Figure 2, see also Figure S1 in the Supporting Information). The structure clearly shows the intramolecular symmetry plane of (2*r*,5*s*)-**7a** with two equatorially oriented benzylamino moieties attached to the cyclohexane chair. The equatorially oriented NO_2_ moiety and the axially oriented silyloxy moiety in 2- and 5-postion lie on this symmetry plane.

**Figure 3:**
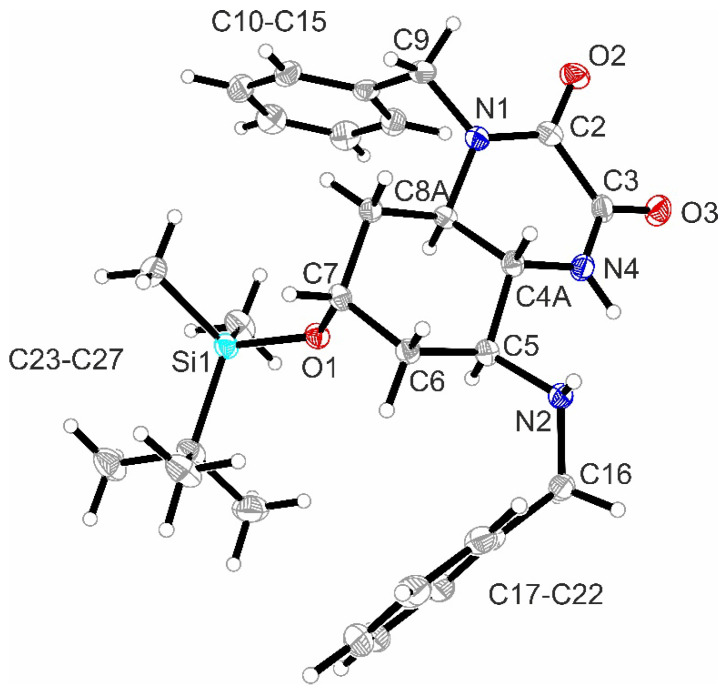
X-ray crystal structure of racemic **9**. Thermal ellipsoids are shown at 50% probability. CCDC Nr.: 2384409.

The symmetry was retained after the reduction of the NO_2_ moiety of (2*r*,5*s*)-**7a**. Several reaction conditions were explored to reduce the aliphatic NO_2_ moiety. Finally, zinc in the presence of NH_4_Cl at 60 °C [29] led to the achiral triamine (2*r*,5*s*)-**8** in 98% yield. However, the reaction of the triamine (2*r*,5*s*)-**8** with dimethyl oxalate broke the symmetry by connecting two adjacent amino moieties. The racemic quinoxalinedione **9** was obtained in 61% yield. Crystals suitable for X-ray crystal structure were obtained by the recrystallization of **9** from EtOAc and Et_2_O. The crystal structure clearly shows the *trans*-configuration of the annulated cyclohexane and piperazine rings. Furthermore, it confirms the equatorial and axial orientation of the benzylamino and silyloxy moiety, respectively (Figure 3, see also Figure S2 in the Supporting Information).

After setting up the perhydroquinoxaline framework, the final steps comprised the introduction of the pharmacophoric elements. At first, the benzyl moiety of the secondary benzylamino moiety of **9** was removed hydrogenolytically, and the resulting primary amine **10** was reacted with 1,4-diiodobutane to establish the pyrrolidino group (**11**). For the reduction of the dilactam, a mixture of LiAlH_4_ and AlCl_3_ forming AlH_3_ in situ [30] was used. The secondary amine **12** was directly converted into acetamide **13** upon the addition of 2-(3,4-dichlorophenyl)acetyl chloride. The acetamide **13** was isolated in 76% yield. In the last step, the silyl protective group was cleaved off with tetrabutylammonium fluoride to obtain the OH-substitute perhydroquinoxaline **14** in 47% yield.

In conclusion, the perhydroquinoxaline scaffold of **14** rich in sp^3^-hybridized C-atoms opens up a larger three-dimensional space in contrast to rather flat aromatic π-systems. However, the large amount of sp^3^-hybridized C-atoms led to stereochemistry problems, which required the time-consuming separation of stereoisomers. In **14**, the typical κ agonistic pharmacophoric elements, i.e., the pyrrolidine ring and the arylacetamide connected by two C-atoms [20], were embedded in an exactly defined three-dimensional orientation into the hydroxylated perhydroquinoxaline scaffold. Starting from diethyl 3-hydroxyglutarate (**4**), the hydroxylated perhydroquinoxaline **14** was stereoselectively obtained in a 10-step synthesis.

### 2.2. Biological Evaluation

The affinity toward the κ receptors of the final quinoxaline derivative **14** and its silylated precursor **13** was determined in receptor binding studies using the radioligand [^3^H]U-69,593 ([^3^H]**3**) and membrane preparations from guinea pigse [22,31]. In addition to the κ affinity, the selectivity over the related μ and δ receptors [22,31] as well as σ_1_ and σ_2_ receptors [32] was determined in receptor binding studies with radioligands. The results, together with the receptor affinity of the reference compounds, are summarized in Table 1.

Table 1 reveals considerably reduced κ receptor affinity for the hydroxylated perhydroquinoxaline **14** (K_i_ = 599 nM) compared to the lead compound **2a** with a K_i_ value of 9.4 nM [22]. Obviously, the polar OH moiety in the 7-position of **14** is less tolerated by the κ receptor. As expected, the very large *tert*-butyldimethylsilyloxy moiety in 7-position led to even lower κ receptor affinity of **13**. However, **14** displayed selectivity for the κ receptor over the related μ and δ receptors and over both σ receptor subtypes.

In addition to receptor affinity, some pharmacokinetic data were recorded for alcohol **14**. The recently established micro shake flask method [33] led to a promissing logD_7_._4_ value of 1.72 ± 0.05. The metabolic stability of **14** was determined with mouse liver microsomes and NADPH [33]. After an incubation period of 90 min, 92 ± 1% of **14** remained unchanged, indicating high metabolic stability. A high-performance affinity chromatography of **14** with a human serum albumin stationary phase [33,34] resulted in a plasma protein binding of 92.5 ± 0.03%. Although the additional OH moiety reduced the κ affinity considerably, it increased the polarity of **14** compared to **2a**. The logD_7.4_ value of 1.72 for **14** suggests good penetration of membranes and thus high bioavailability. Moreover, **14** showed high metabolic stability upon incubation with mouse liver microsomes and NADPH. The low lipophilicity and the high metabolic stability combined with 92.5% binding at human serum albumin indicate promising pharmacokinetic properties of **14.** In addition to the favorable pharmacokinetic parameters, **14** revealed high selectivity for κ receptors over the related opioid receptors (μ and δ receptors) as well as σ receptors (σ_1_ and σ_2_ receptors) compensating, at least partially, the lower κ affinity.

### 2.3. Molecular Modeling

**Figure 4:**
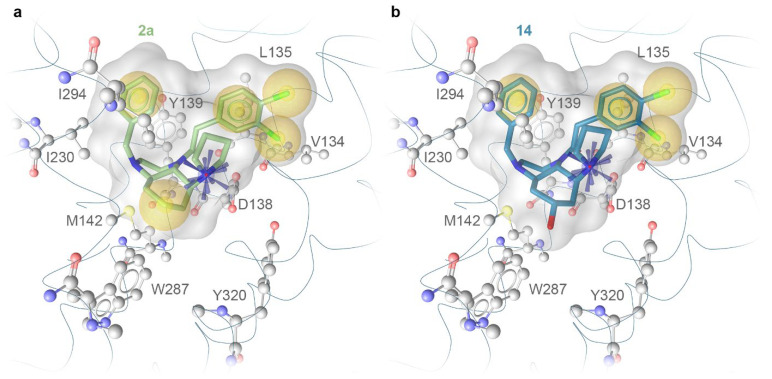
Proposed binding modes of **2a** (**a**) and **14** (**b**) with key interactions to the κ receptor derived from docking to the κ receptor (PDB ID: 6B73). The blue star illustrates the positive ionizable center, which forms a salt bridge to a conserved aspartic acid (D138). Lipophilic contacts are indicated by yellow spheres.

We applied molecular docking and 3D-pharmacophore analysis to elucidate the different binding affinities of **2a** and **14**. Both compounds were found to adopt a binding pose, which is compatible with the κ receptor binding and indicates some previously reported key interactions (Figure 4). In particular, the salt bridge formed by the protonated pyrrolidine ring and D138 is essential. Moreover, both compounds showed lipophilic contacts with two hydrophobic regions within the binding site: (i) Y139, I230, and I294 and (ii) V134 and L135. This interaction pattern is similar to the interaction pattern of MP1104, the co-crystallized agonist in the complex used for docking (PDB ID: 6B73) [16]. Interestingly, the hydroxy group of **14** points into a hydrophobic environment (M142 and W287), which could explain the lower binding affinity of **14** compared to **2a**. A potential extension of the ligand’s structure at this position is limited to small groups and might shift the ligand’s position in the binding site. Larger groups, like the TBS protecting group in the precursor **13**, are not tolerated. As expected, no reliable docking pose could be obtained for **13**.

In conclusion, receptor binding studies revealed only moderate κ receptor affinity (K_i_ = 599 nM), although the additional OH moiety was installed at the 7-position of the perhydroquinoxaline scaffold, i.e., outside the κ pharmacophore [20]. Compared to the κ agonist **2a** without OH moiety, (*K*_i_ = 9.4 nM [22]), the hydroxylated ligand **14** was less tolerated by the κ receptor. Despite only moderate κ affinity, docking studies showed the crucial κ agonist–κ receptor interactions [16]. In particular, the ionic interaction between the protonated pyrrolidine ring of **14** and D138 of the κ receptor was retained.

### 2.4. Conclusions

The typical κ agonistic pharmacophoric elements, i.e., the pyrrolidine ring and the arylacetamide connected by two C-atoms, were embedded in a hydroxylated perhydroquinoxaline scaffold resulting in **14**, which is rich in sp^3^-hybridized C-atoms. Perhydroquinoxaline **14** was stereoselectively prepared in a 10-step synthesis starting from diethyl 3-hydroxyglutarate (**4**). Receptor binding studies revealed only moderate κ receptor affinity (K_i_ = 599 nM), indicating that the additional hydroxy moiety installed in the 7-position of **14** was not well tolerated by the κ receptor. Despite only moderate κ affinity, docking studies showed the crucial κ agonist–κ receptor interactions, in particular the ionic interaction between the protonated pyrrolidine ring of **14** and D138 of the κ receptor. The related opioid receptors (μ and δ receptors) as well as σ receptors (σ_1_ and σ_2_ receptors) did not bind the perhydroquinoxaline **14,** indicating high selectivity for the κ receptor.

## 3. Materials and Methods

### 3.1. Synthetic Procedures

Diethyl 3-(*tert*-butyldimethylsilyloxy)glutarate (**5**)

Diethyl 3-hydroxyglutarate (**4**, 7.0 g, 34.3 mmol, 1.0 equiv.) and imidazole (7.0 g, 103 mmol, 3 equiv.) were dissolved in CH_2_Cl_2_ (50 mL) and the solution was cooled to 0 °C. *tert*-Butylchlorodimethylsilane (6.2 g, 41.2 mmol, 1.2 equiv.) was added in one portion and the mixture was warmed to room temperature. After stirring for 24 h, water (30 mL) was added, and the organic layer was separated. The aqueous layer was extracted with CH_2_Cl_2_ (3 × 30 mL). The combined organic layers were dried (Na_2_SO_4_) and the solvent was removed under reduced pressure. The crude product was purified by flash column chromatography (Ø 8 cm, h = 30 cm, v = 30 mL, cyclohexane:ethyl acetate = 7:1, R_f_ = 0.31). Colorless liquid, yield 10.0 g (92%). Formula: C_15_H_30_O_5_Si (318.2 g/mol). ^1^H NMR (400 MHz, CDCl_3_): δ (ppm) = 0.06 (s, 6H, Si(C*H*_3_)_2_), 0.84 (s, 9H, SiC(C*H*_3_)_3_), 1.25 (t, *J* = 7.2 Hz, 6H, 2 × OCH_2_C*H*_3_), 2.47–2.60 (m, 4H, C*H*_2_CHC*H*_2_), 4.06–4.18 (m, 4H, 2 × OC*H*_2_CH_3_), 4.54 (quint, *J* = 6.2 Hz, 1H, CH_2_C*H*CH_2_). ^13^C NMR (100 MHz, CDCl_3_): δ (ppm) = −4.8 (2C, Si(*C*H_3_)_2_), 14.3 (2C, 2 × OCH_2_*C*H_3_), 18.0 (Si*C*(CH_3_)_3_), 26.8 (3C, SiC(*C*H_3_)_3_), 42.8 (2C, *C*H_2_CH*C*H_2_), 60.6 (2C, 2 × O*C*H_2_CH_3_), 66.5 (CH_2_*C*HCH_2_), 171.2 (2C, 2 × *C*=O). HRMS (APCI): *m*/*z* = 319.1953 (calcd. 319.1935 for C_15_H_31_O_5_Si^+^ [M + H]^+^). IR (neat): ṽ [cm^−1^] = 2931 (w, C-H_aliphat._), 1735 (s, C=O), 1253, 1188 (m, C-O).

3-(*tert*-Butyldimethylsilyloxy)pentanedial (**6**)

Protected diethyl glutarate **5** (1.1 g, 3.6 mmol, 1.0 equiv.) was dissolved in dry toluene (30 mL) and the solution was cooled to −78 °C. After the addition of DIBAL-H (1.2 M in toluene, 6.7 mL, 7.9 mmol, 2.2 equiv.), the reaction mixture was stirred for 60 min at −78 °C. Then, dry acetone (2 mL) was added, and the solution was stirred for an additional 30 min at −78 °C. Afterwards, saturated potassium sodium tartrate solution (60 mL) was added at −78 °C and the mixture was warmed to room temperature overnight. The organic layer was separated, and the aqueous layer was extracted with toluene (3 × 50 mL). The combined organic layers were dried (Na_2_SO_4_), the solvent was removed in vacuo, and the residue was heated to 90 °C at 1.8 × 10^−1^ mbar in a Kugelrohr distillation apparatus. Yellow viscous oil, yield 800 mg (97%). Formula: C_11_H_22_O_3_Si (230.4 g/mol). ^1^H NMR (400 MHz, CD_2_Cl_2_): δ (ppm) = 0.09 (s, 6H, Si(C*H*_3_)_2_), 0.86 (s, 9H, SiC(C*H*_3_)_3_), 2.63–2.68 (m, 4H, C*H*_2_CHC*H*_2_), 4.72 (quint, *J* = 5.8 Hz, 1H, CH_2_C*H*CH_2_), 9.77 (t, *J* = 2.0 Hz, 2H, 2 × C*H*O). ^13^C NMR (100 MHz, CD_2_Cl_2_): δ (ppm) = −4.4 (2C, Si(*C*H_3_)_2_), 18.3 (Si*C*(CH_3_)_3_), 26.0 (3C, SiC(*C*H_3_)_3_), 51.6 (2C, *C*H_2_CH*C*H_2_), 64.1 (CH_2_*C*HCH_2_), 201.2 (2C, 2 × *C*HO). HRMS (APCI): *m*/*z* = 231.1289 (calcd. 231.1411 for C_11_H_23_O_3_Si^+^ [M + H]^+^). IR (neat): ṽ [cm^−1^] = 2854 (w, C-H_aliphat._), 2958, 2927 (w, O=C-H), 1728 (s, C=O).

(2*r*,5*s*)-N^1^,N^3^-Dibenzyl-5-(*tert*-butyldimethylsilyloxy)-2-nitrocyclohexane-1,3-diamine ((2*r*,5*s*)-**7a**) and
(2*r*,5*r*)-N^1^,N^3^-dibenzyl-5-(*tert*-butyldimethylsilyloxy)-2-nitrocyclohexane-1,3-diamine ((2*r*,5*r*)-**7b**)

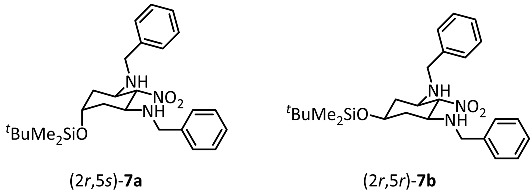


Dialdehyde **6** (1.1 g, 4.7 mmol, 1.0 equiv.) and nitromethane (447 mg, 7.1 mmol, 1.5 equiv.) were dissolved in CH_2_Cl_2_ (5 mL) and the solution was cooled to 0 °C. Afterwards, benzylamine (2.0 mL, 18.8 mmol, 4.0 equiv.) was added dropwise over 1 h. The mixture was stirred at room temperature overnight. The solvent was removed in vacuo and all the volatiles were removed in a Kugelrohr distillation apparatus (7 × 10^−2^ mbar, 65°C). Dark red resin, yield 1.4 g (85%). Ratio (2*r*,5*s*)-**7a**: (2*r*,5*r*)-**7b** = 70:30.

Formula: C_26_H_39_N_3_O_3_Si (469.7 g/mol). HPLC: Purity 84%, t_R_ = 21.8 min. ^1^H NMR (600 MHz, CD_2_Cl_2_): δ (ppm) = 0.01 (s, 4.2H, Si(C*H*_3_)_2_), 0.04 (s, 1.8H, Si(C*H*_3_)_2_), 0.84 (s, 6.3H, SiC(C*H*_3_)_3_), 0.88 (s, 2.7H, SiC(C*H*_3_)_3_), 1.20–1.29 (m, 2H, 4-*H*_ax_/6*H*_ax_), 2.08 (dt, *J* = 12.1/3.5 Hz, 1.4H, 4-*H*_eq_/6-*H*_eq_), 2.22 (dt, *J* = 12.8/4.3 Hz, 0.6H, 4-*H*_eq_/6-*H*_eq_), 3.09 (ddd, *J* = 12.2/10.4/3.9 Hz, 0.6H, 1-*H*_ax_/3-*H*_ax_), 3.56 (ddd, *J* = 11.9/10.5/4.1 Hz, 1.4H, 1-*H*_ax_/3-*H*_ax_), 3.63 (tt, *J* = 10.9/4.3 Hz, 0.3H, 5-*H*_ax_), 3.69 (d, *J* = 13.1 Hz, 1.4H, 2 × C*H*_2_Ph), 3.71 (d, *J* = 13.0 Hz, 0.6H 2 × CH_2_Ph), 3.80 (d, *J* = 12.7 Hz, 2H, 2 × C*H*_2_Ph), 4.12 (quint, *J* = 2.9 Hz, 0.7H, 5-*H*_eq_), 4.22 (t, *J* = 10.6 Hz, 0.7H, 2-*H*_ax_), 4.23 (t, *J* = 10.4 Hz, 0.3H, 2-*H*_ax_) 7.20–7.37 (m, 10H, C*H*_arom._). The signals for the N*H* protons are not seen in the spectrum. ^13^C NMR (100 MHz, CD_2_Cl_2_): δ (ppm) = −4.9 (1.4C, Si(*C*H_3_)_2_), −4.6 (0.6C, Si(*C*H_3_)_2_), 18.0 (0.7C, Si*C*(CH_3_)_3_), 18.2 (0.3C, Si*C*(CH_3_)_3_), 25.8 (2.1C, SiC(*C*H_3_)_3_), 25.9 (0.9C, SiC(*C*H_3_)_3_), 38.6 (1.4C, *C*-4/*C*-6), 40.6 (0.6C, *C*-4/*C*-6), 50.7 (0.6C, *C*H_2_Ph), 51.0 (1.4C, *C*H_2_Ph), 53.7 (1.4C, *C*-1*/C*-3), 55.6 (0.6C, *C*-1*/C*-3), 65.4 (0.7C, *C*-5), 66.9 (0.3C, *C*-5), 96.0 (0.3C, *C*-2), 97.0 (0.7C, *C*-2), 127.2 (1.4C, *C*_arom._), 127.4 (0.6C, *C*_arom._), 128.2 (4C, *C*_arom._), 128.5 (2.8C, *C*_arom._), 128.7 (1.2C, *C*_arom._), 139.8 (0.6C, *C*_quart._), 140.0 (1.4C, *C*_quart._). HRMS (APCI): *m*/*z* = 470.2813 (calcd. 470.2833 for C_26_H_40_N_3_O_3_SiH^+^ [M + H^+^]). IR (neat): ṽ [cm^−1^] = 3329 (s, N-H), 2927 (w, C-H_aliphat._), 1554 (s, NO_2_), 729, 694 (CH_arom.,monosubst._).

(2*r*,5*s*)-N^1^,N^3^-Dibenzyl-5-(*tert*-butyldimethylsilyloxy)-2-nitrocyclohexane-1,3-diamine ((2*r*,5*s*)-**7a**)

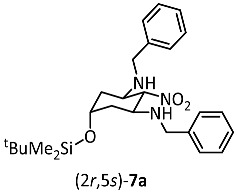


Dialdehyde **6** (2.0 g, 8.7 mmol, 1.0 equiv.) and nitromethane (800 mg, 13.1 mmol, 1.5 equiv.) were dissolved in CH_2_Cl_2_ (10 mL) and the solution was cooled to 0 °C. Afterwards, benzylamine (3.8 mL, 34.8 mmol, 4 equiv.) was added over a period of 1 h. The mixture was stirred at room temperature overnight. H_2_O (10 mL) was added, the organic layer was separated and dried (Na_2_SO_4_). The solvent was removed under reduced pressure and hexane (30 mL) was added to the residue. The hexane solution was concentrated to half and the product precipitated. Dark red crystalline solid, mp 106 °C, yield 1.2 g (29%). Formula: C_26_H_39_N_3_O_3_Si (469.7 g/mol). HPLC: Purity 97%, t_r_ = 21.9 min. ^1^H NMR (600 MHz, CDCl_3_): δ (ppm) = 0.01 (s, 6H, Si(C*H*_3_)_2_), 0.84 (s, 9H, SiC(C*H*_3_)_3_), 1.25 (td, *J* = 12.5/2.3 Hz, 2H, 4-*H*_ax_/6-*H*_ax_), 2.08 (dt, *J* = 12.1/3.4 Hz, 2H, 4-*H*_eq_/6-*H*_eq_), 3.56 (ddd, *J* = 11.9/10.5/4.1 Hz, 2H, 1-*H*_ax_/3-*H*_ax_), 3.69 (d, *J* = 13.0 Hz, 2H, C*H*_2_Ph), 3.80 (d, *J* = 13.0 Hz, 2H, C*H*_2_Ph), 4.12 (quint, *J* = 2.9 Hz, 1H, 5-*H*_eq_), 4.22 (t, *J* = 10.6 Hz, 1H, 2-*H*_ax_), 7.21–7.26 (m, 5H, C*H*_arom._), 7.27–7.34 (m, 5H, C*H*_arom._). ^13^C NMR (100 MHz, CD_2_Cl_2_): δ (ppm) = −4.9 (2C, Si(*C*H_3_)_2_), 18.0 (Si*C*(CH_3_)_3_), 25.8 (3C, SiC(*C*H_3_)_3_), 38.7 (2C, *C*-4/*C*-6), 51.0 (2C, 2 × *C*H_2_Ph), 53.7 (2C, *C*-1/*C*-3), 65.4 (*C*-5), 97.1 (*C*-2), 127.2 (2C, *C*_arom._), 128.3 (4C, *C*_arom._), 128.5 (4C, *C*_arom._), 140.1 (2C, *C*_qua rt._). HRMS (APCI): *m*/*z* = 470.2813 (calcd. 470.2833 for C_26_H_40_N_3_O_3_SiH^+^ [M + H^+^]). IR (neat): ṽ [cm^−1^] = 3329 (s, N-H), 2927 (w, C-H_aliphat._), 1554 (s, NO_2_), 729, 694 (CH_arom.,monosubst._). Crystals for X-ray crystallography were obtained by recrystallization from Et_2_O. The X-ray data are given in the Supporting Information. CCDC Nr.: 2384408.

(2*r*,5*s*)-N^1^,N^3^-Dibenzyl-5-(*tert*-butyldimethylsilyloxy)cyclohexane-1,2,3-triamine ((2*r*,5*s*)-**8**)

Nitrodiamine (2*r*,5*s*)-**7a** (1.2 g, 2.6 mmol, 1.0 equiv.) was dissolved in THF (30 mL). Saturated NH_4_Cl solution (30 mL) and Zn (1.7 g, 25.5 mmol, 10.0 equiv.) were added and the mixture was stirred for 24 h at 60 °C. Afterwards, saturated NaHCO_3_ solution (20 mL) was added, and the mixture was stirred for 1 h at room temperature. Then, the aqueous suspension was filtered through a pad of Celite^®^ using ethyl acetate (50 mL) as eluent. The organic layer was separated, and the aqueous layer was extracted with ethyl acetate (3 × 30 mL). The combined organic layers were dried (Na_2_SO_4_) and the solvent was removed under reduced pressure. Yellow solid, mp 96 °C, yield 1.1 g (98%). Formula: C_26_H_41_N_3_OSi (439.7 g/mol). HPLC: Purity 93%, t_r_ = 18.3 min. ^1^H NMR (400 MHz, DMSO-d_6_): δ (ppm) = −0.02 (s, 6H, Si(C*H*_3_)_2_), 0.82 (s, 9H, SiC(C*H*_3_)_3_), 1.20 (t, *J* = 12.4 Hz, 2H, 4-*H*_ax_/6-*H*_ax_), 1.92 (dt, *J* = 13.0/3.4 Hz, 2H, 4-*H*_eq_/6-*H*_eq_), 2.21 (t, *J* = 10.1 Hz, 1H 2-*H*_ax_), 2.59–2.65 (m, 2H, 1-*H*_ax_/3-*H*_ax_), 3.64–3.78 (m, 4H, 2 × C*H*_2_Ph), 4.06 (quint, *J* = 2.4 Hz, 1H, 5-*H*_eq_), 7.22 (t, *J* = 7.3 Hz, 2H, 2 × C*H*_arom.(*p*)_), 7.29 (t, *J* = 7.3 Hz, 4H, 4 × CH_arom.(*m*)_), 7.38 (d, *J* = 6.9 Hz, 4H, 4 × C*H*_arom.(*o*)_). The signals for the N*H* and N*H*_2_ protons are not seen in the spectrum. ^13^C NMR (100 MHz, DMSO-d_6_): δ (ppm) = −5.1 (2C, Si(*C*H_3_)_2_), 17.7 (Si*C*(CH_3_)_3_), 25.7 (3C, SiC(*C*H_3_)_3_), 37.4 (2C, *C*-4/*C*-6), 50.4 (2C, 2 × *C*H_2_Ph), 55.9 (2C, *C*-1/*C*-3), 59.3 (*C*-2), 65.8 (*C*-5), 126.7 (2C, 2 × *C*_arom.(*p*)_), 128.0 (4C, 4 × *C*_arom.(*m*)_), 128.4 (4C, 4 × *C*_arom.(*o*)_), 140.3 (2C, 2 × C_quart._). HRMS (APCI): *m*/*z* = 440.3092 (calcd. 440.3092 for C_26_H_42_N_3_OSi^+^ [M + H]^+^). IR (neat): ṽ [cm^−1^] = 3425, 3298, 3244 (w, N-H), 2951, 2927, 2885, 2854 (m, C-H_aliphat._), 1600, 1585 (w, N-H).

(4*aRS*,5*SR*,7*SR*,8*aRS*)-1-Benzyl-5-(benzylamino)-7-(*tert*-butyldimethylsilyloxy)-octahydroquinoxaline-2,3-dione (**9**)

Triamine (2*r*,5*s*)-**8** (1.0 g, 2.3 mmol, 1.0 equiv.) was dissolved in methanol (20 mL) and dimethyl oxalate (295 mg, 2.5 mmol, 1.1 equiv.) was added. The mixture was heated to reflux for 24 h and the solvent was removed under reduced pressure. The crude product was purified by flash column chromatography (Ø 6 cm, h = 25 cm, v = 20 mL, cyclohexane: CH_2_Cl_2_: methanol: NH_3(aq)_ = 492:400:100:8, R_f_ = 0.25). Pale yellow solid, mp 183 °C, yield 700 mg (61%). Formula: C_28_H_39_N_3_O_3_Si (493.7 g/mol). HPLC: Purity 99%, t_r_ = 21.0 min. ^1^H NMR (600 MHz, CDCl_3_): δ (ppm) = −0.23 (s, 3H, Si(C*H*_3_)_2_), −0.06 (s, 3H, Si(C*H*_3_)_2_), 0.69 (s, 9H, SiC(C*H*_3_)_3_), 1.17–1.23 (m, 1H, 6-*H*_ax_), 1.47 (td, *J* = 12.5/2.3 Hz, 1H, 8-*H*_ax_), 2.10 (dtd, *J* = 12.6/3.7/1.9 Hz, 1H, 8-*H*_eq_), 2.19 (dtd, *J* = 13.1/3.6/1.9 Hz, 1H, 6-*H*_eq_), 2.88 (td, *J* = 11.1/3.7 Hz, 1H, 5-*H*_ax_), 3.14 (t, *J* = 10.0 Hz, 1H, 4a-*H*_ax_), 3.66 (d, *J* = 12.8 Hz, 1H, NH-C*H*_2_Ph), 3.84–3.93 (m, 2H, 8a-*H*_ax_/NH-C*H*_2_Ph), 4.15 (quint, *J* = 2.9 Hz, 1H, 7-*H*_eq_), 4.18 (d, *J* = 15.6 Hz, 1H, N-C*H*_2_Ph), 5.39 (d, *J* = 15.6 Hz, 1H, N-C*H*_2_Ph), 7.16 (d, *J* = 7.2 Hz, 2H, C*H*_arom_.), 7.19–7.24 (m, 1H, C*H*_arom._), 7.26–7.35 (m, 7H, C*H*_arom._). The signals for the N*H* protons are not seen in the spectrum. ^13^C NMR (100 MHz, CDCl_3_): δ (ppm) = −5.3 (Si(*C*H_3_)_2_), −5.1 (Si(*C*H_3_)_2_), 17.8 (Si*C*(CH_3_)_3_), 25.6 (3C, SiC(*C*H_3_)_3_), 35.2 (*C*-8), 37.7 (*C*-6), 45.9 (N-*C*H_2_Ph), 50.6 (NH-*C*H_2_Ph), 52.7 (*C*-5), 53.1 (*C*-8a), 58.2 (*C*-4a), 65.4 (*C*-7), 127.1 (2C, *C*_arom._), 127.7 (2C, *C*_arom._), 128.4 (2C, *C*_arom._), 128.8 (2C, *C*_arom._), 129.0 (2C, *C*_arom._), 136.1 (*C*_quart._), 139.4 (*C*_quart._), 157.5 (*C*-3), 159.7 (*C*-2). HRMS (APCI): *m*/*z* = 494.2837 (calcd. 494.2833 for C_28_H_40_N_3_O_3_Si^+^ [M + H]^+^). IR (neat): ṽ [cm^−1^] = 3340, 3302 (m, N-H), 2954, 2924, 2893, 2854 (m, C-H_aliphat._), 1701, 1667 (s, C=O), 740 (m, C-H_arom, monosubst._). Crystals for X-ray crystallography were obtained by recrystallization from ethyl acetate and Et_2_O. The X-ray data are given in the Supporting Information. CCDC Nr.: 2384409.

(4*aRS*,5*SR*,7*SR*,8*aRS*)-5-Amino-1-benzyl-7-(*tert*-butyldimethylsilyloxy)-octahydro-quinoxaline-2,3-dione (**10**)

Quinoxalinedione **9** (300 mg, 0.6 mmol, 1.0 equiv.) was dissolved in methanol (20 mL). Under nitrogen flow, Pd/C (300 mg) was added, and the mixture was shaken at room temperature for 48 h under H_2_ atmosphere (5 bar). The mixture was filtered through a pad of Celite^®^ using methanol (30 mL) as the eluent. The solvent was removed under reduced pressure. The crude product was purified by flash column chromatography (Ø 2 cm, h = 25 cm, v = 10 mL, cyclohexane: CH_2_Cl_2_: methanol: NH_3(aq)_ = 492:400:100:8, R_f_ = 0.12). Pale yellow solid, mp 97 °C, yield 120 mg (49%). Formula: C_21_H_33_N_3_O_3_Si (403.6 g/mol). HPLC: Purity 93%, t_r_ = 18.3 min. ^1^H NMR (600 MHz, CDCl_3_): δ (ppm) = −0.23 (s, 3H, Si(C*H*_3_)_2_), −0.07 (s, 3H, Si(C*H*_3_)_2_), 0.69 (s, 9H, SiC(C*H*_3_), 1.37 (t, *J* = 12.5 Hz, 1H, 6-*H*_ax_), 1.48 (td, *J* = 12.4/2.3 Hz, 1H, 8-*H*_ax_), 1.94 (d, *J* = 13.4 Hz, 1H, 6-*H*_eq_), 2.09 (dtd, *J* = 12.7/3.7/2.0 Hz, 1H, 8-*H*_eq_), 2.96–3.05 (m, 1H, 5-*H*_ax_), 3.15 (t, *J* = 10.6 Hz, 1H, 4a-*H*_ax_), 3.91 (ddd, *J* = 12.2/10.8/3.8 Hz, 1H, 8a-*H*_ax_), 4.09 (quint, *J* = 2.9 Hz, 1H, 7-*H*_eq_), 4.21 (d, *J* = 15.7 Hz, 1H, N-C*H*_2_Ph), 5.38 (d, *J* = 15.6 Hz, 1H, N-C*H*_2_Ph), 7.17 (d, *J* = 7.2 Hz, 2H, C*H*_arom._), 7.22 (t, *J* = 7.4 Hz, 1H, C*H*_arom._), 7.29 (t, *J* = 7.4 Hz, 2H, C*H*_arom._). The signals for the N*H* and N*H*_2_ protons are not seen in the spectrum. ^13^C NMR (100 MHz, CDCl_3_): δ (ppm) = −5.3 (Si(*C*H_3_)_2_), −5.1 (Si(*C*H_3_)_2_), 17.8 (Si*C*(CH_3_)_3_), 25.7 (3C, SiC(*C*H_3_)_3_), 35.3 (*C*-8), 43.0 (*C*-6), 45.9 (N-*C*H_2_Ph), 47.9 (*C*-5), 53.1 (*C*-8a), 59.5 (*C*-4a), 65.6 (*C*-7), 127.1 (2C, *C*_arom._), 127.7 (*C*_arom._), 129.0 (2C, *C*_arom._), 136.1 (*C*_quart._), 157.8 (*C*-3), 159.7 (*C*-2). HRMS (APCI): *m*/*z* = 404.2366 (calcd. 404.2364 for C_21_H_34_N_3_O_3_Si^+^ [M + H]^+^). IR (neat): ṽ [cm^−1^] = 3263 (w, N-H), 2951, 2927, 2885, 2854 (m, C-H_aliphat._), 1701, 1670 (s, C=O), 1624 (w, C-N), 775 (m, C-H_arom., monosubst._).

(4*aRS*,5*SR*,7*SR*,8*aRS*)-1-Benzyl-7-(*tert*-butyldimethylsilyloxy)-5-(pyrrolidin-1-yl)-octahydroquinoxaline-2,3-dione (**11**)

The primary amine **10** (134 mg, 0.33 mmol, 1.0 equiv.) was dissolved in THF (10 mL) and Na_2_CO_3_ (249 mg, 2.31 mmol, 7.0 equiv.) was added. 1,4-Diiodobutane (170 µL, 1.32 mmol, 4.0 equiv.) was added to the suspension and the reaction mixture was heated to reflux for 4 d. Na_2_CO_3_ was filtered off and the solvent was removed under reduced pressure. The crude product was purified by column chromatography (Ø 2 cm, h = 20 cm, v = 5 mL, cyclohexane:CH_2_Cl_2_:methanol:NH_3(aq)_ = 492:400:100:8, R_f_ = 0.33). Pale yellow solid, mp 180 °C, yield 112 mg (73%). Formula: C_25_H_39_N_3_O_3_Si (457.7 g/mol). HPLC: Purity 82%, t_r_ = 19.8 min. ^1^H NMR (600 MHz, CD_2_Cl_2_): δ (ppm) = −0.17 (s, 3H, Si(C*H*_3_)_2_), −0.03 (s, 3H, Si(C*H*_3_)_2_), 0.73 (s, 9H, SiC(C*H*_3_)_3_), 1.39–1.47 (m, 2H, 6-*H*_ax_/8-*H*_ax_), 1.69–1.81 (m, 5H, 8-*H*_eq_/N(CH_2_C*H*_2_)_2_), 2.08 (dtd, *J* = 12.8/3.8/1.9 Hz, 1H, 6-*H*_eq_), 2.49–2.61 (m, 4H, N(C*H*_2_CH_2_)_2_), 3.13–3.20 (m, 1H, 5-*H*_ax_), 3.29 (t, *J* = 10.8 Hz, 1H, 4a-*H*_ax_), 3.94 (ddd, *J* = 12.1/10.6/3.9 Hz, 1H, 8a-*H*_ax_), 4.24 (quint, *J* = 3.0 Hz, 1H, 7-*H*_eq_), 4.29 (d, *J* = 15.6 Hz, 1H, N-C*H*_2_Ph), 5.21 (d, *J* = 15.6 Hz, 1H, N-C*H*_2_Ph), 7.19 (d, *J* = 7.1 Hz, 2H, C*H*_arom.(*o*)_), 7.25 (t, *J* = 7.4 Hz, 1H, C*H*_arom.(*p*)_), 7.31 (t, *J* = 7.5 Hz, 2H, C*H*_arom.(*m*)_). The signal for the N*H* proton is not seen in the spectrum. ^13^C NMR (150 MHz, CD_2_Cl_2_): δ (ppm) = −5.1 (1C, Si(*C*H_2_)_2_), −4.9 (1C, Si(*C*H_2_)_2_), 18.2 (Si*C*(CH_3_)_3_), 24.1 (2C, 2 × NCH_2_*C*H_2_), 25.9 (3C, SiC(*C*H_3_)_3_), 28.3 (*C*-8), 35.8 (*C*-6), 46.2 (N-*C*H_2_Ph), 47.4 (2C, 2 × N*C*H_2_CH_2_), 54.4 (*C*-8a), 54.7 (*C*-5), 56.2 (*C*-4a), 66.2 (*C*-7), 127.4 (2C, 2 × *C*_arom.(*o*)_), 127.9 (*C*_arom.(*p*)_), 129.4 (2C, 2 × *C*_arom.(*m*)_), 137.1 (*C*_quart._), 157.6 (*C*-3), 160.3 (*C*-2). HRMS (APCI): *m*/*z* = 458.2831 (calcd. 458.2833 for C_25_H_40_N_3_O_3_Si^+^ [M + H]^+^). IR (neat): ṽ [cm^−1^] = 3340 (m, N-H), 2951, 2924, 2854, 2804 (m, C-H_aliphat._), 1708, 1666 (s, C=O), 1357 (m, C-N).

1-[(4*aRS*,6*SR*,8*SR*,8*aRS*)-4-Benzyl-6-(*tert*-butyldimethylsilyloxy)-8-(pyrrolidin-1-yl)-decahydroquinoxalin-1-yl]-2-(3,4-dichlorophenyl)ethan-1-one (**13**)

AlCl_3_ (64 mg, 0.48 mmol, 2.0 equiv.) was dissolved in dry THF (3 mL) and the mixture was cooled to 0 °C. LiAlH_4_ (1 M in THF, 1.44 mL, 1.44 mmol, 6.0 equiv.) was added dropwise. The mixture was warmed to room temperature and stirred for 20 min. Afterwards, the solution was cooled to 0 °C and a solution of the diamide **11** (112 mg, 0.24 mmol, 1.0 equiv.) in THF (3 mL) was added. The mixture was stirred for 45 min at 0 °C and another 20 min at room temperature. Then, NaOH solution (1 M, 2 mL) was added, and the slurry was extracted with CH_2_Cl_2_ (5 × 5 mL). The organic layer was dried (Na_2_SO_4_), and the solvent was removed under reduced pressure. The residue (compound **12**) was dissolved in CH_2_Cl_2_ (5 mL) and 2-(3,4-dichlorophenyl)acetyl chloride (65 mg, 0.29 mmol, 1.2 equiv.) was added. The mixture was stirred for 18 h at room temperature. NaOH solution (1 M, 4 mL) was added and after stirring for 2 h at room temperature, the organic layer was separated, and the aqueous layer was extracted with CH_2_Cl_2_ (3 × 5 mL). The combined organic layers were washed with brine (10 mL) and dried (Na_2_SO_4_). The solvent was removed in vacuo. The residue was suspended in pentane (15 mL). After filtration, the solvent was removed in vacuo. Pale yellow solid, mp 73–75 °C, yield 112 mg (75%). Formula: C_33_H_47_Cl_2_N_3_O_2_Si (616.7 g/mol). HPLC: Purity 95%, t_r_ = 22.6 min. ^1^H NMR (400 MHz, CD_3_OD_,_ 60 °C): δ (ppm) = 0.12 (s, 6H, Si(C*H*_3_)_2_), 0.95 (s, 9H, SiC(C*H*_3_)_3_), 1.49 (ddd, *J* = 13.7/11.9/2.5 Hz, 1H, 5-*H*_ax_), 1.62 (ddd, *J* = 14.4/12.5/2.4 Hz, 1H, 7-*H*_ax_), 1.74–1.84 (m, 4H, N(CH_2_C*H*_2_)_2_), 2.00–2.06 (m, 1H, 7-*H*_eq_), 2.18–2.25 (m, 1H, 5-*H*_eq_), 2.25–2.33 (m, 1H, 3-H_eq_), 2.73 (dt, *J* = 11.9/6.0 Hz, 1H, 3-*H*_ax_), 2.81–2.95 (m, 4H, N(C*H*_2_CH_2_)_2_), 3.07 (ddd, *J* = 11.3/9.8/3.8 Hz, 1H, 4a-*H*_ax_), 3.27–3.34 (m, 1H, 2-*H*_ax_), 3.40 (d, *J* = 13.6 Hz, 1H, N-C*H*_2_Ph), 3.49–3.66 (m, 3H, 2-*H*_eq_/C*H*_2_C=O), 3.74 (d, *J* = 13.6 Hz, 1H, N-C*H*_2_Ph), 3.74–3.83 (m, 1H, 8-*H*_ax_), 4.03–4.23 (m, 1H, 8a-*H*_ax_), 4.35 (quint, *J* = 3.0 Hz, 1H, 6-*H*_eq_), 7.14–7.30 (m, 6H, C*H*_arom._), 7.38 (d, *J* = 8.3 Hz, 1H, C*H*_arom._), 7.47 (d, *J* = 2.1 Hz, 1H, C*H*_arom._). ^13^C NMR (100 MHz, CD_3_OD, 60 °C): δ (ppm) = −4.7 (2C, Si(*C*H_3_)_2_), 18.9 (Si*C*(CH_3_)_3_), 24.7 (2C, N(CH_2_*C*H_2_)_2_), 26.4 (3C, SiC(*C*H_3_)_3_), 38.7 (*C*-5), 50.5 (2C, N(*C*H_2_CH_2_)_2_), 53.3 (*C*-3), 57.4 (N-*C*H_2_Ph), 57.8 (*C*-4a), 67.5 (*C*-6), 128.1 (*C*_arom._), 129.3 (2C, *C*_arom._), 129.9 (2C, *C*_arom._), 130.2 (*C*_arom._), 131.5 (*C*_arom._), 131.8 (*C*_quart._), 132.2 (*C*_arom._), 133.3 (*C*_quart._), 137.5 (*C*_quart._), 139.7 (*C*_quart._), 174.1 (*C*=O). The signals for *C*-2, *C*-7, *C*-8, *C*-8a, and *C*H_2_C=O are not seen in the spectrum. HRMS (APCI): *m*/*z* = 616.2885 (calcd. 616.2887 for C_33_H_48_^35^Cl_2_N_3_O_2_Si^+^ [M + H]^+^). IR (neat): ṽ [cm^−1^] = 2954, 2924, 2854 (s, C-H_aliphat._), 1647 (s, C=O), 1458 (s, CH_2_), 1388 (m, C-N), 1357 (m, C-N), 833 (m, C-Cl), 775 (C-H_arom., disubst._)

1-[(4*aRS*,6*SR*,8*SR*,8*aRS*)-4-Benzyl-6-hydroxy-8-(pyrrolidin-1-yl)decahydro-quinoxalin-1-yl]-2-(3,4-dichlorophenyl)ethan-1-one (**14**)

Silyl ether **13** (67 mg, 0.11 mmol, 1.0 equiv.) was dissolved in THF (3 mL) and TBAF × 3H_2_O (69 mg, 0.22 mmol, 2.0 equiv.) was added in one portion. The mixture was stirred for 4 d at room temperature. H_2_O (5 mL) was added, the aqueous layer was extracted with CH_2_Cl_2_ (5 × 10 mL), and the combined organic layers were dried (Na_2_SO_4_). The solvent was removed in vacuo. The crude product was purified by flash column chromatography (Ø 2 cm, h = 20 cm, v = 5 mL, cyclohexane: CH_2_Cl_2_: methanol: NH_3(aq)_ = 371:100:25:4, R_f_ = 0.17). Colorless solid, mp 74 °C, yield 26 mg (47%). Formula: C_27_H_33_Cl_2_N_3_O_2_ (502.5 g/mol). HPLC: Purity 96%, t_r_ = 15.9 min. ^1^H NMR (400 MHz, CD_3_OD, 60 °C): δ (ppm) = 1.50 (td, *J* = 12.6/3.0 Hz, 1H, 5-*H*_ax_), 1.55–1.64 (m, 1H, 7-*H*_ax_), 1.64–1.73 (m, 4H, N(CH_2_C*H*_2_)_2_), 2.08 (d, *J* = 13.6 Hz, 1H, 7-*H*_eq_), 2.20 (d, *J* = 12.6 Hz, 1H, 5-*H*_eq_), 2.36 (dt, *J* = 10.5/4.9 Hz, 1H, 3-*H*_eq_), 2.56–2.80 (m, 5H, 3-*H*_ax_,/N(C*H*_2_CH_2_)_2_), 3.07 (ddd, *J* = 12.8/9.6/3.7 Hz, 1H, 4a-*H*_ax_), 3.37 (d, *J* = 13.4 Hz, 1H, N-C*H*_2_Ph), 3.40–3.51 (m, 1H, 2-*H*_ax_), 3.53–3.69 (m, 3H, 2-*H*_eq_/C*H*_2_C=O), 3.73 (d, *J* = 13.4 Hz, 1H, N-C*H*_2_Ph), 3.76–3.86 (m, 2H, 8a-*H*_ax_,/8-*H*_ax_), 4.24 (quint, *J* = 3.1 Hz, 1H, 6-*H*_eq_), 7.11–7.33 (m,6H, C*H*_arom._), 7.40 (d, *J* = 8.3 Hz, 1H, C*H*_arom._), 7.48 (d, *J* = 2.0 Hz, 1H, C*H*_arom_.). The signal for the O*H* proton is not seen in the spectrum. ^13^C NMR (100 MHz, CD_3_OD, 60 °C): δ (ppm) = 24.7 (2C, N(CH_2_*C*H_2_)_2_), 38.8 (*C*-5), 48.9 (2C, N(*C*H_2_CH_2_)_2_), 53.1 (*C*-3), 57.1 (N-*C*H_2_Ph), 57.5 (*C*-4a), 66.4 (*C*-6), 128.1 (*C*_arom_.), 129.3 (2C, *C*_arom._), 130.0 (2C, *C*_arom._), 130.2 (*C*_arom._), 131.5 (*C*_arom._), 131.6 (*C*_quart._), 132.1 (*C*_arom._), 133.3 (*C*_quart._), 137.7 (*C*_quart._), 140.0 (*C*_quart._), 173.4 (*C*=O). The signals for *C*-2, *C*-7, *C*-8, *C*-8a, and *C*H_2_C=O are not seen in the spectrum. HRMS (ESI): *m*/*z* = 502.2013 (calcd. 502.2023 for C_27_H_34_^35^Cl_2_N_3_O_2_^+^ [M + H]^+^). IR (neat): ṽ [cm^−1^] = 3387 (m, O-H), 2924, 2873, 2854, 2800 (m, C-H_aliphat._), 1627 (s, C=O), 1134 (C-O).

### 3.2. Receptor Binding Studies

Materials, preparation of membrane homogenates from various tissues, protein determination, and the general and specific procedures for binding assays are detailed in the Supplementary Materials (Tables S1–S11).

### 3.3. Determination of κ Receptor Affinity (Guinea Pig Brain) [22,31]

The assay was performed with the radioligand [^3^H]U-69,593 (55 Ci/mmol, Amersham, Little Chalfont, UK). The thawed guinea pig brain membrane preparation (about 100 μg of the protein) was incubated with various concentrations of test compounds, 1 nM [^3^H]U-69,593, and TRIS-MgCl_2_-buffer (50 mM, 8 mM MgCl_2_, pH 7.4) at 37 °C. The non-specific binding was determined with 10 μM unlabeled U-69,593. The K_d_-value of U-69,593 is 0.69 nM.

### 3.4. Molecular Modeling

Molecular docking was performed with AutoDock 4.2 as implemented in the LigandScout framework by applying default settings [35,36]. Structural models were preprocessed with MOE 2022.02 (Molecular Operating Environment). We used the crystal structure of the MP1104-bound κ receptor obtained from the RCSB database [16] and selected chain A for all the docking attempts with an interaction cutoff threshold of 10 Å. Due to the structural similarity with known ligands including U-55,0488 and U-69,593, we considered the pyrrolidine moiety to be protonated in the bound form. The resulting docking poses were evaluated by taking receptor–ligand interactions into account. Three-dimensional pharmacophore models were built with LigandScout 4.4 by using the default preferences for the geometric feature definitions [35,37]. The selected docking poses were energy-minimized before calculating pharmacophores, but only minor conformational changes have been observed. The shown interactions are based on geometric feature definitions (based on distance ranges and angles) that have been previously described by Wolber and Langer [31].

## Figures and Tables

**Table 1:** Affinity of novel perhydroquinoxalines **13** and **14** towards κ receptors and related receptors.

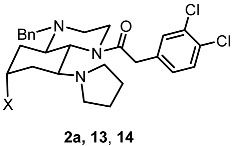						
Compd.	X	*K*_i_ ± SEM [nM] ^(a,b)^				
		κ Receptor	μ Receptor	δ Receptor	σ_1_ Receptor	σ_2_ Receptor
		[^3^H]U-69,593	[^3^H]DAMGO	[^3^H]DPDPE	[^3^H]-(+)-pentazocine	[^3^H]DTG
**2a** [22]	H	9.4 ± 1.6	3900	39%	1390	4390
**13**	OTBS	0%	--	--	487	7%
**14**	OH	599 ± 217	0%	11%	7900	0%
U-50,488		0.34 ± 0.07				
U-69,593		0.88 ± 0.10	-	-	-	-
naloxone		6.9 ± 0.50	2.3 ± 1.1	103	-	-
morphine		35 ± 6	5.2 ± 1.6	-	-	-
SNC80		-	-	1.2 ± 0.5	-	-
(+)-pentazocine		-	-	-	5.4 ± 0.5	-
haloperidol		-	-	-	6.6 ± 0.9	78 ± 2.3

^(a)^ values in % reflect the inhibition of the radioligand binding at a test compound concentration of 1 μM. K_i_ values without SEM values represent the result of one experiment and K_i_ values with SEM values represent the mean of three independent experiments (n = 3). ^(b)^ guinea pig brain membrane preparations were used in the κ, μ, and σ_1_ assay. In the δ assay, rat brain and in the σ_2_ assay, rat liver membrane preparations were used.

## Data Availability

Data is contained within the article and Supplementary Materials.

## Supplementary Materials

The following supporting information can be downloaded at https://www.mdpi.com/article/10.3390/ijms26030998/s1. Refs. [38,39,40,41,42] are cited in Supplementary Materials file.

## Abbreviations

DIBAL-H: diisobutylaluminumhydride; DOP: δ receptor; EAE: experimental autoimmune encephalomyelitis; KOP: κ receptor; MOP: μ receptor; NOP: nociception opioid receptor; TBS: *tert*-butyldimethylsilyl.
