# Supplementary material for: Stereoselective Synthesis and Biological Evaluation of Perhydroquinoxaline-Based κ Receptor Agonists
Source: Int J Mol Sci. 2025 Jan 24;26(3):998. doi: 10.3390/ijms26030998 (PMC11817610; doi:10.3390/ijms26030998)
Supplement: Supplementary file 1 [file ijms-26-00998-s001.zip › ijms-3396230-supplementary.pdf]

Supporting information

Stereoselective synthesis and biological evaluation of perhydroquinoxaline-based κ receptor agonists

Jonathan Hoffmann,[a] Dirk Schepmann,[a] Constantin Daniliuc,[b] Marcel Bermudez,[a] Bernhard Wünsch[a,c] *

[a] Universität Münster, Institut für Pharmazeutische und Medizinische Chemie, Corrensstraße 48, D-48149 Münster, Germany.
Tel.: +49-251-8333311; Fax: +49-251-8332144; E-mail: wuensch@uni-muenster.de

[b] Universität Münster, Organisch-Chemisches Institut, Corrensstraße 40, D-48149 Münster, Germany.

[c] Universität Münster, GRK 2515, Chemical biology of ion channels (Chembion), Correnstr. 48, D-48149 Münster, Germany.

Content page

1. Chemistry, general methods S2
2. HPLC method for the determination of the purity S3
3. Synthesis of 2-(3,4-dichlorophenyl)acetyl chloride S3
4. X-ray crystal structure analyses S4
5. Receptor binding studies S25
6. Binding curves of **14** and **13** at κ and related receptors S29
7. ^{1}H and ^{13}C NMR spectra S33

1. Chemistry, General Methods

Oxygen and moisture sensitive reactions were carried out under nitrogen, dried with silica gel with moisture indicator (orange gel, VWR, Darmstadt, Germany) and in dry glassware (Schlenk flask or Schlenk tube). Temperature was controlled with dry ice/acetone (-78 °C), ice/water (0 °C), Cryostat (Julabo TC100E-F, Seelbach, Germany), magnetic stirrer MR 3001 K (Heidolph, Schwalbach, Germany) or RCT CL (IKA, Staufen, Germany), together with temperature controller EKT HeiCon (Heidolph) or VT-5 (VWR) and PEG or silicone bath. All solvents were of analytical or technical grade quality. Demineralized water was used. CH_2Cl_2 was distilled from CaH_2; THF was distilled from sodium/benzophenone; MeOH was distilled from magnesium methanolate. Thin layer chromatography (tlc): tlc silica gel 60 F_{254} on aluminum sheets (VWR). Flash chromatography (fc): Silica gel 60, 40–63 µm (VWR); parentheses include: diameter of the column (∅), length of the stationary phase (h), fraction size (v) and eluent. Automated flash chromatography: Isolera™ Spektra One (Biotage®); parentheses include: cartridge size, eluent, fraction size was always 20 mL. Melting point: Melting point system MP50 (Mettler Toledo, Gießen, Germany), open capillary, uncorrected. MS: MicroTOFQII mass spectrometer (Bruker Daltonics, Bremen, Germany); deviations of the found exact masses from the calculated exact masses were 5 ppm or less; the data were analyzed with DataAnalysis® (Bruker Daltonics). NMR: NMR spectra were recorded in deuterated solvents on Agilent DD2 400 MHz and 600 MHz spectrometers (Agilent, Santa Clara CA, USA); chemical shifts (δ) are reported in parts per million (ppm) against the reference substance tetramethylsilane and calculated using the solvent residual peak of the undeuterated solvent; coupling constants are given with 0.5 Hz resolution; assignment of ^{1}H and ^{13}C NMR signals was supported by 2-D NMR techniques where necessary. IR: FT/IR IR Affinity®-1 spectrometer (Shimadzu, Düsseldorf, Germany) using ATR technique.

2. HPLC method for the determination of the purity

Pump: L-7100, degasser: L-7614, autosampler: L-7200, UV detector: L-7400, interface: D-7000, data transfer: D-line, data acquisition: HSM-Software (all from Merck Hitachi, Darmstadt, Germany); column: LiChrospher® 60 RP-select B (5 µm), LiChroCART® 250-4 mm cartridge; flow rate: 1.0 mL/min; injection volume: 5.0 µL; detection at λ = 210 nm; solvents: A: demineralized water with 0.05 % (V/V) trifluoroacetic acid, B: CH_3CN with 0.05 % (V/V) trifluoroacetic acid; gradient elution (% A): 0 – 4 min: 90 %; 4 – 29 min: gradient from 90 % to 0 %; 29 – 31 min: 0 %; 31 – 31.5 min: gradient from 0 % to 90 %; 31.5 – 40 min: 90 %. Unless otherwise mentioned, the purity of all test compounds is greater than 95 %.

3. Synthesis of 2-(3,4-dichlorophenyl)acetyl chloride

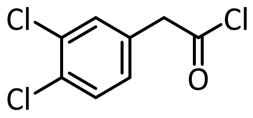

50

2-(3,4-Dichlorophenyl)acetic acid (1.0 g, 4.88 mmol, 1.0 equiv.) was treated with $SOCl_2$ (0.7 mL, 9.76 mmol, 2.0 equiv.). The mixture was heated to 60 °C for 3 h. The excess of $SOCl_2$ was removed in vacuum. Pale yellow liquid, yield 1.09 g (100 %).

^{1}H NMR (400 MHz, $CDCl_3$): δ (ppm) = 4.11 (s, 2H, CH_2C=O), 7.11 (dd, *J* = 8.2/2.1 Hz, 1H, C$H_{arom.}$), 7.38 (d, *J* = 2.1 Hz, 1H, C$H_{arom.}$), 7.45 (d, *J* = 8.2 Hz, 1H, C$H_{arom.}$).

4. X-ray crystal structure analyses

4.1. X-Ray diffraction

Data sets for compounds (2*r*,5*s*)-**7a** and racemic-**9** were collected with a Bruker D8 Venture CMOS diffractometer. Programs used: data collection: APEX3 V2016.1-0 (Bruker AXS Inc., **2016**); cell refinement: SAINT V8.37A (Bruker AXS Inc., **2015**); data reduction: SAINT V8.37A (Bruker AXS Inc., **2015**); absorption correction, SADABS V2014/7 (Bruker AXS Inc., **2014**); structure solution SHELXT-2015 (Sheldrick, **2015**); structure refinement SHELXL-2015 (Sheldrick, **2015**). *R*-values are given for observed reflections, and wR^2 values are given for all reflections.

Exceptions and special features: For compound (2*r*,5*s*)-**7a** one silyloxy moiety was found disordered over two positions in the asymmetric unit. Several restraints (SADI, SAME, ISOR and SIMU) were used in order to improve refinement stability.

4.2. X-ray crystal structure analysis of (2*r*,5*s*)-*N*^1^,*N*^3^-dibenzyl-5-(*tert*-butyldimethylsilyloxy)-2-nitrocyclohexane-1,3-diamine ((2*r*,5*s*)-7a), (dan8662)

A colorless prism-like specimen of $C_{26}H_{39}N_3O_3Si$, approximate dimensions 0.136 mm x 0.174 mm x 0.257 mm, was used for the X-ray crystallographic analysis. The X-ray intensity data were measured. The integration of the data using a triclinic unit cell yielded a total of 48603 reflections to a maximum θ angle of 66.87° (0.84 Å resolution), of which 18435 were independent (average redundancy 2.636, completeness = 96.2%, R_{int} = 5.69%, R_{sig} = 6.58%) and 15032 (81.54%) were greater than $2\sigma(F^2)$. The final cell constants of a = 7.5603(3) Å, b = 26.7393(9) Å, c = 29.7893(10) Å, α = 114.235(1)°, β = 95.141(2)°, γ = 95.803(2)°, volume = 5405.9(3) Å^3, are based upon the refinement of the XYZ-centroids of reflections above 20 σ(I). Data were corrected for absorption effects using the multi-scan method (SADABS). The calculated minimum and maximum transmission coefficients (based on crystal size) are 0.7830 and 0.8760. The structure was solved and refined using the Bruker SHELXTL Software Package, using the space group *P*-1, with Z = 8 for the formula unit, $C_{26}H_{39}N_3O_3Si$. The final anisotropic full-matrix least-squares refinement on F^2 with 1310 variables converged at R1 = 8.63%, for the observed data and wR2 = 20.71% for all data. The goodness-of-fit was 1.138. The largest peak in the final difference electron density synthesis was 0.791 e^-/Å^3 and the largest hole was -0.482 e^-/Å^3 with an RMS deviation of 0.079 e^-/Å^3. On the basis of the final model, the calculated density was 1.154 g/cm^3 and F(000), 2032 e^-. The hydrogen atoms located

at N1A and N3A were refined freely, but with N-H distance restraints (DFIX). CCDC Nr.: 2384408.

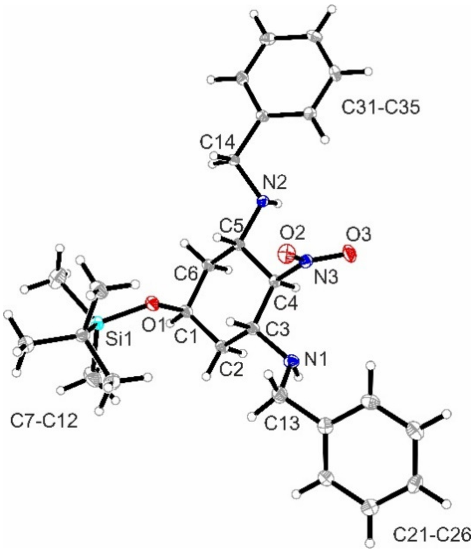

Figure S1: Crystal structure of compound (2*r*,5*s*)-**7a**. Thermal ellipsoids are shown at 50% probability.

Table S1: Sample and crystal data for (2*r*,5*s*)-**7a**

Identification code	dan8662	
Chemical formula	$C_{26}H_{39}N_3O_3Si$	
Formula weight	469.69 g/mol	
Temperature	100(2) K	
Wavelength	1.54178 Å	
Crystal size	0.136 x 0.174 x 0.257 mm	
Crystal habit	colorless prism	
Crystal system	Triclinic	
Space group	P -1	
Unit cell dimensions	a = 7.5603(3) Å	α = 114.2350(10)°
	b = 26.7393(9) Å	β = 95.141(2)°
	c = 29.7893(10) Å	γ = 95.803(2)°
Volume	5405.9(3) $Å^3$	
Z	8	
Density (calculated)	1.154 g/cm^3	
Absorption coefficient	1.001 mm^{-1}	
F(000)	2032	

Table S2: Data collection and structure refinement for (2*r*,5*s*)-**7a**

Theta range for data collection	2.94 to 66.87°	
Index ranges	-8<=h<=8, -31<=k<=31, -35<=l<=35	
Reflections collected	48603	
Independent reflections	18435 [R(int) = 0.0569]	
Absorption correction	multi-scan	
Max. and min. transmission	0.8760 and 0.7830	
Structure solution technique	direct methods	
Structure solution program	SHELXL-2014/7 (Sheldrick, 2014)	
Refinement method	Full-matrix least-squares on F^2	
Refinement program	SHELXL-2014/7 (Sheldrick, 2014)	
Function minimized	$\Sigma w(F_o^2 - F_c^2)^2$	
Data / restraints / parameters	18435 / 153 / 1310	
Goodness-of-fit on F^2	1.138	
Δ/σ_{max}	0.001	
Final R indices	15032 data; I>2σ(I)	R1 = 0.0863, wR2 = 0.2000
	all data	R1 = 0.1019, wR2 = 0.2071
Weighting scheme	$w=1/[\sigma^2(F_o^2)+25.2474P]$ where $P=(F_o^2+2F_c^2)/3$	
Largest diff. peak and hole	0.791 and -0.482 eÅ^{-3}	
R.M.S. deviation from mean	0.079 eÅ^{-3}	

Table S3: Bond lengths (Å) for (2*r*,5*s*)-**7a**

Si1A-O1A	1.640(3)	Si1A-C22A	1.859(5)
Si1A-C21A	1.860(5)	Si1A-C23A	1.893(5)
O1A-C5A	1.438(5)	O2A-N2A	1.227(5)
O3A-N2A	1.231(4)	N1A-C7A	1.465(6)
N1A-C1A	1.469(6)	N1A-H1AA	0.87(2)
N3A-C14A	1.451(5)	N3A-C3A	1.464(5)
N3A-H2AA	0.877(19)	N2A-C2A	1.508(5)
C5A-C6A	1.511(6)	C5A-C4A	1.531(5)
C5A-H5A	1.0	C6A-C1A	1.538(6)
C6A-H611	0.99	C6A-H612	0.99
C1A-C2A	1.525(5)	C1A-H1A	1.0
C2A-C3A	1.518(5)	C2A-H2A	1.0
C3A-C4A	1.531(6)	C3A-H3A	1.0
C4A-H411	0.99	C4A-H412	0.99
C21A-H21A	0.98	C21A-H21B	0.98
C21A-H21C	0.98	C22A-H22A	0.98
C22A-H22B	0.98	C22A-H22C	0.98
C23A-C26A	1.523(6)	C23A-C24A	1.533(6)

C23A-C25A	1.539(6)	C24A-H24A	0.98
C24A-H24B	0.98	C24A-H24C	0.98
C25A-H25A	0.98	C25A-H25B	0.98
C25A-H25C	0.98	C26A-H26A	0.98
C26A-H26B	0.98	C26A-H26C	0.98
C7A-C8A	1.510(6)	C7A-H7A	0.99
C7A-H7B	0.99	C14A-C15A	1.522(5)
C14A-H14A	0.99	C14A-H14B	0.99
C8A-C9A	1.384(6)	C8A-C13A	1.404(6)
C9A-C10A	1.388(6)	C9A-H9A	0.95
C10A-C11A	1.383(6)	C10A-H10A	0.95
C11A-C12A	1.390(7)	C11A-H11A	0.95
C12A-C13A	1.384(7)	C12A-H12A	0.95
C13A-H13A	0.95	C15A-C16A	1.388(6)
C15A-C20A	1.394(6)	C16A-C17A	1.390(6)
C16A-H16A	0.95	C17A-C18A	1.381(6)
C17A-H17A	0.95	C18A-C19A	1.372(6)
C18A-H18A	0.95	C19A-C20A	1.391(6)
C19A-H19A	0.95	C20A-H20A	0.95
O1B-C5B	1.433(5)	O1B-Si1E	1.633(11)
O1B-Si1B	1.650(4)	Si1B-C22B	1.857(6)
Si1B-C21B	1.864(6)	Si1B-C23B	1.870(6)
C21B-H21D	0.98	C21B-H21E	0.98
C21B-H21F	0.98	C22B-H22D	0.98
C22B-H22E	0.98	C22B-H22F	0.98
C23B-C24B	1.538(9)	C23B-C25B	1.545(8)
C23B-C26B	1.559(8)	C24B-H24J	0.98
C24B-H24K	0.98	C24B-H24L	0.98
C25B-H25J	0.98	C25B-H25K	0.98
C25B-H25L	0.98	C26B-H26J	0.98
C26B-H26K	0.98	C26B-H26L	0.98
Si1E-C23E	1.866(13)	Si1E-C22E	1.870(13)
Si1E-C21E	1.876(14)	C21E-H21G	0.98
C21E-H21H	0.98	C21E-H21I	0.98
C22E-H22G	0.98	C22E-H22H	0.98
C22E-H22I	0.98	C23E-C25E	1.549(14)
C23E-C24E	1.549(14)	C23E-C26E	1.561(14)
C24E-H24M	0.98	C24E-H24N	0.98
C24E-H24O	0.98	C25E-H25M	0.98
C25E-H25N	0.98	C25E-H25O	0.98
C26E-H26M	0.98	C26E-H26N	0.98
C26E-H26O	0.98	O2B-N2B	1.225(5)
O3B-N2B	1.231(5)	N1B-C7B	1.455(5)
N1B-C1B	1.464(5)	N1B-H1BB	0.82(5)
N3B-C14B	1.452(5)	N3B-C3B	1.456(5)
N3B-H2BB	0.86(6)	N2B-C2B	1.513(5)
C5B-C4B	1.519(6)	C5B-C6B	1.531(6)
C5B-H5B	1.0	C6B-C1B	1.536(6)
C6B-H621	0.99	C6B-H622	0.99

C1B-C2B	1.521(5)	C1B-H1B	1.0
C2B-C3B	1.521(5)	C2B-H2B	1.0
C3B-C4B	1.531(6)	C3B-H3B	1.0
C4B-H421	0.99	C4B-H422	0.99
C7B-C8B	1.512(6)	C7B-H7C	0.99
C7B-H7D	0.99	C14B-C15B	1.519(6)
C14B-H14C	0.99	C14B-H14D	0.99
C8B-C9B	1.375(6)	C8B-C13B	1.405(6)
C9B-C10B	1.390(6)	C9B-H9B	0.95
C10B-C11B	1.380(6)	C10B-H10B	0.95
C11B-C12B	1.383(6)	C11B-H11B	0.95
C12B-C13B	1.381(6)	C12B-H12B	0.95
C13B-H13B	0.95	C15B-C20B	1.389(6)
C15B-C16B	1.396(6)	C16B-C17B	1.389(6)
C16B-H16B	0.95	C17B-C18B	1.391(6)
C17B-H17B	0.95	C18B-C19B	1.386(6)
C18B-H18B	0.95	C19B-C20B	1.394(6)
C19B-H19B	0.95	C20B-H20B	0.95
Si1C-O1C	1.646(3)	Si1C-C21C	1.852(5)
Si1C-C22C	1.869(5)	Si1C-C23C	1.897(5)
O1C-C5C	1.440(5)	O2C-N2C	1.232(5)
O3C-N2C	1.232(4)	N1C-C7C	1.452(5)
N1C-C1C	1.466(5)	N1C-H1CC	0.90(6)
N3C-C14C	1.452(6)	N3C-C3C	1.464(5)
N3C-H2CC	0.87(5)	N2C-C2C	1.507(5)
C5C-C6C	1.514(5)	C5C-C4C	1.516(6)
C5C-H5C	1.0	C6C-C1C	1.535(6)
C6C-H641	0.99	C6C-H642	0.99
C1C-C2C	1.523(6)	C1C-H1C	1.0
C2C-C3C	1.525(5)	C2C-H2C	1.0
C3C-C4C	1.531(6)	C3C-H3C	1.0
C4C-H441	0.99	C4C-H442	0.99
C21C-H21J	0.98	C21C-H21K	0.98
C21C-H21L	0.98	C22C-H22J	0.98
C22C-H22K	0.98	C22C-H22L	0.98
C23C-C24C	1.529(6)	C23C-C25C	1.537(6)
C23C-C26C	1.540(6)	C24C-H24D	0.98
C24C-H24E	0.98	C24C-H24F	0.98
C25C-H25D	0.98	C25C-H25E	0.98
C25C-H25F	0.98	C26C-H26D	0.98
C26C-H26E	0.98	C26C-H26F	0.98
C7C-C8C	1.521(5)	C7C-H7E	0.99
C7C-H7F	0.99	C14C-C15C	1.511(6)
C14C-H14E	0.99	C14C-H14F	0.99
C8C-C13C	1.383(6)	C8C-C9C	1.394(6)
C9C-C10C	1.394(6)	C9C-H9C	0.95
C10C-C11C	1.381(7)	C10C-H10C	0.95
C11C-C12C	1.368(7)	C11C-H11C	0.95
C12C-C13C	1.402(6)	C12C-H12C	0.95

C13C-H13C	0.95	C15C-C16C	1.388(6)
C15C-C20C	1.403(7)	C16C-C17C	1.389(7)
C16C-H16C	0.95	C17C-C18C	1.389(7)
C17C-H17C	0.95	C18C-C19C	1.389(7)
C18C-H18C	0.95	C19C-C20C	1.375(7)
C19C-H19C	0.95	C20C-H20C	0.95
Si1D-O1D	1.648(3)	Si1D-C21D	1.834(5)
Si1D-C22D	1.867(6)	Si1D-C23D	1.890(4)
O1D-C5D	1.439(5)	O2D-N2D	1.225(5)
O3D-N2D	1.230(5)	N1D-C7D	1.453(5)
N1D-C1D	1.463(5)	N1D-H1DD	0.87(6)
N3D-C14D	1.455(5)	N3D-C3D	1.467(5)
N3D-H2DD	0.83(5)	N2D-C2D	1.513(5)
C5D-C6D	1.520(6)	C5D-C4D	1.526(6)
C5D-H5D	1.0	C6D-C1D	1.527(6)
C6D-H651	0.99	C6D-H652	0.99
C1D-C2D	1.524(5)	C1D-H1D	1.0
C2D-C3D	1.517(5)	C2D-H2D	1.0
C3D-C4D	1.545(6)	C3D-H3D	1.0
C4D-H451	0.99	C4D-H452	0.99
C21D-H21M	0.98	C21D-H21N	0.98
C21D-H21O	0.98	C22D-H22M	0.98
C22D-H22N	0.98	C22D-H22O	0.98
C23D-C26D	1.527(6)	C23D-C25D	1.529(6)
C23D-C24D	1.551(6)	C24D-H24G	0.98
C24D-H24H	0.98	C24D-H24I	0.98
C25D-H25G	0.98	C25D-H25H	0.98
C25D-H25I	0.98	C26D-H26G	0.98
C26D-H26H	0.98	C26D-H26I	0.98
C7D-C8D	1.512(5)	C7D-H7G	0.99
C7D-H7H	0.99	C14D-C15D	1.514(6)
C14D-H14G	0.99	C14D-H14H	0.99
C8D-C13D	1.387(6)	C8D-C9D	1.398(6)
C9D-C10D	1.392(6)	C9D-H9D	0.95
C10D-C11D	1.383(6)	C10D-H10D	0.95
C11D-C12D	1.392(6)	C11D-H11D	0.95
C12D-C13D	1.399(6)	C12D-H12D	0.95
C13D-H13D	0.95	C15D-C16D	1.372(6)
C15D-C20D	1.407(6)	C16D-C17D	1.392(6)
C16D-H16D	0.95	C17D-C18D	1.389(6)
C17D-H17D	0.95	C18D-C19D	1.384(6)
C18D-H18D	0.95	C19D-C20D	1.382(7)
C19D-H19D	0.95	C20D-H20D	0.95

Table S4: Bond angles (°) for (2*r*,5*s*)-**7a**

O1A-Si1A-C22A	110.7(2)	O1A-Si1A-C21A	110.1(2)
C22A-Si1A-C21A	108.2(3)	O1A-Si1A-C23A	104.21(18)

C22A-Si1A-C23A	112.3(2)	C21A-Si1A-C23A	111.3(2)
C5A-O1A-Si1A	127.3(3)	C7A-N1A-C1A	115.6(3)
C7A-N1A-H1AA	113.(4)	C1A-N1A-H1AA	104.(4)
C14A-N3A-C3A	115.4(3)	C14A-N3A-H2AA	107.(3)
C3A-N3A-H2AA	114.(3)	O2A-N2A-O3A	123.4(4)
O2A-N2A-C2A	117.9(3)	O3A-N2A-C2A	118.7(3)
O1A-C5A-C6A	109.1(3)	O1A-C5A-C4A	108.0(3)
C6A-C5A-C4A	111.4(3)	O1A-C5A-H5A	109.4
C6A-C5A-H5A	109.4	C4A-C5A-H5A	109.4
C5A-C6A-C1A	111.4(3)	C5A-C6A-H611	109.4
C1A-C6A-H611	109.4	C5A-C6A-H612	109.4
C1A-C6A-H612	109.4	H611-C6A-H612	108.0
N1A-C1A-C2A	109.1(3)	N1A-C1A-C6A	115.3(3)
C2A-C1A-C6A	108.6(3)	N1A-C1A-H1A	107.9
C2A-C1A-H1A	107.9	C6A-C1A-H1A	107.9
N2A-C2A-C3A	108.8(3)	N2A-C2A-C1A	108.0(3)
C3A-C2A-C1A	113.2(3)	N2A-C2A-H2A	108.9
C3A-C2A-H2A	108.9	C1A-C2A-H2A	108.9
N3A-C3A-C2A	109.2(3)	N3A-C3A-C4A	114.9(3)
C2A-C3A-C4A	108.5(3)	N3A-C3A-H3A	108.0
C2A-C3A-H3A	108.0	C4A-C3A-H3A	108.0
C5A-C4A-C3A	112.4(3)	C5A-C4A-H411	109.1
C3A-C4A-H411	109.1	C5A-C4A-H412	109.1
C3A-C4A-H412	109.1	H411-C4A-H412	107.9
Si1A-C21A-H21A	109.5	Si1A-C21A-H21B	109.5
H21A-C21A-H21B	109.5	Si1A-C21A-H21C	109.5
H21A-C21A-H21C	109.5	H21B-C21A-H21C	109.5
Si1A-C22A-H22A	109.5	Si1A-C22A-H22B	109.5
H22A-C22A-H22B	109.5	Si1A-C22A-H22C	109.5
H22A-C22A-H22C	109.5	H22B-C22A-H22C	109.5
C26A-C23A-C24A	109.0(4)	C26A-C23A-C25A	109.2(4)
C24A-C23A-C25A	109.5(4)	C26A-C23A-Si1A	109.6(3)
C24A-C23A-Si1A	110.5(3)	C25A-C23A-Si1A	108.9(3)
C23A-C24A-H24A	109.5	C23A-C24A-H24B	109.5
H24A-C24A-H24B	109.5	C23A-C24A-H24C	109.5
H24A-C24A-H24C	109.5	H24B-C24A-H24C	109.5
C23A-C25A-H25A	109.5	C23A-C25A-H25B	109.5
H25A-C25A-H25B	109.5	C23A-C25A-H25C	109.5
H25A-C25A-H25C	109.5	H25B-C25A-H25C	109.5
C23A-C26A-H26A	109.5	C23A-C26A-H26B	109.5
H26A-C26A-H26B	109.5	C23A-C26A-H26C	109.5
H26A-C26A-H26C	109.5	H26B-C26A-H26C	109.5
N1A-C7A-C8A	110.0(4)	N1A-C7A-H7A	109.7
C8A-C7A-H7A	109.7	N1A-C7A-H7B	109.7
C8A-C7A-H7B	109.7	H7A-C7A-H7B	108.2
N3A-C14A-C15A	111.7(3)	N3A-C14A-H14A	109.3
C15A-C14A-H14A	109.3	N3A-C14A-H14B	109.3
C15A-C14A-H14B	109.3	H14A-C14A-H14B	108.0
C9A-C8A-C13A	118.9(4)	C9A-C8A-C7A	121.7(4)

C13A-C8A-C7A	119.4(4)	C8A-C9A-C10A	121.1(4)
C8A-C9A-H9A	119.5	C10A-C9A-H9A	119.5
C11A-C10A-C9A	119.7(4)	C11A-C10A-H10A	120.1
C9A-C10A-H10A	120.1	C10A-C11A-C12A	119.9(4)
C10A-C11A-H11A	120.1	C12A-C11A-H11A	120.1
C13A-C12A-C11A	120.4(4)	C13A-C12A-H12A	119.8
C11A-C12A-H12A	119.8	C12A-C13A-C8A	120.0(4)
C12A-C13A-H13A	120.0	C8A-C13A-H13A	120.0
C16A-C15A-C20A	119.1(4)	C16A-C15A-C14A	119.8(4)
C20A-C15A-C14A	121.1(4)	C15A-C16A-C17A	120.6(4)
C15A-C16A-H16A	119.7	C17A-C16A-H16A	119.7
C18A-C17A-C16A	120.0(4)	C18A-C17A-H17A	120.0
C16A-C17A-H17A	120.0	C19A-C18A-C17A	119.5(4)
C19A-C18A-H18A	120.2	C17A-C18A-H18A	120.2
C18A-C19A-C20A	121.3(4)	C18A-C19A-H19A	119.4
C20A-C19A-H19A	119.4	C19A-C20A-C15A	119.4(4)
C19A-C20A-H20A	120.3	C15A-C20A-H20A	120.3
C5B-O1B-Si1E	133.8(5)	C5B-O1B-Si1B	126.0(3)
O1B-Si1B-C22B	111.4(3)	O1B-Si1B-C21B	110.1(3)
C22B-Si1B-C21B	108.5(3)	O1B-Si1B-C23B	102.7(3)
C22B-Si1B-C23B	112.3(3)	C21B-Si1B-C23B	111.8(3)
Si1B-C21B-H21D	109.5	Si1B-C21B-H21E	109.5
H21D-C21B-H21E	109.5	Si1B-C21B-H21F	109.5
H21D-C21B-H21F	109.5	H21E-C21B-H21F	109.5
Si1B-C22B-H22D	109.5	Si1B-C22B-H22E	109.5
H22D-C22B-H22E	109.5	Si1B-C22B-H22F	109.5
H22D-C22B-H22F	109.5	H22E-C22B-H22F	109.5
C24B-C23B-C25B	110.3(6)	C24B-C23B-C26B	109.1(6)
C25B-C23B-C26B	108.9(6)	C24B-C23B-Si1B	108.7(5)
C25B-C23B-Si1B	111.1(5)	C26B-C23B-Si1B	108.5(5)
C23B-C24B-H24J	109.5	C23B-C24B-H24K	109.5
H24J-C24B-H24K	109.5	C23B-C24B-H24L	109.5
H24J-C24B-H24L	109.5	H24K-C24B-H24L	109.5
C23B-C25B-H25J	109.5	C23B-C25B-H25K	109.5
H25J-C25B-H25K	109.5	C23B-C25B-H25L	109.5
H25J-C25B-H25L	109.5	H25K-C25B-H25L	109.5
C23B-C26B-H26J	109.5	C23B-C26B-H26K	109.5
H26J-C26B-H26K	109.5	C23B-C26B-H26L	109.5
H26J-C26B-H26L	109.5	H26K-C26B-H26L	109.5
O1B-Si1E-C23E	111.0(8)	O1B-Si1E-C22E	107.0(10)
C23E-Si1E-C22E	111.0(11)	O1B-Si1E-C21E	108.4(11)
C23E-Si1E-C21E	110.3(12)	C22E-Si1E-C21E	109.0(13)
Si1E-C21E-H21G	109.5	Si1E-C21E-H21H	109.5
H21G-C21E-H21H	109.5	Si1E-C21E-H21I	109.5
H21G-C21E-H21I	109.5	H21H-C21E-H21I	109.5
Si1E-C22E-H22G	109.5	Si1E-C22E-H22H	109.5
H22G-C22E-H22H	109.5	Si1E-C22E-H22I	109.5
H22G-C22E-H22I	109.5	H22H-C22E-H22I	109.5
C25E-C23E-C24E	107.5(17)	C25E-C23E-C26E	106.2(16)

C24E-C23E-C26E	113.6(16)	C25E-C23E-Si1E	112.0(15)
C24E-C23E-Si1E	106.9(14)	C26E-C23E-Si1E	110.7(13)
C23E-C24E-H24M	109.5	C23E-C24E-H24N	109.5
H24M-C24E-H24N	109.5	C23E-C24E-H24O	109.5
H24M-C24E-H24O	109.5	H24N-C24E-H24O	109.5
C23E-C25E-H25M	109.5	C23E-C25E-H25N	109.5
H25M-C25E-H25N	109.5	C23E-C25E-H25O	109.5
H25M-C25E-H25O	109.5	H25N-C25E-H25O	109.5
C23E-C26E-H26M	109.5	C23E-C26E-H26N	109.5
H26M-C26E-H26N	109.5	C23E-C26E-H26O	109.5
H26M-C26E-H26O	109.5	H26N-C26E-H26O	109.5
C7B-N1B-C1B	115.9(3)	C7B-N1B-H1BB	115.(4)
C1B-N1B-H1BB	108.(4)	C14B-N3B-C3B	114.6(3)
C14B-N3B-H2BB	112.(4)	C3B-N3B-H2BB	109.(4)
O2B-N2B-O3B	123.7(4)	O2B-N2B-C2B	117.7(3)
O3B-N2B-C2B	118.6(3)	O1B-C5B-C4B	108.2(3)
O1B-C5B-C6B	110.0(3)	C4B-C5B-C6B	110.6(3)
O1B-C5B-H5B	109.3	C4B-C5B-H5B	109.3
C6B-C5B-H5B	109.3	C5B-C6B-C1B	112.0(3)
C5B-C6B-H621	109.2	C1B-C6B-H621	109.2
C5B-C6B-H622	109.2	C1B-C6B-H622	109.2
H621-C6B-H622	107.9	N1B-C1B-C2B	109.4(3)
N1B-C1B-C6B	114.7(3)	C2B-C1B-C6B	108.4(3)
N1B-C1B-H1B	108.0	C2B-C1B-H1B	108.0
C6B-C1B-H1B	108.0	N2B-C2B-C3B	108.3(3)
N2B-C2B-C1B	107.9(3)	C3B-C2B-C1B	113.6(3)
N2B-C2B-H2B	109.0	C3B-C2B-H2B	109.0
C1B-C2B-H2B	109.0	N3B-C3B-C2B	109.4(3)
N3B-C3B-C4B	115.1(3)	C2B-C3B-C4B	106.9(3)
N3B-C3B-H3B	108.4	C2B-C3B-H3B	108.4
C4B-C3B-H3B	108.4	C5B-C4B-C3B	113.0(3)
C5B-C4B-H421	109.0	C3B-C4B-H421	109.0
C5B-C4B-H422	109.0	C3B-C4B-H422	109.0
H421-C4B-H422	107.8	N1B-C7B-C8B	109.7(3)
N1B-C7B-H7C	109.7	C8B-C7B-H7C	109.7
N1B-C7B-H7D	109.7	C8B-C7B-H7D	109.7
H7C-C7B-H7D	108.2	N3B-C14B-C15B	111.5(3)
N3B-C14B-H14C	109.3	C15B-C14B-H14C	109.3
N3B-C14B-H14D	109.3	C15B-C14B-H14D	109.3
H14C-C14B-H14D	108.0	C9B-C8B-C13B	118.9(4)
C9B-C8B-C7B	121.5(4)	C13B-C8B-C7B	119.6(4)
C8B-C9B-C10B	121.0(4)	C8B-C9B-H9B	119.5
C10B-C9B-H9B	119.5	C11B-C10B-C9B	119.6(4)
C11B-C10B-H10B	120.2	C9B-C10B-H10B	120.2
C10B-C11B-C12B	120.3(4)	C10B-C11B-H11B	119.9
C12B-C11B-H11B	119.9	C13B-C12B-C11B	120.0(4)
C13B-C12B-H12B	120.0	C11B-C12B-H12B	120.0
C12B-C13B-C8B	120.3(4)	C12B-C13B-H13B	119.9
C8B-C13B-H13B	119.9	C20B-C15B-C16B	119.1(4)

C20B-C15B-C14B	121.4(4)	C16B-C15B-C14B	119.5(4)
C17B-C16B-C15B	120.7(4)	C17B-C16B-H16B	119.6
C15B-C16B-H16B	119.6	C16B-C17B-C18B	119.6(4)
C16B-C17B-H17B	120.2	C18B-C17B-H17B	120.2
C19B-C18B-C17B	120.3(4)	C19B-C18B-H18B	119.9
C17B-C18B-H18B	119.9	C18B-C19B-C20B	119.8(4)
C18B-C19B-H19B	120.1	C20B-C19B-H19B	120.1
C15B-C20B-C19B	120.6(4)	C15B-C20B-H20B	119.7
C19B-C20B-H20B	119.7	O1C-Si1C-C21C	109.5(2)
O1C-Si1C-C22C	110.6(2)	C21C-Si1C-C22C	109.0(3)
O1C-Si1C-C23C	104.24(18)	C21C-Si1C-C23C	111.4(2)
C22C-Si1C-C23C	112.1(2)	C5C-O1C-Si1C	126.0(3)
C7C-N1C-C1C	114.4(3)	C7C-N1C-H1CC	110.(3)
C1C-N1C-H1CC	110.(3)	C14C-N3C-C3C	115.3(3)
C14C-N3C-H2CC	114.(3)	C3C-N3C-H2CC	106.(3)
O2C-N2C-O3C	123.0(4)	O2C-N2C-C2C	117.9(3)
O3C-N2C-C2C	119.1(3)	O1C-C5C-C6C	108.9(3)
O1C-C5C-C4C	108.9(3)	C6C-C5C-C4C	111.2(3)
O1C-C5C-H5C	109.3	C6C-C5C-H5C	109.3
C4C-C5C-H5C	109.3	C5C-C6C-C1C	113.0(3)
C5C-C6C-H641	109.0	C1C-C6C-H641	109.0
C5C-C6C-H642	109.0	C1C-C6C-H642	109.0
H641-C6C-H642	107.8	N1C-C1C-C2C	109.2(3)
N1C-C1C-C6C	115.1(3)	C2C-C1C-C6C	108.4(3)
N1C-C1C-H1C	108.0	C2C-C1C-H1C	108.0
C6C-C1C-H1C	108.0	N2C-C2C-C1C	109.1(3)
N2C-C2C-C3C	108.2(3)	C1C-C2C-C3C	113.1(3)
N2C-C2C-H2C	108.8	C1C-C2C-H2C	108.8
C3C-C2C-H2C	108.8	N3C-C3C-C2C	108.9(3)
N3C-C3C-C4C	115.6(3)	C2C-C3C-C4C	108.9(3)
N3C-C3C-H3C	107.7	C2C-C3C-H3C	107.7
C4C-C3C-H3C	107.7	C5C-C4C-C3C	111.5(3)
C5C-C4C-H441	109.3	C3C-C4C-H441	109.3
C5C-C4C-H442	109.3	C3C-C4C-H442	109.3
H441-C4C-H442	108.0	Si1C-C21C-H21J	109.5
Si1C-C21C-H21K	109.5	H21J-C21C-H21K	109.5
Si1C-C21C-H21L	109.5	H21J-C21C-H21L	109.5
H21K-C21C-H21L	109.5	Si1C-C22C-H22J	109.5
Si1C-C22C-H22K	109.5	H22J-C22C-H22K	109.5
Si1C-C22C-H22L	109.5	H22J-C22C-H22L	109.5
H22K-C22C-H22L	109.5	C24C-C23C-C25C	108.6(4)
C24C-C23C-C26C	110.1(4)	C25C-C23C-C26C	109.8(4)
C24C-C23C-Si1C	109.6(3)	C25C-C23C-Si1C	109.1(3)
C26C-C23C-Si1C	109.7(3)	C23C-C24C-H24D	109.5
C23C-C24C-H24E	109.5	H24D-C24C-H24E	109.5
C23C-C24C-H24F	109.5	H24D-C24C-H24F	109.5
H24E-C24C-H24F	109.5	C23C-C25C-H25D	109.5
C23C-C25C-H25E	109.5	H25D-C25C-H25E	109.5
C23C-C25C-H25F	109.5	H25D-C25C-H25F	109.5

H25E-C25C-H25F	109.5	C23C-C26C-H26D	109.5
C23C-C26C-H26E	109.5	H26D-C26C-H26E	109.5
C23C-C26C-H26F	109.5	H26D-C26C-H26F	109.5
H26E-C26C-H26F	109.5	N1C-C7C-C8C	112.3(3)
N1C-C7C-H7E	109.1	C8C-C7C-H7E	109.2
N1C-C7C-H7F	109.2	C8C-C7C-H7F	109.2
H7E-C7C-H7F	107.9	N3C-C14C-C15C	110.2(4)
N3C-C14C-H14E	109.6	C15C-C14C-H14E	109.6
N3C-C14C-H14F	109.6	C15C-C14C-H14F	109.6
H14E-C14C-H14F	108.1	C13C-C8C-C9C	119.2(4)
C13C-C8C-C7C	121.4(4)	C9C-C8C-C7C	119.4(4)
C10C-C9C-C8C	120.6(4)	C10C-C9C-H9C	119.7
C8C-C9C-H9C	119.7	C11C-C10C-C9C	119.7(4)
C11C-C10C-H10C	120.1	C9C-C10C-H10C	120.1
C12C-C11C-C10C	120.1(4)	C12C-C11C-H11C	120.0
C10C-C11C-H11C	120.0	C11C-C12C-C13C	120.8(4)
C11C-C12C-H12C	119.6	C13C-C12C-H12C	119.6
C8C-C13C-C12C	119.7(4)	C8C-C13C-H13C	120.2
C12C-C13C-H13C	120.2	C16C-C15C-C20C	118.5(4)
C16C-C15C-C14C	121.7(4)	C20C-C15C-C14C	119.8(4)
C15C-C16C-C17C	120.8(4)	C15C-C16C-H16C	119.6
C17C-C16C-H16C	119.6	C16C-C17C-C18C	120.3(4)
C16C-C17C-H17C	119.9	C18C-C17C-H17C	119.9
C19C-C18C-C17C	119.0(4)	C19C-C18C-H18C	120.5
C17C-C18C-H18C	120.5	C20C-C19C-C18C	121.0(5)
C20C-C19C-H19C	119.5	C18C-C19C-H19C	119.5
C19C-C20C-C15C	120.4(4)	C19C-C20C-H20C	119.8
C15C-C20C-H20C	119.8	O1D-Si1D-C21D	110.8(2)
O1D-Si1D-C22D	110.2(2)	C21D-Si1D-C22D	109.1(3)
O1D-Si1D-C23D	104.19(18)	C21D-Si1D-C23D	111.2(2)
C22D-Si1D-C23D	111.3(2)	C5D-O1D-Si1D	126.1(3)
C7D-N1D-C1D	114.4(3)	C7D-N1D-H1DD	114.(4)
C1D-N1D-H1DD	110.(4)	C14D-N3D-C3D	115.8(3)
C14D-N3D-H2DD	111.(3)	C3D-N3D-H2DD	112.(3)
O2D-N2D-O3D	123.6(4)	O2D-N2D-C2D	117.7(3)
O3D-N2D-C2D	118.7(3)	O1D-C5D-C6D	109.4(3)
O1D-C5D-C4D	108.8(3)	C6D-C5D-C4D	111.2(3)
O1D-C5D-H5D	109.2	C6D-C5D-H5D	109.2
C4D-C5D-H5D	109.2	C5D-C6D-C1D	113.2(3)
C5D-C6D-H651	108.9	C1D-C6D-H651	108.9
C5D-C6D-H652	108.9	C1D-C6D-H652	108.9
H651-C6D-H652	107.7	N1D-C1D-C2D	109.4(3)
N1D-C1D-C6D	115.1(3)	C2D-C1D-C6D	108.3(3)
N1D-C1D-H1D	107.9	C2D-C1D-H1D	107.9
C6D-C1D-H1D	107.9	N2D-C2D-C3D	107.8(3)
N2D-C2D-C1D	109.2(3)	C3D-C2D-C1D	113.1(3)
N2D-C2D-H2D	108.9	C3D-C2D-H2D	108.9
C1D-C2D-H2D	108.9	N3D-C3D-C2D	109.1(3)
N3D-C3D-C4D	114.7(3)	C2D-C3D-C4D	108.7(3)

N3D-C3D-H3D	108.1	C2D-C3D-H3D	108.1
C4D-C3D-H3D	108.1	C5D-C4D-C3D	110.6(3)
C5D-C4D-H451	109.5	C3D-C4D-H451	109.5
C5D-C4D-H452	109.5	C3D-C4D-H452	109.5
H451-C4D-H452	108.1	Si1D-C21D-H21M	109.5
Si1D-C21D-H21N	109.5	H21M-C21D-H21N	109.5
Si1D-C21D-H21O	109.5	H21M-C21D-H21O	109.5
H21N-C21D-H21O	109.5	Si1D-C22D-H22M	109.5
Si1D-C22D-H22N	109.5	H22M-C22D-H22N	109.5
Si1D-C22D-H22O	109.5	H22M-C22D-H22O	109.5
H22N-C22D-H22O	109.5	C26D-C23D-C25D	109.3(4)
C26D-C23D-C24D	108.6(4)	C25D-C23D-C24D	110.1(4)
C26D-C23D-Si1D	109.4(3)	C25D-C23D-Si1D	109.6(3)
C24D-C23D-Si1D	109.8(3)	C23D-C24D-H24G	109.5
C23D-C24D-H24H	109.5	H24G-C24D-H24H	109.5
C23D-C24D-H24I	109.5	H24G-C24D-H24I	109.5
H24H-C24D-H24I	109.5	C23D-C25D-H25G	109.5
C23D-C25D-H25H	109.5	H25G-C25D-H25H	109.5
C23D-C25D-H25I	109.5	H25G-C25D-H25I	109.5
H25H-C25D-H25I	109.5	C23D-C26D-H26G	109.5
C23D-C26D-H26H	109.5	H26G-C26D-H26H	109.5
C23D-C26D-H26I	109.5	H26G-C26D-H26I	109.5
H26H-C26D-H26I	109.5	N1D-C7D-C8D	112.5(3)
N1D-C7D-H7G	109.1	C8D-C7D-H7G	109.1
N1D-C7D-H7H	109.1	C8D-C7D-H7H	109.1
H7G-C7D-H7H	107.8	N3D-C14D-C15D	110.7(3)
N3D-C14D-H14G	109.5	C15D-C14D-H14G	109.5
N3D-C14D-H14H	109.5	C15D-C14D-H14H	109.5
H14G-C14D-H14H	108.1	C13D-C8D-C9D	119.2(4)
C13D-C8D-C7D	121.8(4)	C9D-C8D-C7D	119.1(4)
C10D-C9D-C8D	120.4(4)	C10D-C9D-H9D	119.8
C8D-C9D-H9D	119.8	C11D-C10D-C9D	120.2(4)
C11D-C10D-H10D	119.9	C9D-C10D-H10D	119.9
C10D-C11D-C12D	119.9(4)	C10D-C11D-H11D	120.0
C12D-C11D-H11D	120.0	C11D-C12D-C13D	119.8(4)
C11D-C12D-H12D	120.1	C13D-C12D-H12D	120.1
C8D-C13D-C12D	120.5(4)	C8D-C13D-H13D	119.8
C12D-C13D-H13D	119.8	C16D-C15D-C20D	119.4(4)
C16D-C15D-C14D	121.7(4)	C20D-C15D-C14D	118.9(4)
C15D-C16D-C17D	121.0(4)	C15D-C16D-H16D	119.5
C17D-C16D-H16D	119.5	C18D-C17D-C16D	119.6(4)
C18D-C17D-H17D	120.2	C16D-C17D-H17D	120.2
C19D-C18D-C17D	119.7(4)	C19D-C18D-H18D	120.1
C17D-C18D-H18D	120.1	C20D-C19D-C18D	120.7(4)
C20D-C19D-H19D	119.6	C18D-C19D-H19D	119.6
C19D-C20D-C15D	119.6(4)	C19D-C20D-H20D	120.2
C15D-C20D-H20D	120.2		

Table S5: Torsion angles (°) for (2*r*,5*s*)-**7a**

C22A-Si1A-O1A-C5A	25.4(4)	C21A-Si1A-O1A-C5A	-94.3(4)
C23A-Si1A-O1A-C5A	146.3(3)	Si1A-O1A-C5A-C6A	-105.2(4)
Si1A-O1A-C5A-C4A	133.5(3)	O1A-C5A-C6A-C1A	-63.8(4)
C4A-C5A-C6A-C1A	55.4(5)	C7A-N1A-C1A-C2A	142.9(4)
C7A-N1A-C1A-C6A	-94.8(5)	C5A-C6A-C1A-N1A	-179.0(3)
C5A-C6A-C1A-C2A	-56.4(4)	O2A-N2A-C2A-C3A	66.9(4)
O3A-N2A-C2A-C3A	-114.0(4)	O2A-N2A-C2A-C1A	-56.3(5)
O3A-N2A-C2A-C1A	122.7(4)	N1A-C1A-C2A-N2A	-54.3(4)
C6A-C1A-C2A-N2A	179.4(3)	N1A-C1A-C2A-C3A	-174.8(3)
C6A-C1A-C2A-C3A	58.9(4)	C14A-N3A-C3A-C2A	-163.5(3)
C14A-N3A-C3A-C4A	74.4(5)	N2A-C2A-C3A-N3A	56.3(4)
C1A-C2A-C3A-N3A	176.4(3)	N2A-C2A-C3A-C4A	-177.9(3)
C1A-C2A-C3A-C4A	-57.7(4)	O1A-C5A-C4A-C3A	65.0(4)
C6A-C5A-C4A-C3A	-54.9(5)	N3A-C3A-C4A-C5A	177.0(3)
C2A-C3A-C4A-C5A	54.5(4)	O1A-Si1A-C23A-C26A	47.7(3)
C22A-Si1A-C23A-C26A	167.5(3)	C21A-Si1A-C23A-C26A	-71.0(4)
O1A-Si1A-C23A-C24A	167.9(3)	C22A-Si1A-C23A-C24A	-72.3(4)
C21A-Si1A-C23A-C24A	49.2(4)	O1A-Si1A-C23A-C25A	-71.8(3)
C22A-Si1A-C23A-C25A	48.1(4)	C21A-Si1A-C23A-C25A	169.6(3)
C1A-N1A-C7A-C8A	-176.3(4)	C3A-N3A-C14A-C15A	170.7(3)
N1A-C7A-C8A-C9A	-115.4(5)	N1A-C7A-C8A-C13A	62.0(5)
C13A-C8A-C9A-C10A	-0.5(6)	C7A-C8A-C9A-C10A	176.9(4)
C8A-C9A-C10A-C11A	0.3(7)	C9A-C10A-C11A-C12A	0.3(7)
C10A-C11A-C12A-C13A	-0.8(7)	C11A-C12A-C13A-C8A	0.5(7)
C9A-C8A-C13A-C12A	0.1(6)	C7A-C8A-C13A-C12A	-177.4(4)
N3A-C14A-C15A-C16A	157.9(4)	N3A-C14A-C15A-C20A	-22.3(5)
C20A-C15A-C16A-C17A	0.5(6)	C14A-C15A-C16A-C17A	-179.8(4)
C15A-C16A-C17A-C18A	0.3(7)	C16A-C17A-C18A-C19A	-0.3(7)
C17A-C18A-C19A-C20A	-0.5(7)	C18A-C19A-C20A-C15A	1.3(6)
C16A-C15A-C20A-C19A	-1.2(6)	C14A-C15A-C20A-C19A	179.0(4)
C5B-O1B-Si1B-C22B	-39.3(4)	C5B-O1B-Si1B-C21B	81.1(4)
C5B-O1B-Si1B-C23B	-159.7(4)	O1B-Si1B-C23B-C24B	-63.0(5)
C22B-Si1B-C23B-C24B	177.2(4)	C21B-Si1B-C23B-C24B	55.0(6)
O1B-Si1B-C23B-C25B	175.4(5)	C22B-Si1B-C23B-C25B	55.6(7)
C21B-Si1B-C23B-C25B	-66.6(7)	O1B-Si1B-C23B-C26B	55.6(5)
C22B-Si1B-C23B-C26B	-64.2(6)	C21B-Si1B-C23B-C26B	173.6(5)
C5B-O1B-Si1E-C23E	-142.4(8)	C5B-O1B-Si1E-C22E	-21.2(13)
C5B-O1B-Si1E-C21E	96.2(12)	O1B-Si1E-C23E-C25E	-46.4(18)
C22E-Si1E-C23E-C25E	-165.2(18)	C21E-Si1E-C23E-C25E	73.8(19)
O1B-Si1E-C23E-C24E	71.1(16)	C22E-Si1E-C23E-C24E	-47.7(18)
C21E-Si1E-C23E-C24E	-168.7(17)	O1B-Si1E-C23E-C26E	-164.7(13)
C22E-Si1E-C23E-C26E	76.5(18)	C21E-Si1E-C23E-C26E	-44.5(19)
Si1E-O1B-C5B-C4B	162.7(6)	Si1B-O1B-C5B-C4B	139.9(3)
Si1E-O1B-C5B-C6B	-76.4(7)	Si1B-O1B-C5B-C6B	-99.2(4)
O1B-C5B-C6B-C1B	-65.5(4)	C4B-C5B-C6B-C1B	53.9(5)
C7B-N1B-C1B-C2B	143.3(4)	C7B-N1B-C1B-C6B	-94.7(4)
C5B-C6B-C1B-N1B	-177.3(3)	C5B-C6B-C1B-C2B	-54.7(4)
O2B-N2B-C2B-C3B	66.7(4)	O3B-N2B-C2B-C3B	-114.2(4)

O2B-N2B-C2B-C1B	-56.7(5)	O3B-N2B-C2B-C1B	122.4(4)
N1B-C1B-C2B-N2B	-55.0(4)	C6B-C1B-C2B-N2B	179.3(3)
N1B-C1B-C2B-C3B	-175.1(3)	C6B-C1B-C2B-C3B	59.2(4)
C14B-N3B-C3B-C2B	-161.9(3)	C14B-N3B-C3B-C4B	77.9(5)
N2B-C2B-C3B-N3B	55.4(4)	C1B-C2B-C3B-N3B	175.3(3)
N2B-C2B-C3B-C4B	-179.5(3)	C1B-C2B-C3B-C4B	-59.6(4)
O1B-C5B-C4B-C3B	64.8(5)	C6B-C5B-C4B-C3B	-55.8(5)
N3B-C3B-C4B-C5B	178.7(3)	C2B-C3B-C4B-C5B	57.0(4)
C1B-N1B-C7B-C8B	-176.7(4)	C3B-N3B-C14B-C15B	171.6(3)
N1B-C7B-C8B-C9B	-113.8(5)	N1B-C7B-C8B-C13B	63.4(5)
C13B-C8B-C9B-C10B	-0.5(6)	C7B-C8B-C9B-C10B	176.7(4)
C8B-C9B-C10B-C11B	0.1(6)	C9B-C10B-C11B-C12B	0.1(6)
C10B-C11B-C12B-C13B	0.2(6)	C11B-C12B-C13B-C8B	-0.7(6)
C9B-C8B-C13B-C12B	0.9(6)	C7B-C8B-C13B-C12B	-176.4(4)
N3B-C14B-C15B-C20B	-23.8(5)	N3B-C14B-C15B-C16B	156.3(4)
C20B-C15B-C16B-C17B	0.2(6)	C14B-C15B-C16B-C17B	-179.9(4)
C15B-C16B-C17B-C18B	0.8(6)	C16B-C17B-C18B-C19B	-1.0(6)
C17B-C18B-C19B-C20B	0.2(6)	C16B-C15B-C20B-C19B	-1.0(6)
C14B-C15B-C20B-C19B	179.1(4)	C18B-C19B-C20B-C15B	0.9(6)
C21C-Si1C-O1C-C5C	94.3(4)	C22C-Si1C-O1C-C5C	-25.7(4)
C23C-Si1C-O1C-C5C	-146.4(3)	Si1C-O1C-C5C-C6C	-133.0(3)
Si1C-O1C-C5C-C4C	105.6(4)	O1C-C5C-C6C-C1C	-65.3(4)
C4C-C5C-C6C-C1C	54.7(5)	C7C-N1C-C1C-C2C	167.5(3)
C7C-N1C-C1C-C6C	-70.3(5)	C5C-C6C-C1C-N1C	-176.8(3)
C5C-C6C-C1C-C2C	-54.2(4)	O2C-N2C-C2C-C1C	-68.1(4)
O3C-N2C-C2C-C1C	112.6(4)	O2C-N2C-C2C-C3C	55.3(5)
O3C-N2C-C2C-C3C	-124.0(4)	N1C-C1C-C2C-N2C	-56.8(4)
C6C-C1C-C2C-N2C	177.1(3)	N1C-C1C-C2C-C3C	-177.3(3)
C6C-C1C-C2C-C3C	56.6(4)	C14C-N3C-C3C-C2C	-145.4(4)
C14C-N3C-C3C-C4C	91.7(5)	N2C-C2C-C3C-N3C	53.8(4)
C1C-C2C-C3C-N3C	174.8(3)	N2C-C2C-C3C-C4C	-179.3(3)
C1C-C2C-C3C-C4C	-58.4(4)	O1C-C5C-C4C-C3C	64.6(4)
C6C-C5C-C4C-C3C	-55.4(5)	N3C-C3C-C4C-C5C	179.3(3)
C2C-C3C-C4C-C5C	56.4(4)	O1C-Si1C-C23C-C24C	-53.3(4)
C21C-Si1C-C23C-C24C	64.7(4)	C22C-Si1C-C23C-C24C	-172.9(3)
O1C-Si1C-C23C-C25C	65.5(3)	C21C-Si1C-C23C-C25C	-176.5(3)
C22C-Si1C-C23C-C25C	-54.2(4)	O1C-Si1C-C23C-C26C	-174.3(3)
C21C-Si1C-C23C-C26C	-56.3(4)	C22C-Si1C-C23C-C26C	66.1(4)
C1C-N1C-C7C-C8C	-171.4(3)	C3C-N3C-C14C-C15C	174.6(4)
N1C-C7C-C8C-C13C	19.9(6)	N1C-C7C-C8C-C9C	-160.2(4)
C13C-C8C-C9C-C10C	-0.7(6)	C7C-C8C-C9C-C10C	179.4(4)
C8C-C9C-C10C-C11C	-0.4(7)	C9C-C10C-C11C-C12C	0.8(7)
C10C-C11C-C12C-C13C	-0.2(7)	C9C-C8C-C13C-C12C	1.3(6)
C7C-C8C-C13C-C12C	-178.8(4)	C11C-C12C-C13C-C8C	-0.8(7)
N3C-C14C-C15C-C16C	117.5(5)	N3C-C14C-C15C-C20C	-60.6(6)
C20C-C15C-C16C-C17C	-0.7(7)	C14C-C15C-C16C-C17C	-178.7(4)
C15C-C16C-C17C-C18C	0.2(7)	C16C-C17C-C18C-C19C	0.1(7)
C17C-C18C-C19C-C20C	0.1(7)	C18C-C19C-C20C-C15C	-0.6(7)
C16C-C15C-C20C-C19C	0.8(7)	C14C-C15C-C20C-C19C	178.9(4)

C21D-Si1D-O1D-C5D	90.2(4)	C22D-Si1D-O1D-C5D	-30.6(4)
C23D-Si1D-O1D-C5D	-150.1(3)	Si1D-O1D-C5D-C6D	-125.7(3)
Si1D-O1D-C5D-C4D	112.8(3)	O1D-C5D-C6D-C1D	-65.2(4)
C4D-C5D-C6D-C1D	54.9(5)	C7D-N1D-C1D-C2D	167.8(3)
C7D-N1D-C1D-C6D	-70.0(4)	C5D-C6D-C1D-N1D	-177.0(3)
C5D-C6D-C1D-C2D	-54.3(4)	O2D-N2D-C2D-C3D	54.6(4)
O3D-N2D-C2D-C3D	-124.8(4)	O2D-N2D-C2D-C1D	-68.6(4)
O3D-N2D-C2D-C1D	112.0(4)	N1D-C1D-C2D-N2D	-56.4(4)
C6D-C1D-C2D-N2D	177.4(3)	N1D-C1D-C2D-C3D	-176.4(3)
C6D-C1D-C2D-C3D	57.4(4)	C14D-N3D-C3D-C2D	-141.8(4)
C14D-N3D-C3D-C4D	96.0(4)	N2D-C2D-C3D-N3D	53.9(4)
C1D-C2D-C3D-N3D	174.7(3)	N2D-C2D-C3D-C4D	179.6(3)
C1D-C2D-C3D-C4D	-59.6(4)	O1D-C5D-C4D-C3D	65.0(4)
C6D-C5D-C4D-C3D	-55.4(4)	N3D-C3D-C4D-C5D	179.5(3)
C2D-C3D-C4D-C5D	57.1(4)	O1D-Si1D-C23D-C26D	-52.7(3)
C21D-Si1D-C23D-C26D	66.7(4)	C22D-Si1D-C23D-C26D	-171.4(3)
O1D-Si1D-C23D-C25D	67.2(3)	C21D-Si1D-C23D-C25D	-173.5(4)
C22D-Si1D-C23D-C25D	-51.5(4)	O1D-Si1D-C23D-C24D	-171.8(3)
C21D-Si1D-C23D-C24D	-52.4(4)	C22D-Si1D-C23D-C24D	69.5(4)
C1D-N1D-C7D-C8D	-171.1(3)	C3D-N3D-C14D-C15D	175.5(4)
N1D-C7D-C8D-C13D	20.9(5)	N1D-C7D-C8D-C9D	-159.1(4)
C13D-C8D-C9D-C10D	-0.3(6)	C7D-C8D-C9D-C10D	179.7(4)
C8D-C9D-C10D-C11D	-0.9(7)	C9D-C10D-C11D-C12D	1.8(7)
C10D-C11D-C12D-C13D	-1.5(7)	C9D-C8D-C13D-C12D	0.5(6)
C7D-C8D-C13D-C12D	-179.5(4)	C11D-C12D-C13D-C8D	0.3(6)
N3D-C14D-C15D-C16D	113.0(5)	N3D-C14D-C15D-C20D	-64.3(5)
C20D-C15D-C16D-C17D	-0.7(6)	C14D-C15D-C16D-C17D	-178.0(4)
C15D-C16D-C17D-C18D	0.7(6)	C16D-C17D-C18D-C19D	-0.5(6)
C17D-C18D-C19D-C20D	0.4(6)	C18D-C19D-C20D-C15D	-0.4(6)
C16D-C15D-C20D-C19D	0.5(6)	C14D-C15D-C20D-C19D	177.9(4)

4.3. X-ray crystal structure analysis of (4a*RS*,5*SR*,7*SR*,8a*RS*)-1-benzyl-5-(benzylamino)-7-(*tert*-butyldimethyl-silyloxy)octahydroquinoxaline-2,3-dione (9), (dan8711)

A colorless plate-like specimen of $C_{28}H_{39}N_3O_3Si$, approximate dimensions 0.050 mm x 0.190 mm x 0.220 mm, was used for the X-ray crystallographic analysis. The X-ray intensity data were measured. The integration of the data using an orthorhombic unit cell yielded a total of 35209 reflections to a maximum θ angle of 67.12° (0.84 Å resolution), of which 4871 were independent (average redundancy 7.228, completeness = 98.6%, R_{int} = 6.06%, R_{sig} = 3.37%) and 4521 (92.81%) were greater than 2σ(F^2).

The final cell constants of a = 13.148(3) Å, b = 36.517(7) Å, c = 11.547(2) Å, volume = 5544.(2) Å^3, are based upon the refinement of the XYZ-centroids of reflections above 20 σ(I). The calculated minimum and maximum transmission coefficients (based on crystal size) are 0.8100 and 0.9520. The final anisotropic full-matrix least-squares refinement on F^2 with 329 variables converged at R1 = 3.03%, for the observed data and wR2 = 7.70% for all data. The goodness-of-fit was 1.053. The largest peak in the final difference electron density synthesis was 0.238 e^-/Å^3 and the largest hole was -0.206 e^-/Å^3 with an RMS deviation of 0.033 e^-/Å^3. On the basis of the final model, the calculated density was 1.183 g/cm^3 and F(000), 2128 e^-. The hydrogen atoms located at N2 and N4 were refined freely. CCDC Nr.: 2384409.

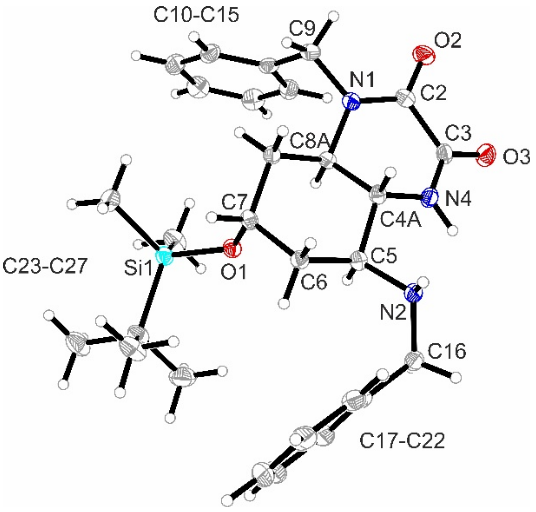

Figure S2: Crystal structure of racemic **9**. Thermal ellipsoids are shown at 50% probability.

Table S6: Sample and crystal data for **9**

Identification code	dan8711	
Chemical formula	$C_{28}H_{39}N_3O_3Si$	
Formula weight	493.71 g/mol	
Temperature	100(2) K	
Wavelength	1.54178 Å	
Crystal size	0.050 x 0.190 x 0.220 mm	
Crystal habit	colorless plate	
Crystal system	orthorhombic	
Space group	I b a 2	
Unit cell dimensions	a = 13.148(3) Å	α = 90°
	b = 36.517(7) Å	β = 90°

	c = 11.547(2) Å	γ = 90°
Volume	5544.(2) $Å^3$	
Z	8	
Density (calculated)	1.183 g/cm^3	
Absorption coefficient	1.002 mm^{-1}	
F(000)	2128	

Table S7: Data collection and structure refinement for **9**

Theta range for data collection	3.57 to 67.12°	
Index ranges	-15<=h<=14, -43<=k<=43, -13<=l<=13	
Reflections collected	35209	
Independent reflections	4871 [R(int) = 0.0606]	
Max. and min. transmission	0.9520 and 0.8100	
Refinement method	Full-matrix least-squares on F^2	
Refinement program	SHELXL-2014/7 (Sheldrick, 2014)	
Function minimized	$\Sigma w(F_o^2 - F_c^2)^2$	
Data / restraints / parameters	4871 / 1 / 329	
Goodness-of-fit on F^2	1.053	
Final R indices	4521 data; I>2σ(I)	R1 = 0.0303, wR2 = 0.0752
	all data	R1 = 0.0346, wR2 = 0.0770
Weighting scheme	$w=1/[\sigma^2(F_o^2)+(0.0457P)^2+0.4712P]$ where $P=(F_o^2+2F_c^2)/3$	
Absolute structure parameter	0.023(12)	
Largest diff. peak and hole	0.238 and -0.206 $eÅ^{-3}$	
R.M.S. deviation from mean	0.033 $eÅ^{-3}$	

Table S8: Bond lengths (Å) for **9**

N1-C2	1.356(3)	N1-C9	1.467(3)
N1-C8A	1.484(3)	N2-C5	1.456(3)
N2-C16	1.466(3)	N2-H2	0.85(3)
N4-C3	1.330(3)	N4-C4A	1.458(3)
N4-H4	0.90(3)	O1-C7	1.433(3)
O1-Si1	1.6499(17)	O2-C2	1.221(3)

O3-C3	1.225(3)	Si1-C24	1.849(3)
Si1-C23	1.873(3)	Si1-C25	1.894(3)
C7-C6	1.518(3)	C7-C8	1.530(3)
C7-H7A	1.0	C8-C8A	1.530(3)
C8-H8A	0.99	C8-H8B	0.99
C8A-C4A	1.515(3)	C8A-H8C	1.0
C4A-C5	1.530(3)	C4A-H4A	1.0
C5-C6	1.533(3)	C5-H5	1.0
C6-H6A	0.99	C6-H6B	0.99
C23-H23A	0.98	C23-H23B	0.98
C23-H23C	0.98	C24-H24A	0.98
C24-H24B	0.98	C24-H24C	0.98
C25-C27	1.526(4)	C25-C28	1.527(4)
C25-C26	1.538(4)	C26-H26A	0.98
C26-H26B	0.98	C26-H26C	0.98
C27-H27A	0.98	C27-H27B	0.98
C27-H27C	0.98	C28-H28A	0.98
C28-H28B	0.98	C28-H28C	0.98
C9-C10	1.509(4)	C9-H9A	0.99
C9-H9B	0.99	C16-C17	1.517(3)
C16-H16A	0.99	C16-H16B	0.99
C2-C3	1.550(4)	C10-C11	1.392(4)
C10-C15	1.396(4)	C11-C12	1.381(4)
C11-H11	0.95	C12-C13	1.387(5)
C12-H12	0.95	C13-C14	1.388(4)
C13-H13	0.95	C14-C15	1.385(4)
C14-H14	0.95	C15-H15	0.95
C17-C18	1.384(4)	C17-C22	1.398(4)
C18-C19	1.393(4)	C18-H18	0.95
C19-C20	1.379(5)	C19-H19	0.95
C20-C21	1.387(5)	C20-H20	0.95
C21-C22	1.381(4)	C21-H21	0.95
C22-H22	0.95		

Table S9: Bond angles (°) for **9**

C2-N1-C9	117.7(2)	C2-N1-C8A	120.3(2)
C9-N1-C8A	119.44(19)	C5-N2-C16	116.5(2)
C5-N2-H2	111.5(18)	C16-N2-H2	112.2(18)
C3-N4-C4A	123.8(2)	C3-N4-H4	119.(2)
C4A-N4-H4	117.(2)	C7-O1-Si1	126.68(14)
O1-Si1-C24	104.81(11)	O1-Si1-C23	111.27(11)

C24-Si1-C23	110.17(15)	O1-Si1-C25	108.13(11)
C24-Si1-C25	111.93(13)	C23-Si1-C25	110.40(13)
O1-C7-C6	108.00(19)	O1-C7-C8	108.98(19)
C6-C7-C8	111.88(19)	O1-C7-H7A	109.3
C6-C7-H7A	109.3	C8-C7-H7A	109.3
C7-C8-C8A	109.16(19)	C7-C8-H8A	109.8
C8A-C8-H8A	109.8	C7-C8-H8B	109.8
C8A-C8-H8B	109.8	H8A-C8-H8B	108.3
N1-C8A-C4A	108.49(18)	N1-C8A-C8	113.0(2)
C4A-C8A-C8	108.85(19)	N1-C8A-H8C	108.8
C4A-C8A-H8C	108.8	C8-C8A-H8C	108.8
N4-C4A-C8A	109.37(19)	N4-C4A-C5	108.1(2)
C8A-C4A-C5	111.74(19)	N4-C4A-H4A	109.2
C8A-C4A-H4A	109.2	C5-C4A-H4A	109.2
N2-C5-C4A	106.99(19)	N2-C5-C6	116.7(2)
C4A-C5-C6	108.66(19)	N2-C5-H5	108.1
C4A-C5-H5	108.1	C6-C5-H5	108.1
C7-C6-C5	112.7(2)	C7-C6-H6A	109.1
C5-C6-H6A	109.1	C7-C6-H6B	109.1
C5-C6-H6B	109.1	H6A-C6-H6B	107.8
Si1-C23-H23A	109.5	Si1-C23-H23B	109.5
H23A-C23-H23B	109.5	Si1-C23-H23C	109.5
H23A-C23-H23C	109.5	H23B-C23-H23C	109.5
Si1-C24-H24A	109.5	Si1-C24-H24B	109.5
H24A-C24-H24B	109.5	Si1-C24-H24C	109.5
H24A-C24-H24C	109.5	H24B-C24-H24C	109.5
C27-C25-C28	109.7(3)	C27-C25-C26	109.0(2)
C28-C25-C26	109.1(2)	C27-C25-Si1	108.77(18)
C28-C25-Si1	109.82(19)	C26-C25-Si1	110.5(2)
C25-C26-H26A	109.5	C25-C26-H26B	109.5
H26A-C26-H26B	109.5	C25-C26-H26C	109.5
H26A-C26-H26C	109.5	H26B-C26-H26C	109.5
C25-C27-H27A	109.5	C25-C27-H27B	109.5
H27A-C27-H27B	109.5	C25-C27-H27C	109.5
H27A-C27-H27C	109.5	H27B-C27-H27C	109.5
C25-C28-H28A	109.5	C25-C28-H28B	109.5
H28A-C28-H28B	109.5	C25-C28-H28C	109.5
H28A-C28-H28C	109.5	H28B-C28-H28C	109.5
N1-C9-C10	112.7(2)	N1-C9-H9A	109.1
C10-C9-H9A	109.1	N1-C9-H9B	109.1
C10-C9-H9B	109.1	H9A-C9-H9B	107.8
N2-C16-C17	116.9(2)	N2-C16-H16A	108.1
C17-C16-H16A	108.1	N2-C16-H16B	108.1

C17-C16-H16B	108.1	H16A-C16-H16B	107.3
O2-C2-N1	124.6(2)	O2-C2-C3	117.8(2)
N1-C2-C3	117.7(2)	O3-C3-N4	125.0(2)
O3-C3-C2	118.8(2)	N4-C3-C2	116.2(2)
C11-C10-C15	118.6(3)	C11-C10-C9	120.6(2)
C15-C10-C9	120.8(2)	C12-C11-C10	121.3(3)
C12-C11-H11	119.3	C10-C11-H11	119.3
C11-C12-C13	119.3(3)	C11-C12-H12	120.3
C13-C12-H12	120.3	C12-C13-C14	120.4(3)
C12-C13-H13	119.8	C14-C13-H13	119.8
C15-C14-C13	119.8(3)	C15-C14-H14	120.1
C13-C14-H14	120.1	C14-C15-C10	120.6(3)
C14-C15-H15	119.7	C10-C15-H15	119.7
C18-C17-C22	118.4(2)	C18-C17-C16	121.0(2)
C22-C17-C16	120.6(2)	C17-C18-C19	121.2(3)
C17-C18-H18	119.4	C19-C18-H18	119.4
C20-C19-C18	119.8(3)	C20-C19-H19	120.1
C18-C19-H19	120.1	C19-C20-C21	119.5(3)
C19-C20-H20	120.3	C21-C20-H20	120.3
C22-C21-C20	120.7(3)	C22-C21-H21	119.6
C20-C21-H21	119.6	C21-C22-C17	120.3(3)
C21-C22-H22	119.8	C17-C22-H22	119.8

Table S10: Torsion angles (°) for **9**

C7-O1-Si1-C24	140.0(2)	C7-O1-Si1-C23	21.0(2)
C7-O1-Si1-C25	-100.5(2)	Si1-O1-C7-C6	137.81(16)
Si1-O1-C7-C8	-100.4(2)	O1-C7-C8-C8A	-62.8(2)
C6-C7-C8-C8A	56.5(3)	C2-N1-C8A-C4A	43.5(3)
C9-N1-C8A-C4A	-154.9(2)	C2-N1-C8A-C8	164.3(2)
C9-N1-C8A-C8	-34.1(3)	C7-C8-C8A-N1	179.55(19)
C7-C8-C8A-C4A	-59.9(2)	C3-N4-C4A-C8A	36.8(3)
C3-N4-C4A-C5	158.6(2)	N1-C8A-C4A-N4	-54.8(3)
C8-C8A-C4A-N4	-178.12(19)	N1-C8A-C4A-C5	-174.43(19)
C8-C8A-C4A-C5	62.2(3)	C16-N2-C5-C4A	-156.3(2)
C16-N2-C5-C6	81.8(3)	N4-C4A-C5-N2	55.1(2)
C8A-C4A-C5-N2	175.5(2)	N4-C4A-C5-C6	-178.14(19)
C8A-C4A-C5-C6	-57.8(3)	O1-C7-C6-C5	65.8(2)
C8-C7-C6-C5	-54.1(3)	N2-C5-C6-C7	174.1(2)
C4A-C5-C6-C7	53.1(3)	O1-Si1-C25-C27	62.2(2)
C24-Si1-C25-C27	177.12(19)	C23-Si1-C25-C27	-59.8(2)
O1-Si1-C25-C28	-57.8(2)	C24-Si1-C25-C28	57.1(2)
C23-Si1-C25-C28	-179.8(2)	O1-Si1-C25-C26	-178.2(2)

C24-Si1-C25-C26	-63.2(2)	C23-Si1-C25-C26	59.9(2)
C2-N1-C9-C10	98.0(3)	C8A-N1-C9-C10	-64.1(3)
C5-N2-C16-C17	-61.7(3)	C9-N1-C2-O2	8.5(4)
C8A-N1-C2-O2	170.4(2)	C9-N1-C2-C3	-170.7(2)
C8A-N1-C2-C3	-8.8(3)	C4A-N4-C3-O3	178.1(2)
C4A-N4-C3-C2	-1.5(4)	O2-C2-C3-O3	-13.2(4)
N1-C2-C3-O3	166.0(2)	O2-C2-C3-N4	166.4(2)
N1-C2-C3-N4	-14.3(3)	N1-C9-C10-C11	134.4(2)
N1-C9-C10-C15	-46.6(3)	C15-C10-C11-C12	-0.5(4)
C9-C10-C11-C12	178.5(2)	C10-C11-C12-C13	0.4(4)
C11-C12-C13-C14	0.0(4)	C12-C13-C14-C15	-0.4(4)
C13-C14-C15-C10	0.3(4)	C11-C10-C15-C14	0.2(4)
C9-C10-C15-C14	-178.9(2)	N2-C16-C17-C18	122.8(3)
N2-C16-C17-C22	-58.0(3)	C22-C17-C18-C19	1.0(4)
C16-C17-C18-C19	-179.8(2)	C17-C18-C19-C20	-0.2(4)
C18-C19-C20-C21	-0.7(4)	C19-C20-C21-C22	0.8(4)
C20-C21-C22-C17	-0.1(4)	C18-C17-C22-C21	-0.8(4)
C16-C17-C22-C21	179.9(3)		

Table S11: Hydrogen bond distances (Å) and angles (°) for **9**

	Donor-H	Acceptor-H	Donor-Acceptor	Angle
N2-H2···O2	0.85(3)	2.19(3)	3.027(3)	168.(3)
C8-H8A···O3	0.99	2.45	3.412(3)	164.0

References X-Ray Part

1. APEX3 (V2016.1-0; **2016**), *SAINT* (V8.37A; **2015**) and *SADABS* (V2014/7; **2015**), Bruker AXS Inc., Madison, Wisconsin, USA.
2. Sheldrick, G. M., *SHELXT – Integrated space-group and crystal-structure determination*, *Acta Cryst.,* **2015**, *A71*, 3-8.
3. Sheldrick, G.M., *Crystal structure refinement with SHELXL*, *Acta Cryst.*, **2015**, *C71 (1)*, 3-8.
4. Bruker AXS (**1998**) *XP – Interactive molecular graphics, Version 5.1,* Bruker AXS Inc., Madison, Wisconsin, USA.

5. Receptor binding studies

5.1. Materials

Guinea pig brains, rat brains and rat livers were commercially available (Harlan-Winkelmann, Borchen, Germany). Homogenizers: Elvehjem Potter (B. Braun Biotech International, Melsungen, Germany) and Soniprep® 150 (MSE, London, UK). Centrifuges: Cooling centrifuge model Eppendorf 5427R (Eppendorf, Hamburg, Germany) and High-speed cooling centrifuge model Sorvall® RC-5C plus (Thermo Fisher Scientific, Langenselbold, Germany). Multiplates: standard 96 well multiplates (Diagonal, Muenster, Germany). Shaker: self-made device with adjustable temperature and tumbling speed (scientific workshop of the institute). Harvester: MicroBeta® FilterMate 96 Harvester. Filter: Printed Filtermat Typ A and B. Scintillator: Meltilex® (Typ A or B) solid state scintillator. Scintillation analyzer: MicroBeta® Trilux (all Perkin Elmer LAS, Rodgau-Jügesheim, Germany).

5.2. Preparation of membrane homogenates from guinea pig brain

5 guinea pig brains were homogenized with the potter (500-800 rpm, 10 up and down strokes) in 6 volumes of cold 0.32 M sucrose. The suspension was centrifuged at 1,200 x g for 10 min at 4 °C. The supernatant was separated and centrifuged at 23,500 x g for 20 min at 4 °C. The pellet was resuspended in 5-6 volumes of buffer (50 mM TRIS, pH 7.4) and centrifuged again at 23,500 x g (20 min, 4 °C). This procedure was repeated twice. The final pellet was resuspended in 5-6 volumes of buffer and frozen (-80 °C) in 1.5 mL portions containing about 1.5 mg protein/mL.

5.3. Preparation of membrane homogenates from rat brain

5 rat brains (species: Sprague Dawley rats, gender or sex unspecified) were homogenized with the potter (500-800 rpm, 10 up and down strokes) in 6 volumes of cold 0.32 M sucrose. The suspension was centrifuged at 1,200 x g for 10 min at 4 °C. The supernatant was separated and centrifuged at 23,500 x g for 20 min at 4 °C. The pellet was resuspended in 5-6 volumes of buffer (50 mM TRIS, pH 7.4) and centrifuged again at 23,500 x g (20 min, 4 °C). This procedure was repeated twice. The final pellet was resuspended in 5-6 volumes of buffer and frozen (-80 °C) in 1.5 mL portions containing about 1.5 mg protein/mL.

5.4. Preparation of membrane homogenates from rat liver

Two rat livers were cut into small pieces and homogenized with the potter (500-800 rpm, 10 up and down strokes) in 6 volumes of cold 0.32 M sucrose. The suspension was centrifuged at 1,200 x g for 10 min at 4 °C. The supernatant was separated and centrifuged at 31,000 x g for 20 min at 4 °C. The pellet was resuspended in 5-6 volumes of buffer (50 mM TRIS, pH 8.0) and incubated at rt for 30 min. After the incubation, the suspension was centrifuged again at 31,000 x g for 20 min at 4 °C. The final pellet was resuspended in 5-6 volumes of buffer and stored at -80 °C in 1.5 mL portions containing about 2 mg protein/mL.

5.5. Protein determination

The protein concentration was determined by the method of Bradford.[1] modified by Stoscheck.[2] The Bradford solution was prepared by dissolving 5 mg of Coomassie Brilliant Blue G 250 in 2.5 mL of EtOH (95 %, v/v). 10 mL deionized H_2O and 5 mL phosphoric acid (85 %, m/v) were added to this solution, the mixture was stirred and filled to a total volume of 50 mL with deionized water. The calibration was carried out using bovine serum albumin as a standard in 9 concentrations (0.1, 0.2, 0.4, 0.6, 0.8, 1.0, 1.5, 2.0 and 4.0 mg /mL). In a 96 well standard multiplate, 10 µL of the calibration solution or 10 µL of the membrane receptor preparation were mixed with 190 µL of the Bradford solution, respectively. After 5 min, the UV absorption of the protein-dye complex at λ = 595 nm was measured with a plate reader (Tecan Genios®, Tecan, Crailsheim, Germany).

5.6. General procedures for the binding assays

The test compound solutions were prepared by dissolving approximately 10 µmol (usually 2-4 mg) of test compound in DMSO so that a 10 mM stock solution was obtained. To obtain the required test solutions for the assay, the DMSO stock solution was diluted with the respective assay buffer. The filtermats were presoaked in 0.5 % aqueous polyethylenimine solution for 2 h at rt before use. All binding experiments were carried out in duplicates in the 96 well multiplates. The concentrations given are the final concentration in the assay. Generally, the assays were performed by addition of 50 µL of the respective assay buffer, 50 µL of test compound solution in various concentrations (10^{-5}, 10^{-6}, 10^{-7}, 10^{-8}, 10^{-9} and 10^{-10} mol/L), 50 µL of the corresponding radioligand solution and 50 µL of the respective receptor preparation into each well of

the multiplate (total volume 200 µL). The receptor preparation was always added last. During the incubation, the multiplates were shaken at a speed of 500-600 rpm at the specified temperature. Unless otherwise noted, the assays were terminated after 120 min by rapid filtration using the harvester. During the filtration, each well was washed five times with 300 µL of water. Subsequently, the filtermats were dried at 95 °C. The solid scintillator was melted on the dried filtermats at a temperature of 95 °C for 5 min. After solidifying of the scintillator at rt, the trapped radioactivity in the filtermats was measured with the scintillation analyzer. Each position on the filtermat corresponding to one well of the multiplate was measured for 5 min with the [^{3}H]-counting protocol. The overall counting efficiency was 20 %. The IC_{50} values were calculated with the program GraphPad Prism® 3.0 (GraphPad Software, San Diego, CA, USA) by non-linear regression analysis. Subsequently, the IC_{50} values were transformed into K_i values using the equation of Cheng and Prusoff.[3] The K_i values are given as mean value ± SEM from three independent experiments.

Performance of the binding assays

5.7. Affinity toward µ receptor (µ assay)

The assay was performed with the radioligand [^{3}H]DAMGO (51 Ci/mmol, Perkin Elmer). The thawed guinea pig brain membrane preparation (about 100 µg of the protein) was incubated with various concentrations of test compounds, 3 nM [^{3}H]DAMGO, and TRIS-$MgCl_2$-buffer (50 mM TRIS, 8 mM $MgCl_2$, pH 7.4) at 37 °C. The non-specific binding was determined with 10 µM unlabeled naloxone. The K_d value of [^{3}H]-DAMGO is 0.57 nM.

5.8. Affinity toward δ receptor (δ assay)

The assay was performed with the radioligand [^{3}H]DPDPE (69 Ci/mmol, BIOTREND). The thawed rat brain membrane preparation (about 75 µg of the protein) was incubated with various concentrations of test compounds, 3 nM [^{3}H]DPDPE, and TRIS-$MgCl_2$-buffer (50 mM TRIS, 8 mM $MgCl_2$, pH 7.4) supplemented with SIGMAFAST® protease inhibitor mix (Sigma Aldrich Biochemicals, Hamburg, Germany; 1 tablet dissolved in 100 mL of buffer) at 37 °C. The non-specific binding was determined with 10 µM unlabeled morphine. The K_d value of [^{3}H]-DPDPE is 0.65 nM.

5.9. Affinity toward σ_1 receptor (σ_1 assay)

The assay was performed with the radioligand [^{3}H]-(+)-pentazocine (22.0 Ci/mmol; Perkin Elmer). The thawed membrane preparation of guinea pig brain (about 100 µg of the protein) was incubated with various concentrations of test compounds, 2 nM [^{3}H]-(+)-pentazocine, and TRIS buffer (50 mM, pH 7.4) at 37 °C. The non-specific binding was determined with 10 µM unlabeled (+)-pentazocine. The K_d value of (+)-pentazocine is 2.9 nM.[4]

5.10. Affinity toward σ_2 receptor (σ_2 assay)

The assays were performed with the radioligand [^{3}H]di-*o*-tolylguanidine (specific activity 50 Ci/mmol; ARC, St. Louis, MO, USA). The thawed rat liver membrane preparation (about 100 µg protein) was incubated with various concentrations of the test compound, 3 nM [^{3}H]di-*o*-tolylguanidine and buffer containing (+)-pentazocine (500 nM (+)-pentazocine in TRIS buffer (50 mM TRIS, pH 8.0)) at rt. The non-specific binding was determined with 10 µM non-labeled di-*o*-tolylguanidine. The K_d value of di-*o*-tolylguanidine is 17.9 nM.[5]

5.11. References

1. Bradford, M. M. A rapid and sensitive method for the quantitation of microgram quantities of protein utilizing the principle of protein-dye binding. *Anal. Biochem.* **1976,** *72*, 248–254. DOI: 10.1006/abio.1976.9999.
2. Stoscheck, C. M. Quantitation of protein. *Methods Enzymol.* **1990,** *182*, 50–68. DOI: 10.1016/0076-6879(90)82008-p.

.3. Cheng, Y.; Prusoff, W. H. Relationship between the inhibition constant (K1) and the concentration of inhibitor which causes 50 per cent inhibition (I50) of an enzymatic reaction. *Biochem. Pharmacol.* **1973,** *22*, 3099–3108. DOI: 10.1016/0006-2952(73)90196-2.

4. DeHaven-Hudkins, D. L.; Fleissner, L. C.; Ford-Rice, F. Y. Characterization of the binding of 3H(+)-pentazocine to sigma recognition sites in guinea pig brain. *Eur. J. Pharmacol.* **1992,** *227*, 371–378. DOI: 10.1016/0922-4106(92)90153-m.
5. Mach, R. H.; Smith, C. R.; Childers, S. R. Ibogaine possesses a selective affinity for sigma 2 receptors. *Life Sci.* **1995,** *57*, PL57-62. DOI: 10.1016/0024-3205(95)00301-l.

6. Binding curves of 14 and 13 at κ and related receptors

<u>κ Receptor affinity of **14**</u>

Competitive binding curve 1

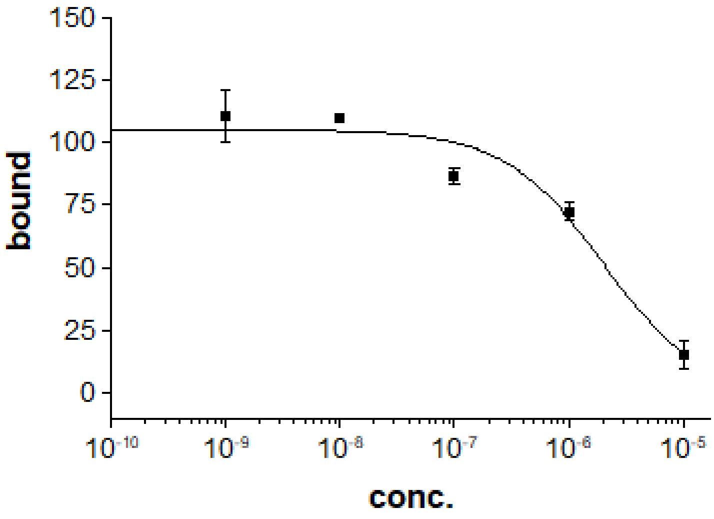

Competitive binding curve 2

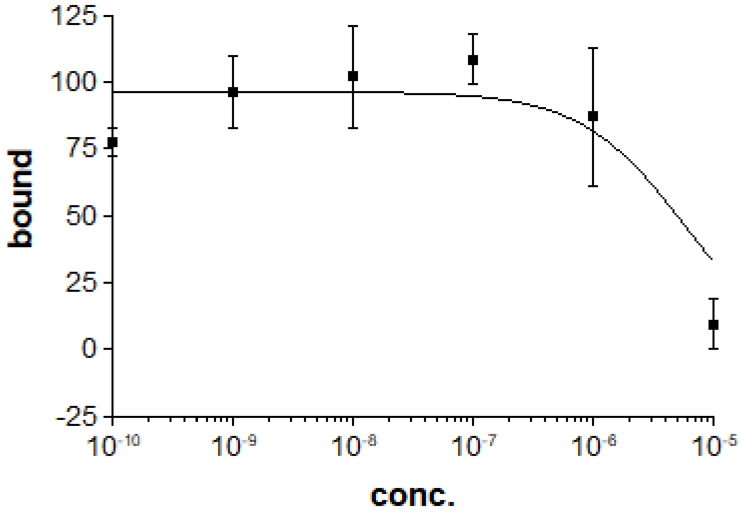

Competitive binding curve 3

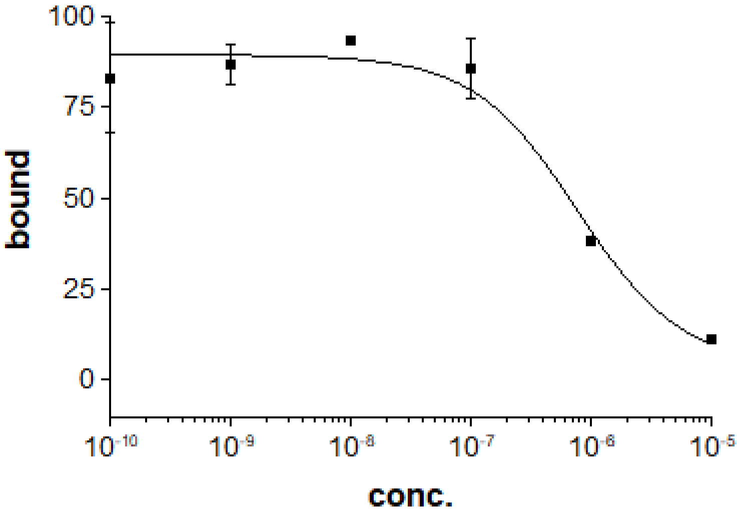

Competitive binding curve 4

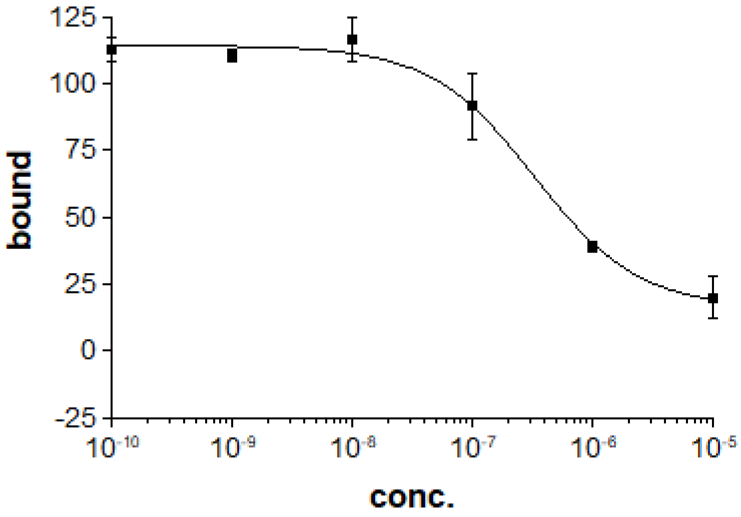

All experiments were conducted with a preparation of guinea pig brain. Compound **14** and the guinea pig brain preparation were incubated at 37 °C for 2 h. The filter B was pretreated with 0.2 % polyethylenimine (PEI) before use.

μ Receptor affinity of **14**

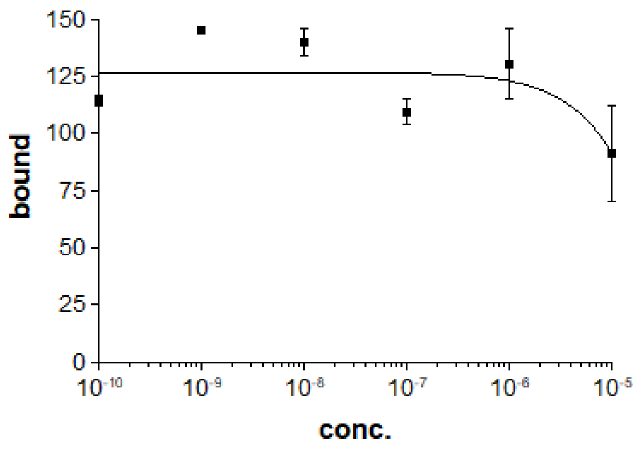

δ Receptor affinity of **14**

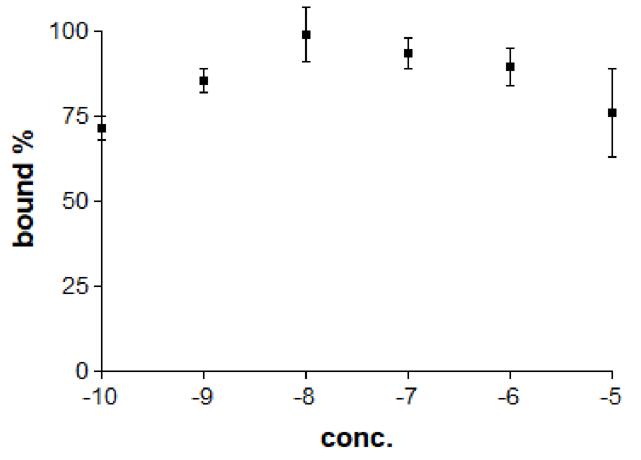

σ_1 Receptor affinity of **14**

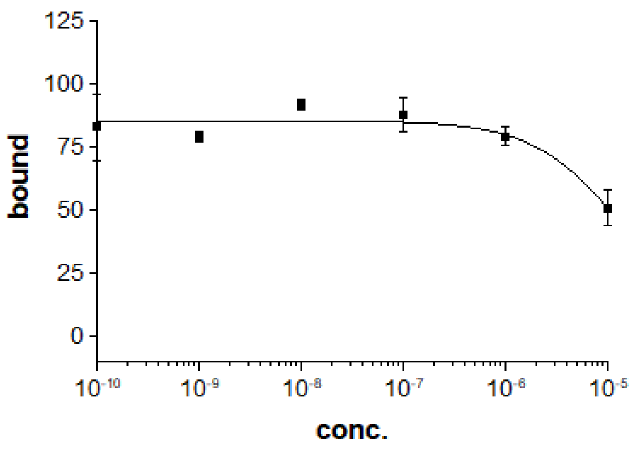

κ Receptor affinity of **13**

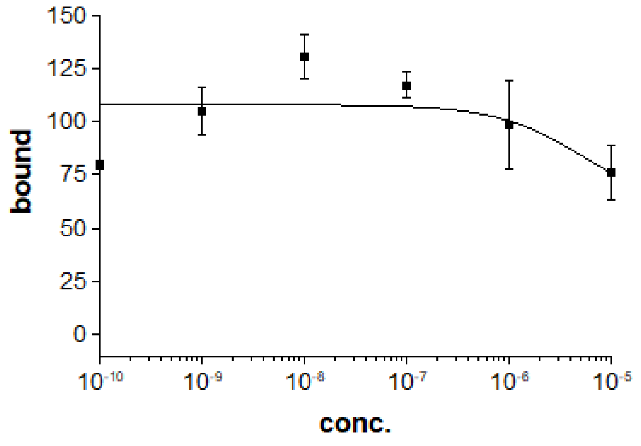

σ_1 Receptor affinity of **13**

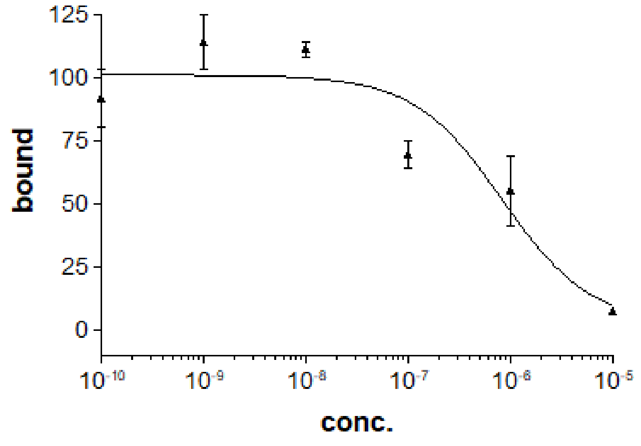

σ_2 Receptor affinity of **13**

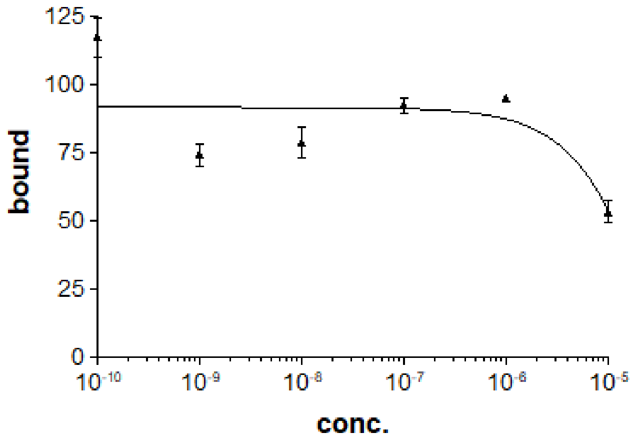

7. ^{1}H and ^{13}C NMR spectra

^{1}H and ^{13}C NMR spectra of **5** in $CDCl_3$

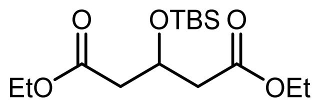

5

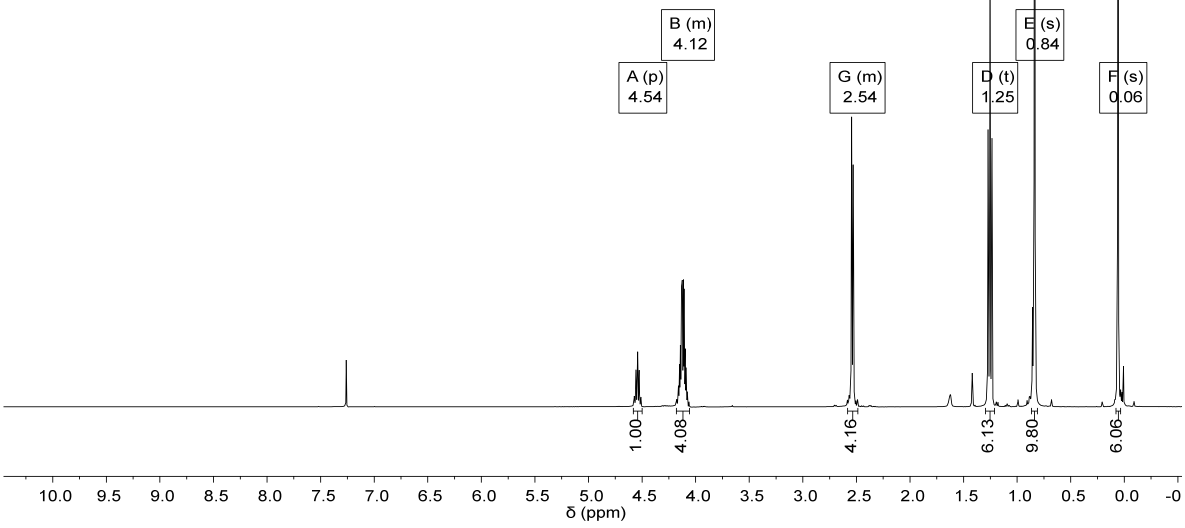

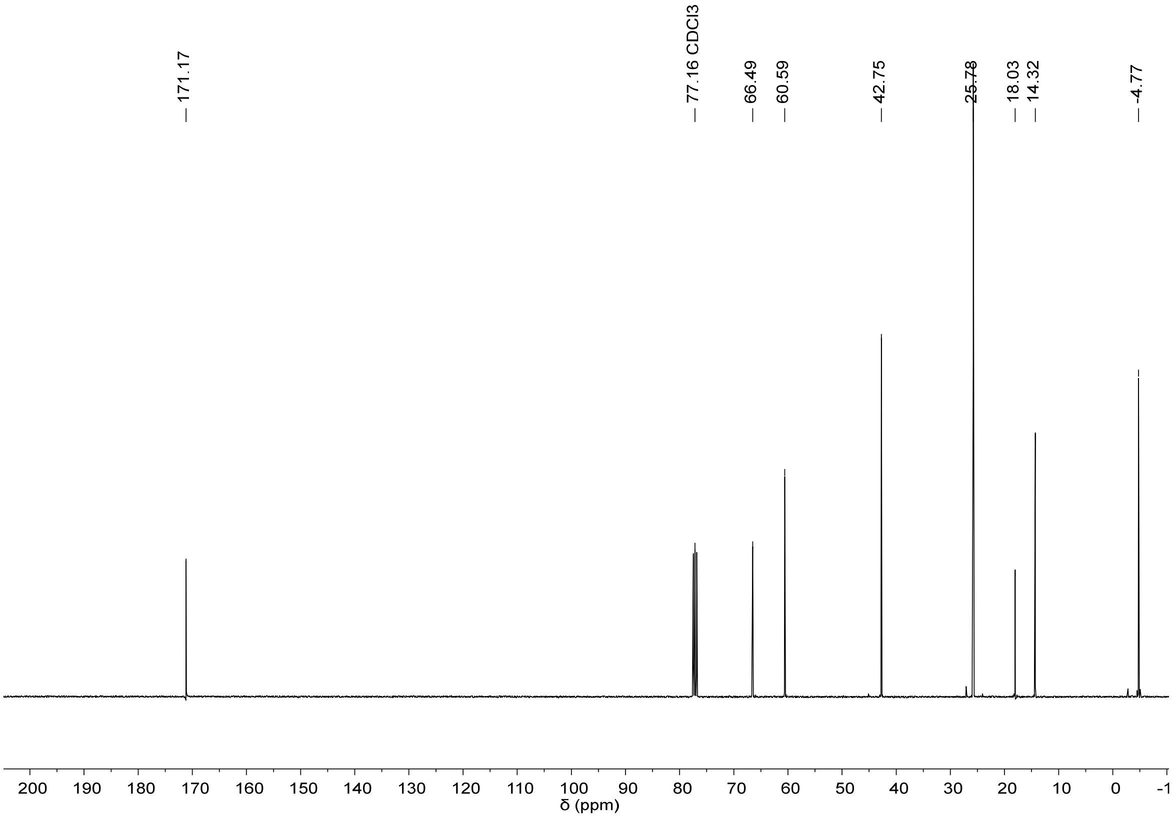

^{1}H and ^{13}C NMR spectra of **6** in $CDCl_3$

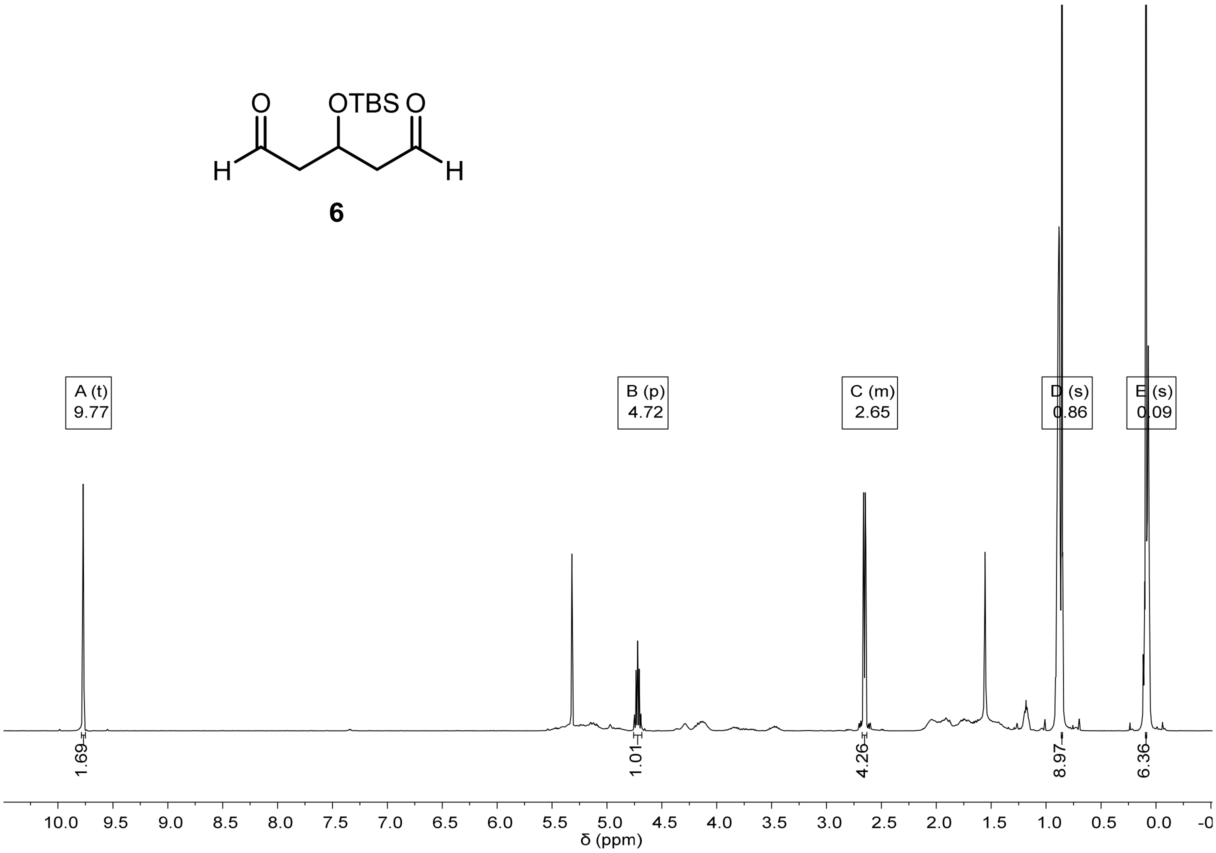

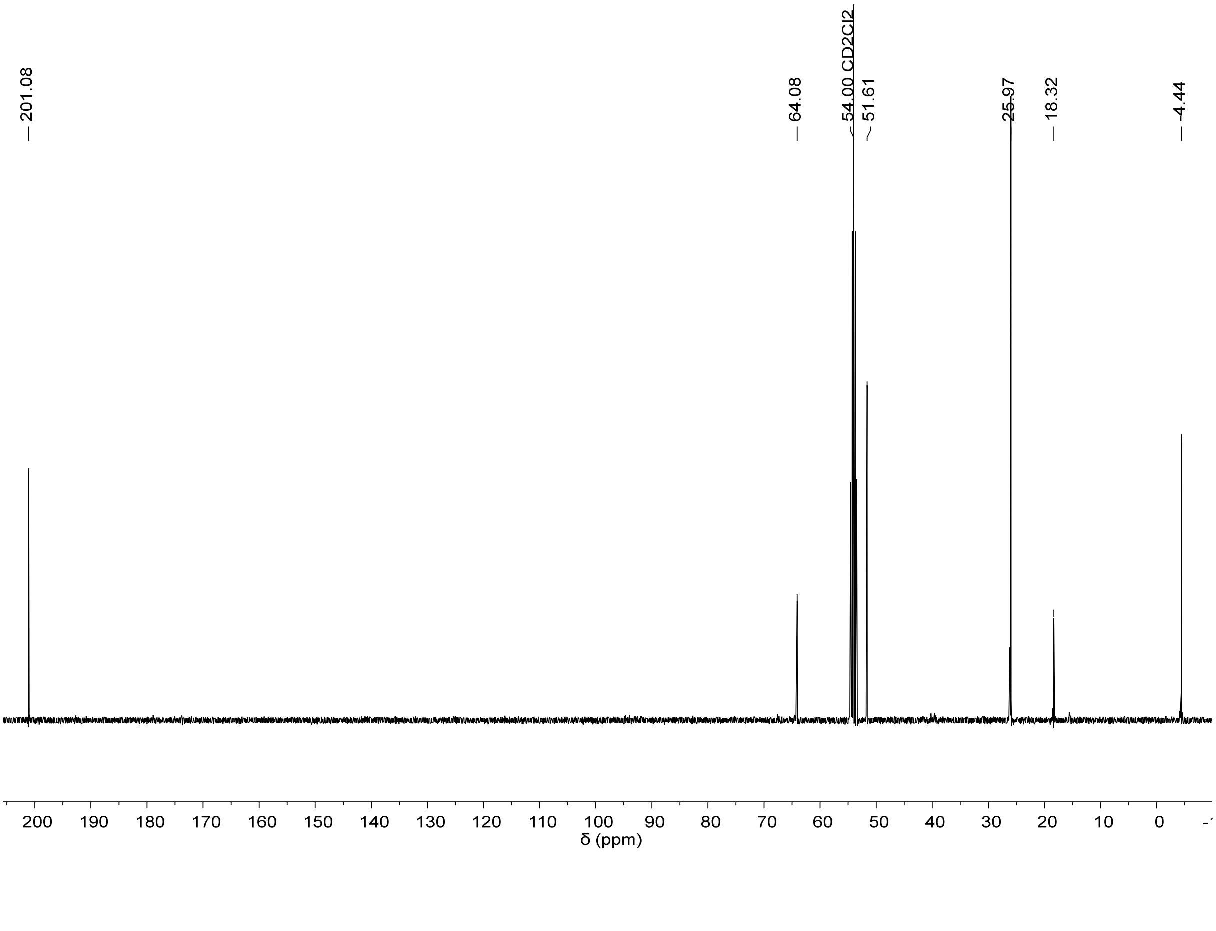

^{1}H and ^{13}C NMR spectra of a 70 : 30 mixture of (2*r*,5*s*)-**7a** and (2*r*,5*r*)-**7b** n $CDCl_3$

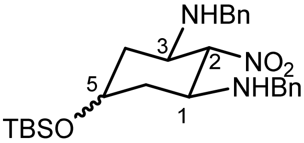

(2*r*,5*s*)-**7a**: OR ax
(2*r*,5*r*)-**7b**: OR eq

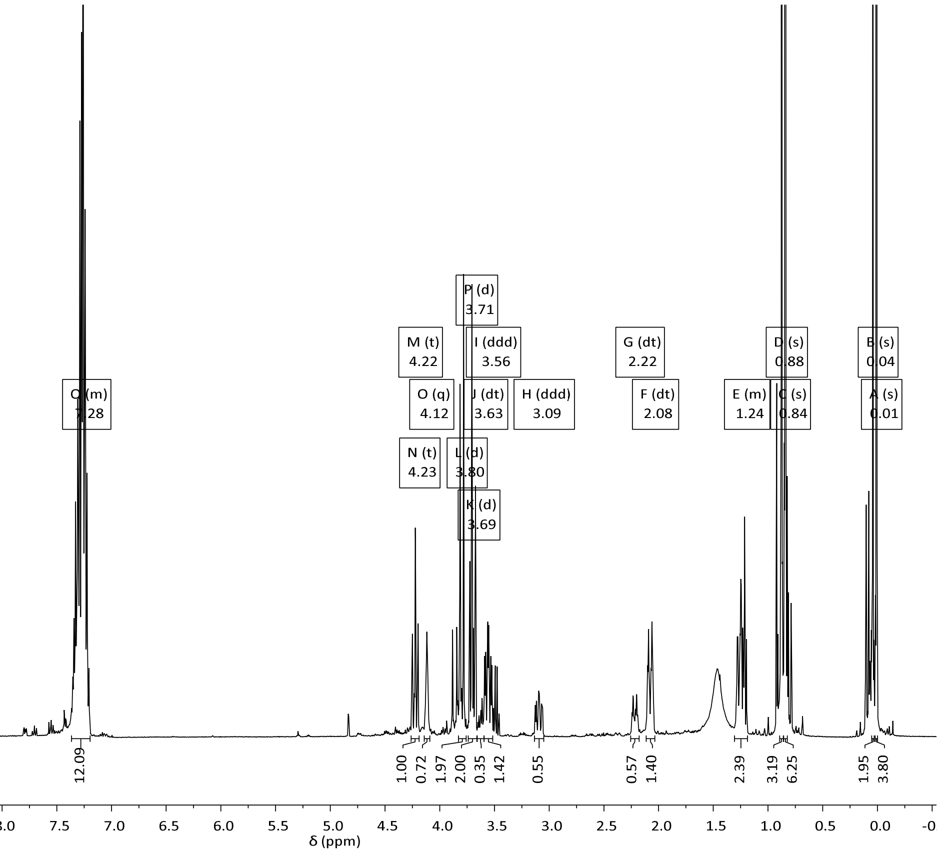

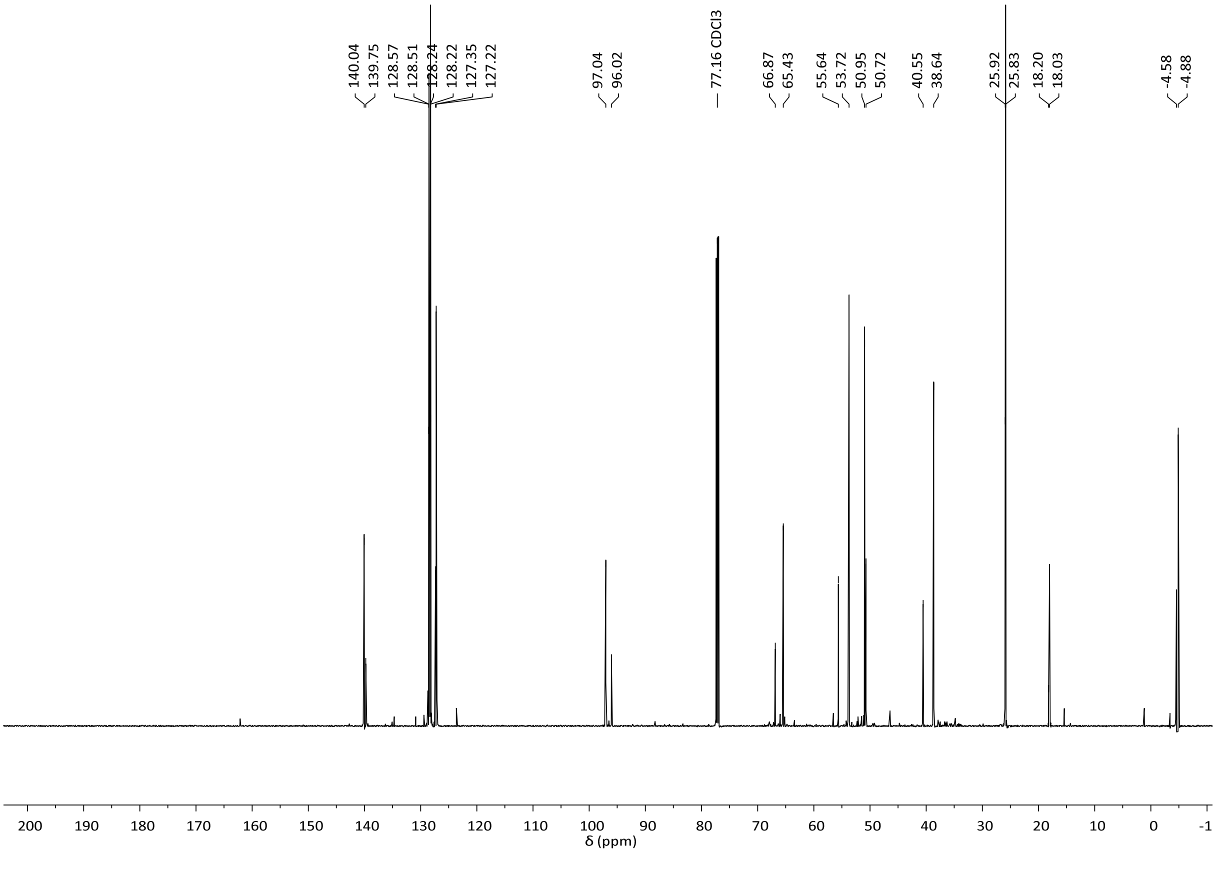

^{1}H and ^{13}C NMR spectra of (2*r*,5*s*)-**7a** n $CDCl_3$

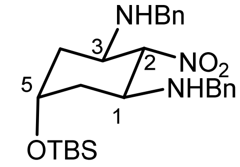

(2*r*,5*s*)-**7a**

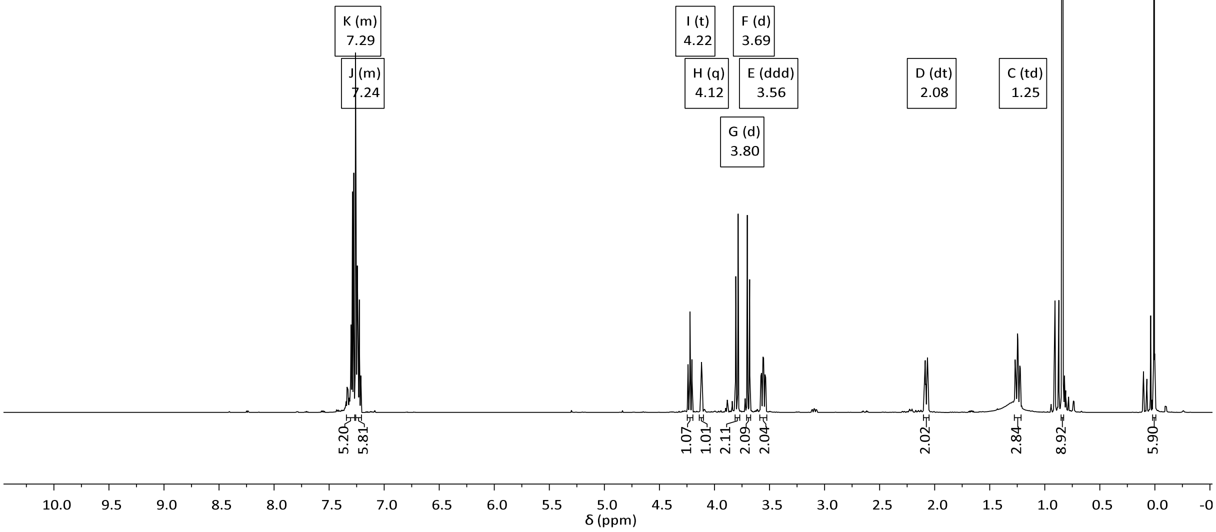

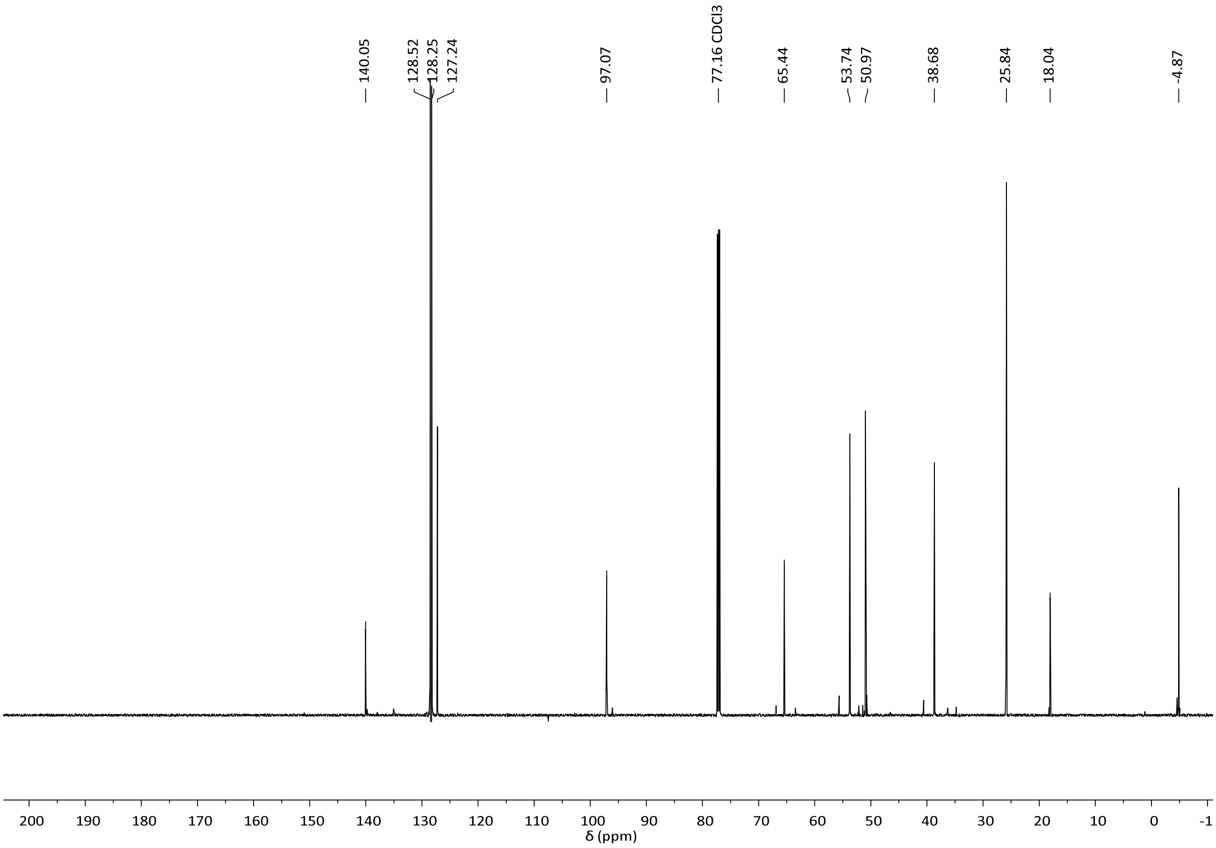

^{1}H and ^{13}C NMR spectra of **8** in $CDCl_3$

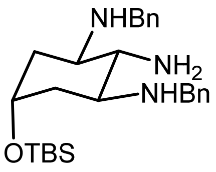

8

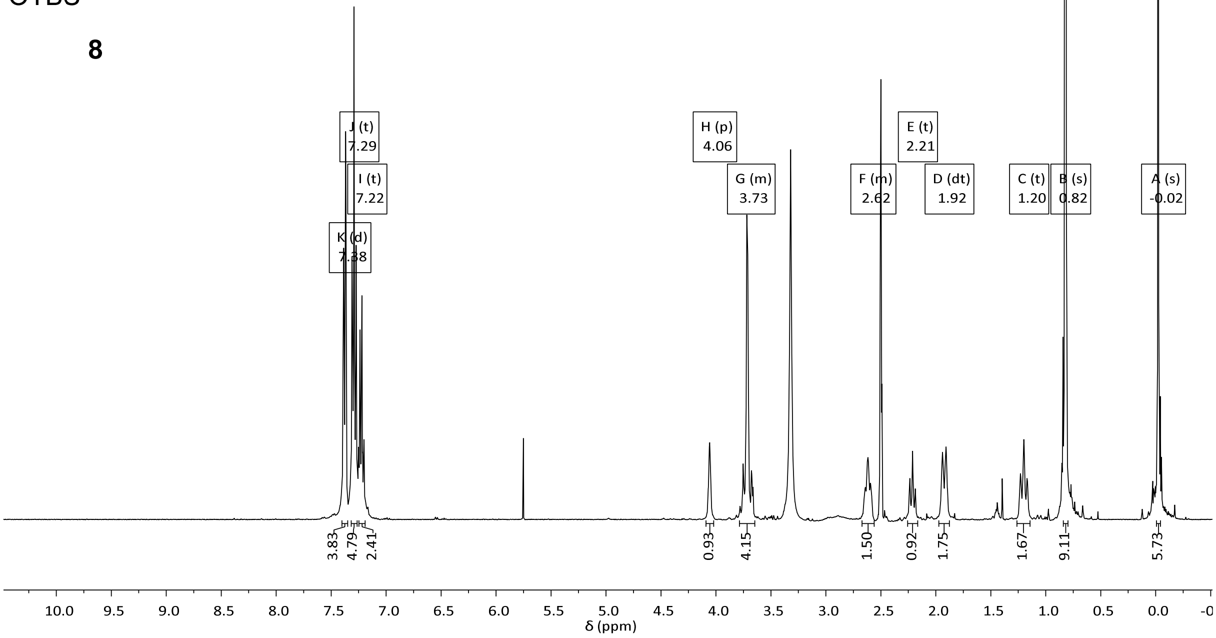

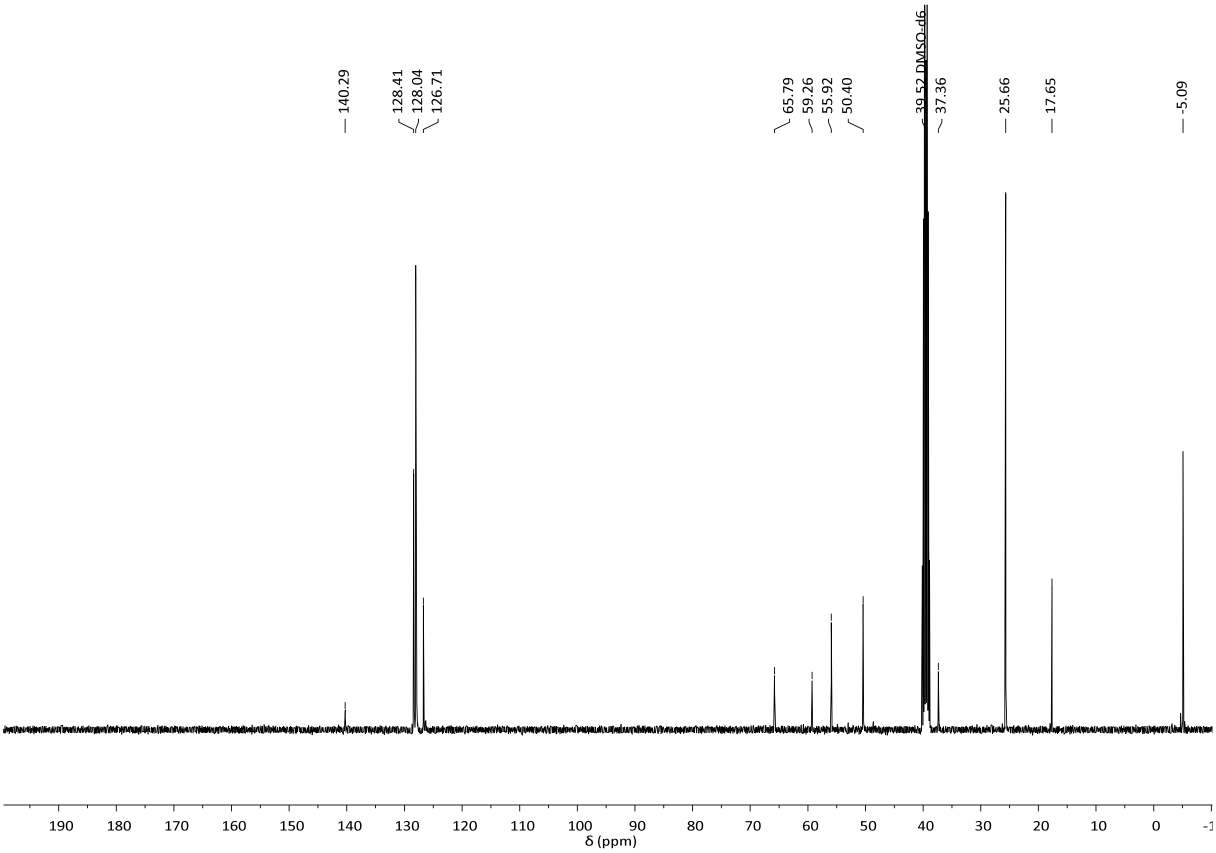

^{1}H and ^{13}C NMR spectra of **9** in $CDCl_3$

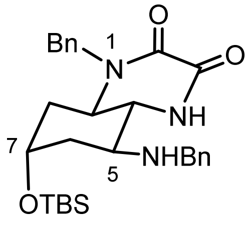

9

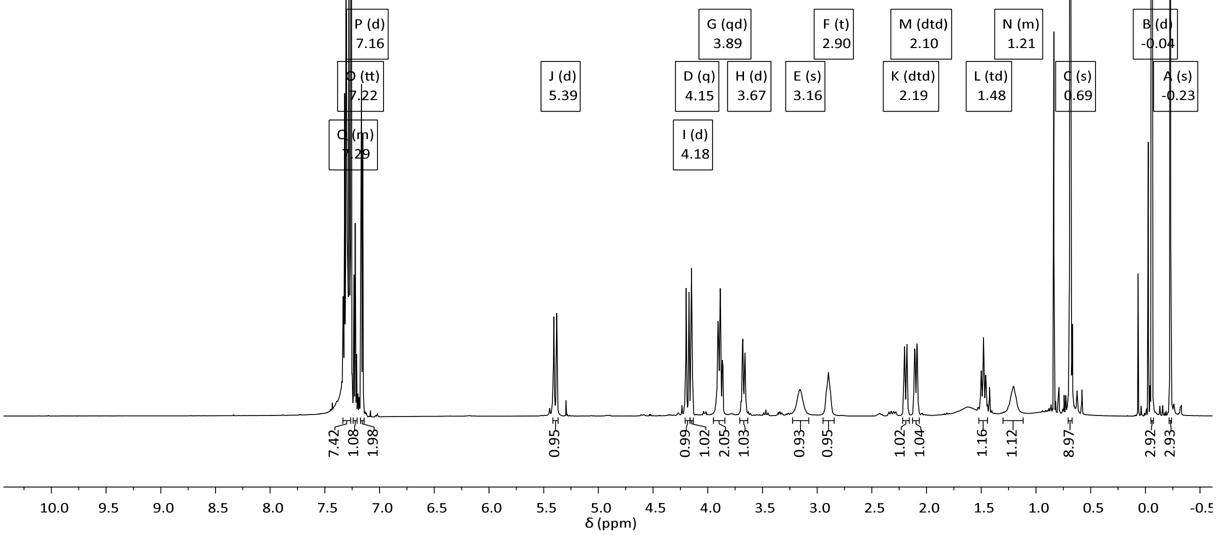

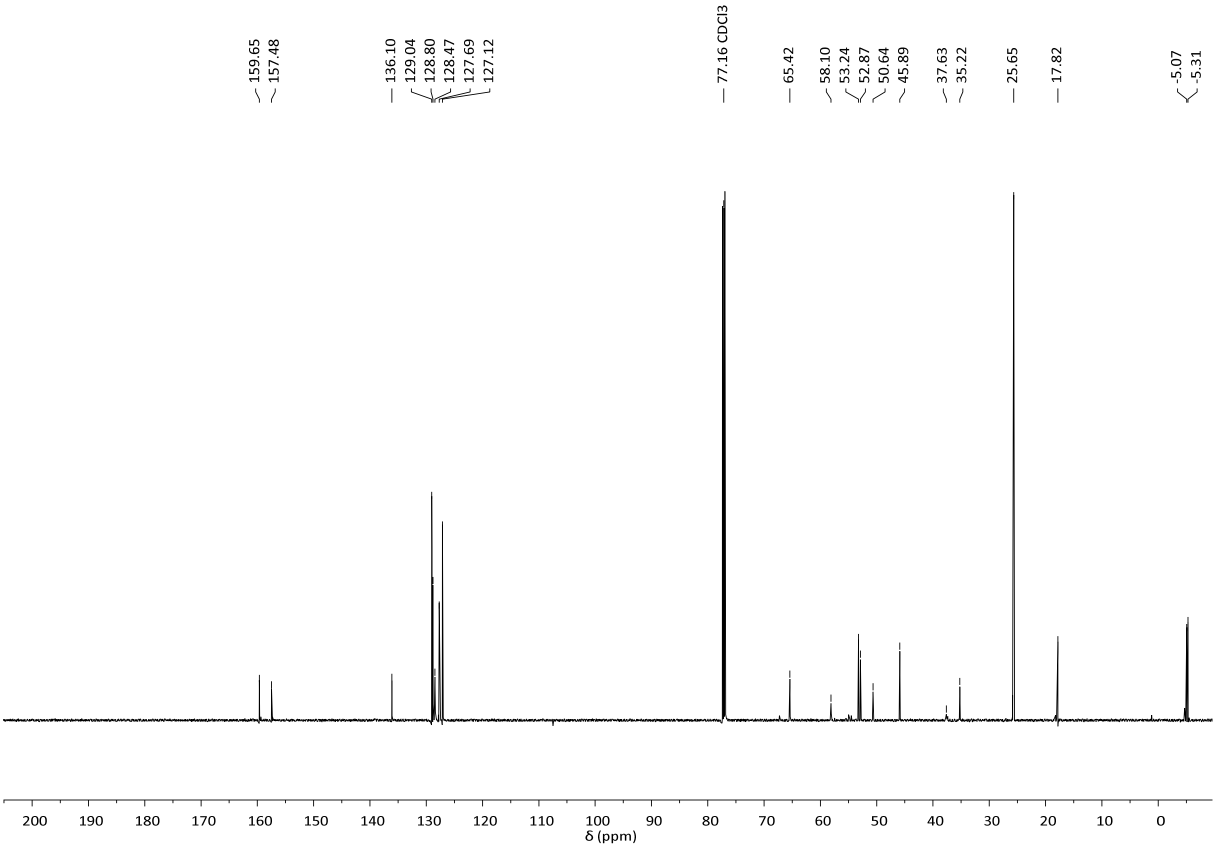

^{1}H and ^{13}C NMR spectra of **10** in $CDCl_3$

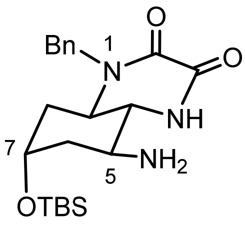

10

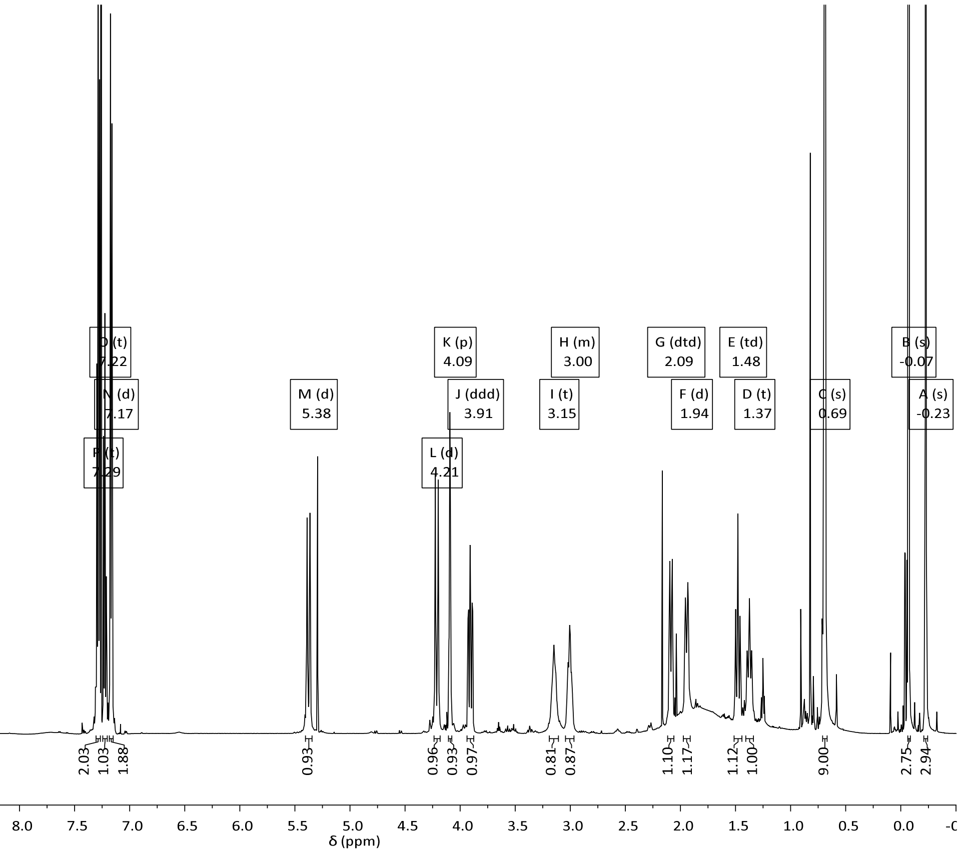

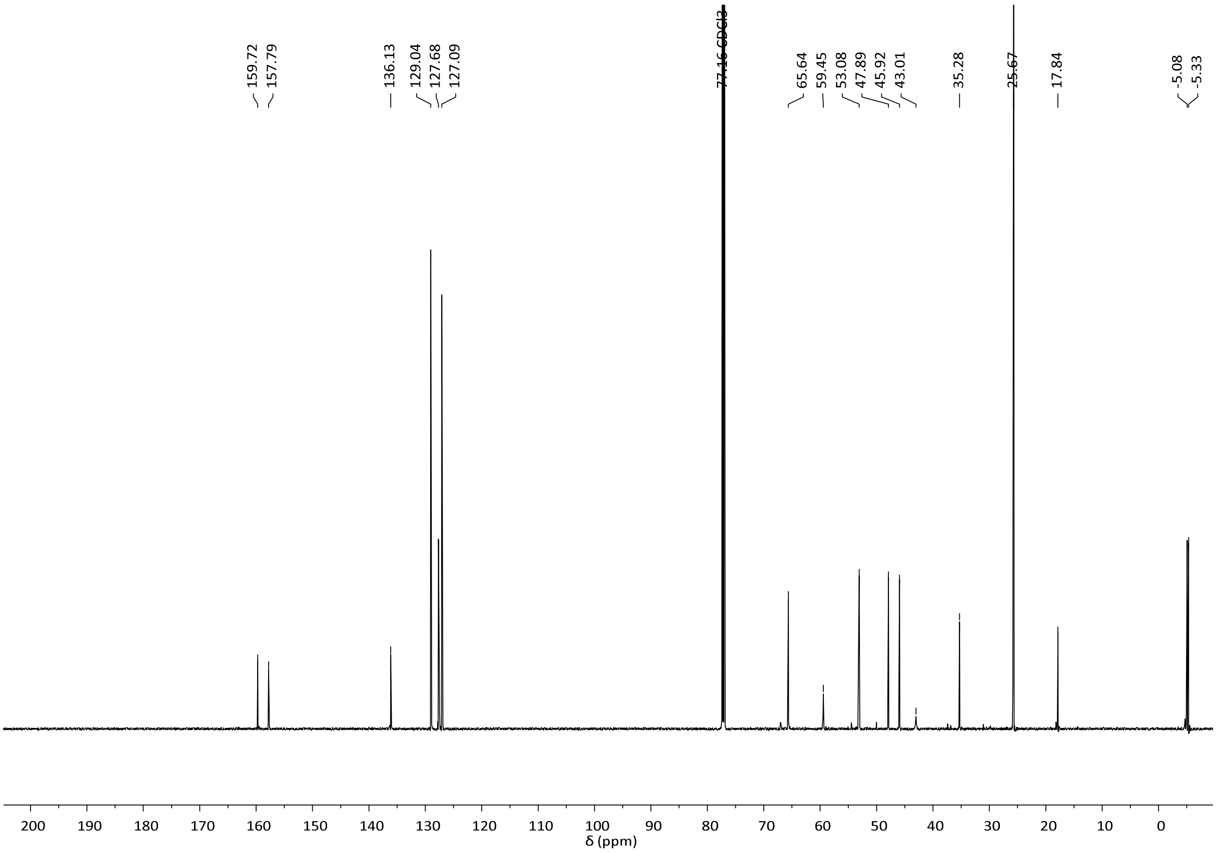

^{1}H and ^{13}C NMR spectra of **11** in $CDCl_3$

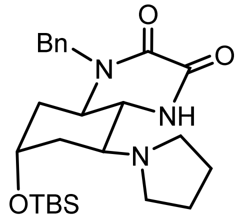

11

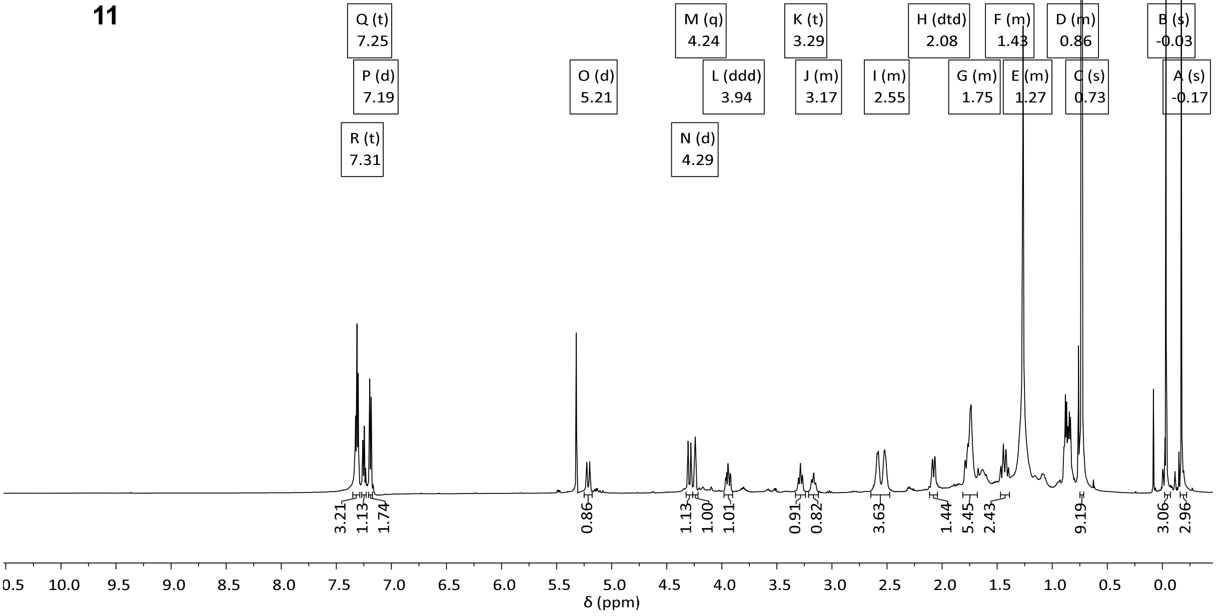

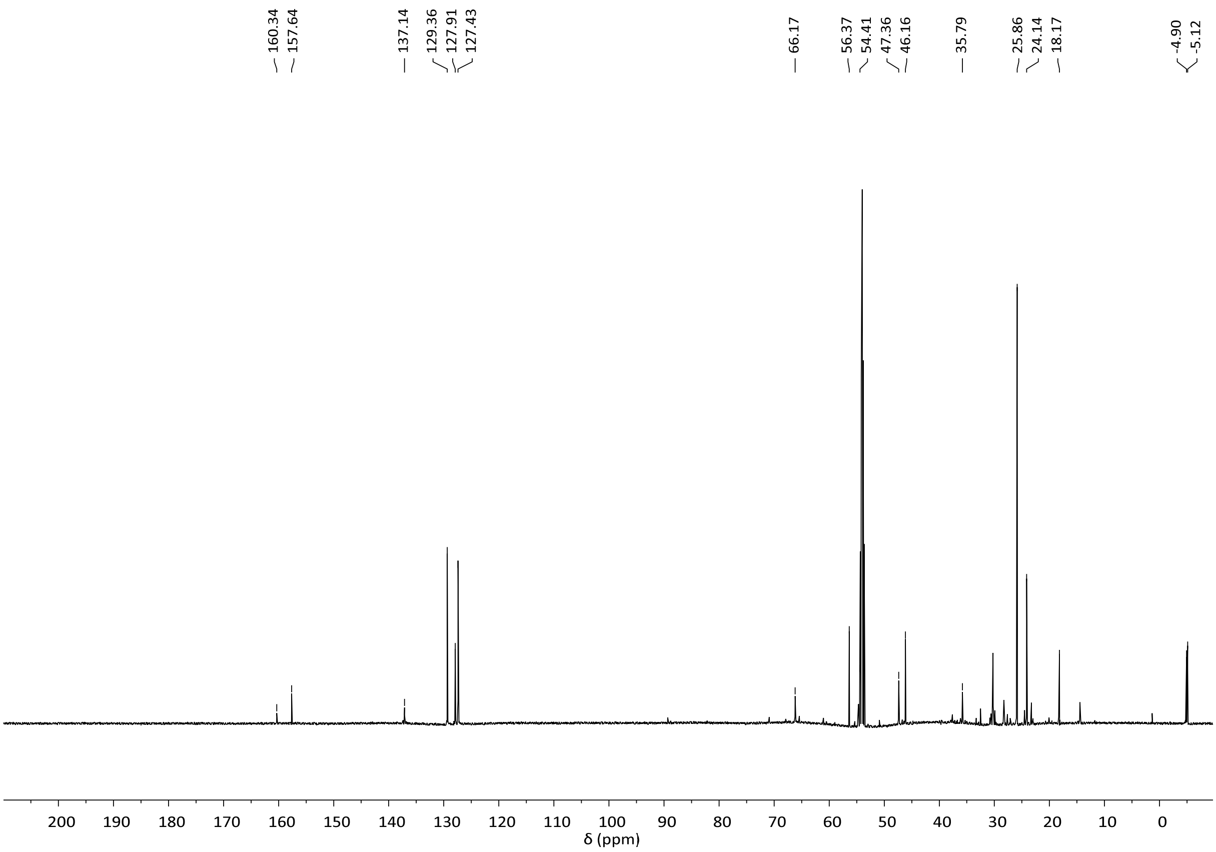

^{1}H and ^{13}C NMR spectra of **13** in $CDCl_3$

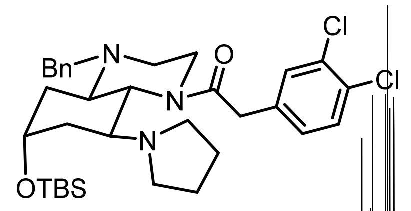

13

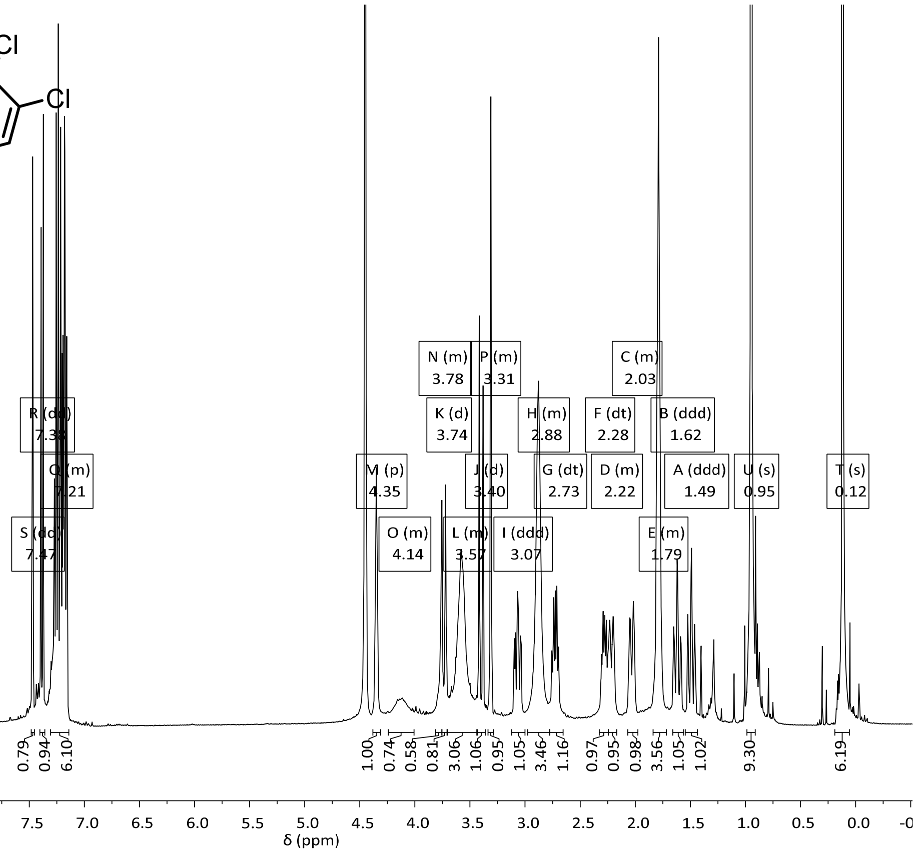

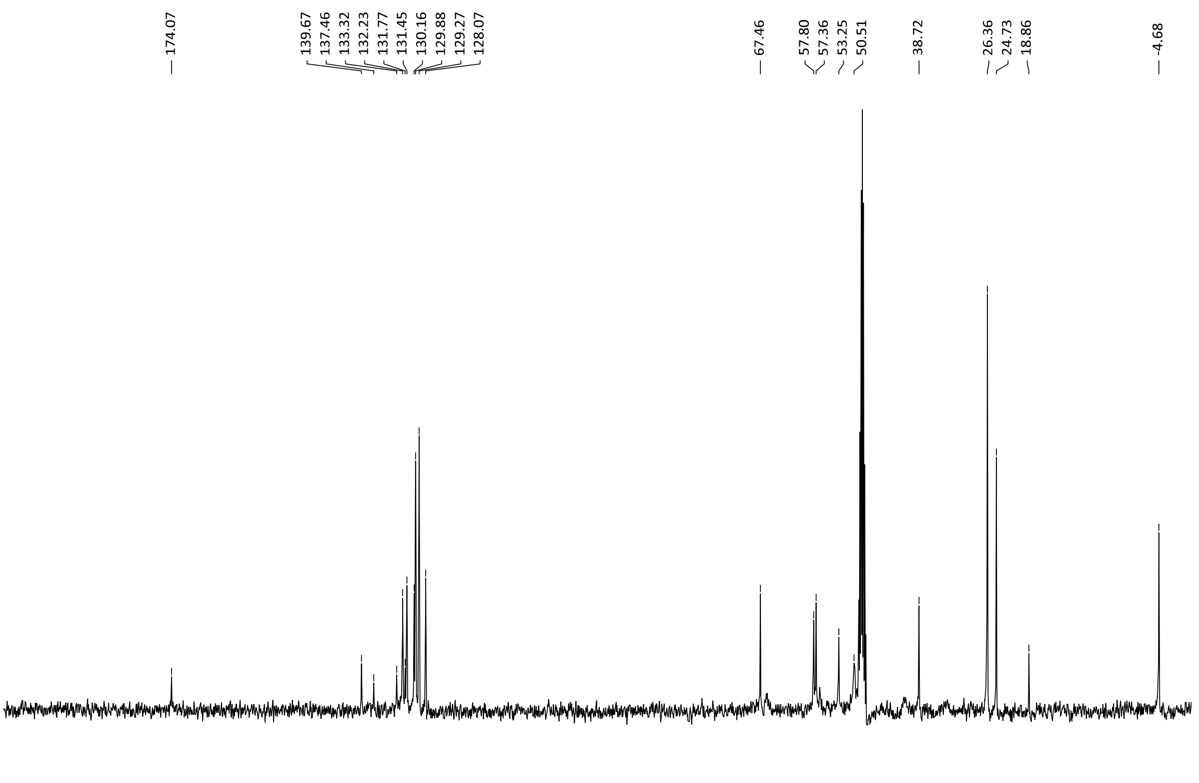

^{1}H and ^{13}C NMR spectra of **14** in $CDCl_3$

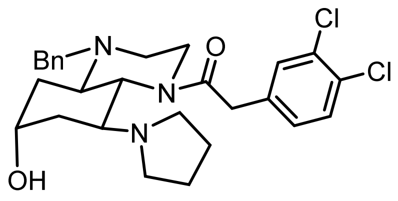

14

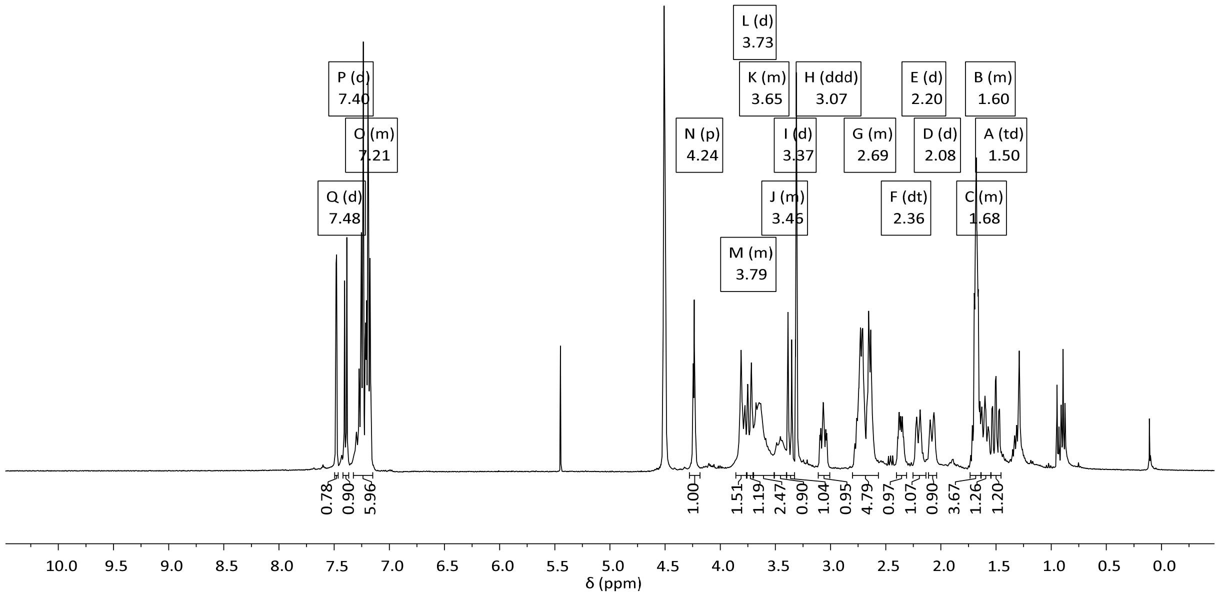

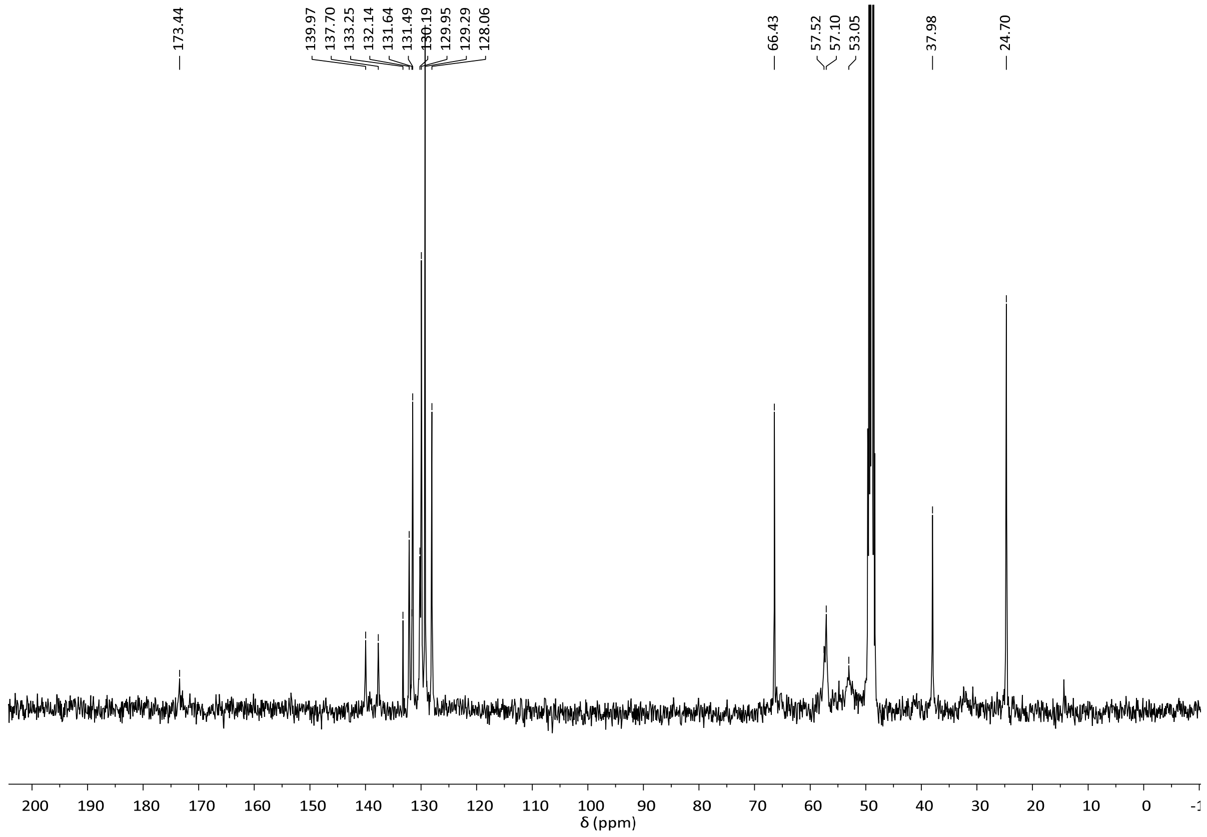

p
